# Navigating the landscape of protein folding and proteostasis: from molecular chaperones to therapeutic innovations

**DOI:** 10.1038/s41392-025-02439-w

**Published:** 2025-10-23

**Authors:** Omer Faruk Kuzu, Lars Jørgen Tvenge Granerud, Fahri Saatcioglu

**Affiliations:** 1 Department of Biosciences, University of Oslo, Oslo, Norway; 2 Institute for Cancer Genetics and Informatics, Oslo University Hospital, Oslo, Norway; 3 Department of Molecular Oncology, Institute of Cancer Research, Oslo University Hospital, Oslo, Norway

**Keywords:** Cell biology, Cancer therapy, Molecular neuroscience, Target identification

## Abstract

Protein folding is a fundamental process ensuring that polypeptide chains acquire the correct three-dimensional structures required for biological function. This complex journey from nascent polypeptides to mature proteins is tightly regulated by the cellular proteostasis network—an integrated system of molecular chaperones, folding enzymes, and degradation machineries. Disruptions in this network lead to dysproteostasis, a pathological state implicated in a growing list of human diseases, including neurodegenerative disorders, metabolic syndromes, and cancer. In this review, we provide a comprehensive and multidimensional analysis of protein folding biology, tracing its evolution from early theoretical foundations to cutting-edge biophysical and computational techniques that now permit near-atomic-resolution modeling of folding dynamics. We explore the historical progression of protein folding research, including landmark discoveries of secondary structure, chaperone biology, and energy landscape theory. We detail the roles of key molecular chaperones across cytosolic, mitochondrial, and endoplasmic reticulum compartments, emphasizing their collaborative actions in protein folding and quality control. We also discuss the multifactorial causes of protein misfolding—from genetic mutations to aging and oxidative stress—and examine the pathological consequences, paying special attention to diseases characterized by toxic protein aggregation and loss of proteome fidelity. We then examine therapeutic innovations targeting proteostasis, including chaperone modulators, proteostasis pathway inhibitors, and emerging strategies to increase proteome resilience. By consolidating insights at the molecular, cellular, and systems levels, this review underscores the central role of protein folding homeostasis in health and disease and highlights novel opportunities for therapeutic intervention through the modulation of the proteostasis network.

## Introduction

Cellular protein homeostasis, or proteostasis, is a cornerstone of normal cellular health and functionality in all living organisms. This term describes the delicate balance between protein synthesis, folding, modification, trafficking, and degradation, ensuring a stable and functional proteome capable of executing the myriad tasks essential for life. From catalyzing metabolic reactions to mediating signal transduction, proteins constitute the molecular machinery driving cellular processes, and their proper folding and function are paramount. The three-dimensional conformation of a protein, which is dictated by its amino acid sequence and influenced by various cellular factors, directly determines its activity and function. Maintaining this precise structural integrity is a continuous challenge in the face of both intrinsic and extrinsic factors that can disrupt the folding process or destabilize already folded proteins, leading to a state of imbalance in the cell known as dysproteostasis.

There are a range of perturbations that result in the loss of proteostasis, including genetic mutations that alter amino acid sequences, errors during protein synthesis and post-translational modifications (PTMs), dysfunction of the molecular chaperones responsible for assisting protein folding, and various cellular stresses, such as heat shock, oxidative stress, and nutrient deprivation. The accumulation of misfolded or unfolded proteins can trigger cellular dysfunction, activate stress response pathways, and ultimately contribute to the development of a wide spectrum of diseases. In neurodegenerative disorders such as Alzheimer’s disease and Parkinson’s disease, metabolic diseases such as type 2 diabetes, and various cancers, disruption of proteostasis is a key pathological determinant. Understanding the complex mechanisms that underlie dysproteostasis, the cellular responses it activates, and the specific ways it contributes to disease pathology is crucial for developing effective therapeutic interventions.

This review provides a comprehensive exploration of the multifaceted nature of protein folding, from its historical roots to its implications for human health. We begin by tracing the historical trajectory of protein folding research, highlighting the key discoveries and milestones that have shaped our understanding. This includes seminal work on protein denaturation, the elucidation of secondary structure elements, and Anfinsen’s thermodynamic hypothesis, providing a foundation for exploring the complexities of protein folding. We then delve into the various factors that can disrupt protein folding, examining the molecular mechanisms by which these perturbations lead to protein misfolding and aggregation. This review also examines cellular stress response pathways, including the heat shock response (HSR) and the unfolded protein response (UPR), which act as critical defense mechanisms against protein folding defects in the cell. These pathways, which are activated by the accumulation of misfolded proteins, initiate a complex cascade of events aimed at restoring proteostasis by enhancing protein folding capacity, increasing the degradation of misfolded proteins, and attenuating protein synthesis.

Finally, we examine the specific roles of dysproteostasis in a range of diseases, with a particular emphasis on neurodegenerative diseases and cancer. Cancer cells, characterized by uncontrolled proliferation and metabolic reprogramming, often experience high levels of proteotoxic stress due to increased protein synthesis and an altered cellular environment. However, they also display a remarkable ability to adapt to and even exploit dysproteostasis for their survival and progression. We discuss the specific mechanisms by which cancer cells manipulate proteostasis networks, including the UPR, to support their growth, metastasis, and resistance to therapy. This review aims to provide a foundation for understanding the underlying causes of protein misfolding and aggregation, examining the molecular mechanisms involved, and exploring potential therapeutic strategies to mitigate their impact on various diseases.

## Historical perspective and milestone events in protein folding

**Fig. 1:**
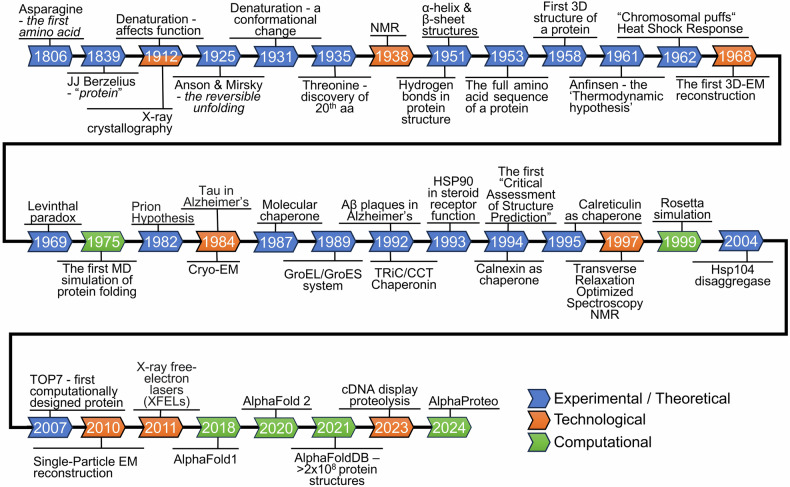
Timeline of key discoveries in protein folding research. Over the last two centuries, protein folding research has seen multiple major discoveries. Some of the central developments in the field are chronologically represented. These enabled the formation of theoretical models that best represent our understanding of the underlying mechanisms. Much of the progress is due to significant technological advancements that were developed and/or implemented along the way

The study of protein folding began in the mid-20th century and has evolved significantly over the past several decades, driven by both experimental and theoretical advancements. This section traces key discoveries and models that shaped our understanding of how proteins attain their functional three-dimensional structures and maintain homeostasis (Fig. 1).

### Early discoveries and foundational work (1800–1950s)

Proteins were first recognized as distinct biomolecules by Berzelius in 1838, with the first amino acid, asparagine, discovered in 1806 and all others discovered by 1935.^1,2^ Fischer and Hofmeister’s 1902 proposal of linear peptide-bonded chains laid the foundation for structural studies.^2^ Chick’s work (1910–1912) distinguished denaturation (loss of structure/function) from coagulation,^3^ whereas Anson and Mirsky (1925) reported reversible unfolding of hemoglobin.^4^ Wu (1931) later linked denaturation to conformational changes, establishing that structure dictates function.^5^

Pauling and Corey’s discovery of α-helices and β-sheets (1951) revealed protein secondary structure principles.^6^ Sanger sequencing of insulin (1953) revealed that proteins have unique sequences.^7^ Kendrew and Perutz’s X-ray crystallography breakthroughs (1958–1960) solved myoglobin and hemoglobin structures, earning the 1962 Nobel Prize and revealing atomic-level protein architecture.^8,9^ In the 1960s, the work of Christian Anfinsen on ribonuclease A demonstrated spontaneous refolding, leading to the principle that the native structure of a protein is determined solely by its amino acid sequence and represents the most thermodynamically stable conformation under physiological conditions.^10^ This “thermodynamic hypothesis” became foundational but left unresolved questions about folding pathways.

Levinthal’s 1969 paradox highlighted that random conformational sampling is implausible.^11^ Ptitsyn’s framework model (1973) proposed early secondary structure formation,^12^ whereas Karplus and Weaver’s diffusion-collision model (1979) emphasized the formation of transient multimicrodomain intermediates during the folding pathway.^13^ Although both models posit that early local structure formation is critical, the diffusion‒collision model emphasizes the role of molecular diffusion and collision events in assembling the complete fold. Moreover, collisions may also occur between microdomains that are not adjacent in the linear sequence, shifting focus from the individual amino acids that make up the polypeptide chain to the characteristics and interactions of microdomains.^14^ This model, refined and validated throughout the 1980s, provided a mechanistic explanation for rapid folding without exhaustive conformational searches.^13,14^

The late 1970s and 1980s saw the development of new experimental techniques that revolutionized protein folding research. Circular dichroism (CD) spectroscopy, fluorescence spectroscopy, and nuclear magnetic resonance (NMR) spectroscopy have emerged as powerful tools for probing protein structure and dynamics. In particular, the application of NMR to protein studies, pioneered by Kurt Wüthrich and others, enabled the exploration of protein structures in solution and the observation of folding intermediates, providing critical insights into the dynamic nature of this process.^15^

In 1995, the “nucleation‒condensation model” was introduced as a refinement to the “diffusion‒collision model”.^16,17^ Rather than requiring the independent formation of all secondary structures prior to assembly, this model suggests that a small region, formed through a combination of local and long-range interactions, rapidly attains a native-like conformation. This nucleus then serves as a condensation center that drives the cooperative formation of the remaining structure, effectively reducing the conformational search space.

The 1990s introduced energy landscape theory, framing folding as a funnel-guided process where native states occupy energy minima.^18,19^ The ruggedness of the folding landscape—arising from the presence of partially folded states and misfolded conformations—accounts for the heterogeneity and complexity observed in folding pathways. The formation of a native-like nucleus, as posited by the nucleation‒condensation model, corresponds to the initial descent down the funnel, where the entropic cost of organizing the chain is minimized by the formation of local interactions. Once this nucleus is established, the funnel-shaped landscape guides the rapid and cooperative assembly of the remaining structure despite the presence of kinetic traps.

In the early 2000s, Englander and colleagues proposed the “foldon model,” which introduces foldons—independently folding units within a protein.^20^ According to this model, proteins fold in a modular, hierarchical manner, with discrete segments attaining native-like conformations before being assembled into the complete structure. This modular organization reduces the overall conformational search space and facilitates the cooperative assembly of the folded protein, aligning rapid folding kinetics with the complexity of the final native state. Hydrogen–deuterium exchange and site-directed mutagenesis experiments provided evidence supporting the existence of foldons, indicating that certain segments stabilize early in the folding process, whereas others complete their structural formation later.^21^

### Molecular chaperones: Orchestrating protein folding in the cellular milieu (1990s-present)

As our understanding of protein folding has increased, it has become evident that intracellular conditions—such as macromolecular crowding, physiological temperatures, and rapid translation rates—present challenges that increase the risk of protein misfolding and aggregation.^22^ To counteract these challenges, cells utilize molecular chaperones: proteins that assist in proper folding, prevent aggregation, refold misfolded proteins, and aid in protein transport and degradation, thereby maintaining proteostasis.

The discovery of molecular chaperones originated from studies on cellular stress responses. In 1962, Ferruccio Ritossa reported chromosomal “puffs” in the salivary glands of heat-shocked fruit flies, indicating increased expression of specific genes encoding heat shock proteins (HSPs).^23^ Initially perceived as mediating general stress responses, the functional significance of HSPs became clearer through subsequent research.

In 1973, Georgopoulos and colleagues reported that mutations in the *groE* gene of *E. coli* led to protein aggregation, suggesting the role of specialized proteins in facilitating proper folding.^24^ In 1980, Barraclough and Ellis provided additional evidence for this concept by showing that a binding protein in chloroplasts assists in the assembly and folding of Rubisco, a key enzyme in photosynthesis.^25^ Further evidence emerged in 1978 when Laskey et al. introduced the term “molecular chaperone” to describe the role of nucleoplasmin in preventing histone aggregation during nucleosome assembly.^26^ John Ellis expanded this definition in 1987 to include proteins that assist in the correct folding and assembly of other polypeptides without becoming part of the final structure.^27^ This was followed by the finding that purified chaperones could prevent the aggregation of unfolded polypeptides and facilitate their refolding in a Mg-ATP-dependent manner, highlighting the active role that chaperones play in protein folding.^28^

These findings showed that protein folding often requires assistance to prevent misfolding and aggregation. Molecular chaperones are highly conserved across species, highlighting their fundamental role in proteostasis. Dysfunction of chaperones has been linked to diseases such as Alzheimer’s disease and Parkinson’s disease, which are characterized by protein misfolding and aggregation. As discussed below in Section “Molecular chaperones: Facilitators of protein folding dynamics”, molecular chaperones are now recognized not only for their role in protein folding but also for their involvement in protein transport, degradation, and signaling pathways.

### Evolution of experimental techniques in protein folding research

Protein folding research has evolved significantly, driven by continuous advances in experimental techniques. These tools have enabled high-resolution probing of protein structure, dynamics, and energetics. While comprehensive overviews are available elsewhere,^29^ here, we briefly highlight the major methods and recent developments.

The structural exploration of proteins began with X-ray crystallography. Starting in the 1950s and 1960s, with the structures of myoglobin and hemoglobin,^9^ it provided atomic-level insights. Despite its power, X-ray crystallography is limited to static, crystalline snapshots that may not reflect native conformations, and not all proteins crystallize easily. To overcome such limitations, CD spectroscopy emerged in the 1960s–70 s, enabling rapid estimation of secondary structure content.^30,31^ The development of stopped-flow CD later allowed the capture of fast structural transitions that were previously inaccessible.^32^ Simultaneously, fluorescence spectroscopy and Förster Resonance Energy Transfer (FRET) became essential for monitoring conformational changes.^33^ FRET, introduced in the 1970s, functions as a “spectroscopic ruler,” measuring nanometer-scale distances to probe folding mechanisms.^34^

Since the late 1980s, NMR spectroscopy has transformed the field, enabling the study of proteins in solution or membrane-like environments.^15^ The development of multi-dimensional NMR techniques, such as two-dimensional (2D) NMR, has enabled the study of larger proteins and more complex structures in solution or in lipid environments under near-native conditions.^35^ Unlike crystallography, NMR captures the dynamic behavior of proteins, making it especially valuable for examining intrinsically disordered proteins (IDPs) and fold-switching proteins that do not adopt a single stable structure.^36^

Further improvements to X-ray crystallography came with synchrotron radiation in the 1970s–80 s, increasing resolution and speed.^37^ Around the same time, early foundations were laid down for cryo-electron microscopy (cryo-EM) and single-molecule techniques such as optical tweezers, although their application to folding studies matured only in the 1990s, when several pivotal techniques emerged.^38,39^ Cryocrystallography improved X-ray resolution by reducing radiation damage through cryogenic cooling.^40^ Single-molecule FRET (smFRET) enabled real-time tracking of folding at the individual molecule level, revealing conformational heterogeneity.^41,42^ In the meantime, a major leap in NMR came with the introduction of TROSY (transverse relaxation optimized spectroscopy) in 1997, allowing studies of larger proteins by mitigating signal broadening.^43^ Moreover, fluorescence correlation spectroscopy (FCS) advanced the analysis of protein dynamics at low concentrations and fast timescales.^44^

By the early 2000s, atomic force microscopy (AFM) enabled the mechanical manipulation of single proteins, the mapping of energy landscapes and rare folding events.^45,46^ In parallel, advancements in hydrogen-deuterium exchange mass spectrometry (HDX-MS) and native MS have provided high-resolution data on protein stability and conformational dynamics.^47^ The 2010s marked a turning point for cryo-EM with direct electron detectors and improved image processing.^48,49^ This revolutionized 3D structural reconstruction at near-atomic resolution, capturing folding intermediates and dynamic complexes. Serial crystallography using XFELs further expanded time-resolved structural studies, revealing protein motion on femtosecond timescales.^50^ Moreover, real-time NMR techniques have been developed and track folding pathways as they occur, whereas integrative approaches—combining multiple methods—have become central in the study of protein structure and dynamics.^51^

In addition to direct folding studies, methods such as deep mutational scanning and display technologies (yeast, phage, ribosome) have emerged to evaluate the functional impact of mutations.^52–54^ These approaches use large variant libraries and high-throughput selection to map sequence‒function relationships, which is particularly useful for protein engineering and understanding fitness landscapes. Most recently, cDNA proteolysis has enabled large-scale quantification of folding stability.^55^ By combining cell-free systems with next-generation sequencing, researchers have assessed the thermodynamic stability of nearly one million protein variants in just one week. This technique offers insights into side chain thermodynamic couplings and generates rich datasets for protein design and machine learning models for folding prediction.

### Experimental detection of protein aggregation and misfolding

In addition to structural techniques for probing protein structure and folding, a variety of chemical agents and imaging strategies have been developed to detect misfolded proteins and aggregates. These approaches, while often less structurally detailed than biophysical methods, offer highly practical tools for monitoring protein aggregation kinetics and studying the pathogenic roles of misfolded proteins in disease states.

Dyes such as Congo Red (CR) and Thioflavin T (ThT) remain among the most widely used tools for detecting amyloid fibrils and other β-sheet-rich aggregates.^56,57^ These dyes exploit the structural hallmarks of aggregated proteins, including extended cross-β architectures, to produce characteristic optical signatures. The CR binds to β-sheet regions via hydrophobic and electrostatic interactions, yielding distinctive green birefringence under polarized light.^58^ However, its labor-intensive staining protocols and limited sensitivity for early aggregates have motivated the search for alternative methods.^56^

ThT offers a fluorescence-based approach, displaying strong signal enhancement upon binding to amyloid fibrils, likely due to restricted intramolecular rotation.^56^ ThT is widely used to monitor amyloid formation kinetics, although its sensitivity for detecting early-stage oligomers is limited.^59^ Structurally related dyes, including thioflavin S, and newly developed small-molecule probes with higher affinities for soluble oligomers, such as BD-Oligo and TAMAOP derivatives, have broadened the toolkit for studying early aggregation events.^57^

More recent efforts have introduced luminescent conjugated oligothiophenes (LCOs), such as p-FTAA and hFTAA, which exhibit solvatochromic behavior (a reversible change in the absorption or emission spectrum of these probes that is induced by the action of solvents) and spectral shifts depending on the aggregation state or target protein.^60,61^ Thiophene-based dyes with specificity for tau aggregates, such as bTVBTs and pTP-TFE, are particularly valuable in the study of tauopathies.^62,63^ Near-infrared (NIR) probes and dual-emission dyes have further advanced real-time, in vivo imaging capabilities, offering deeper tissue penetration and reduced background fluorescence.^64,65^

In clinical research, radiotracers derived from ThT, such as Pittsburgh compound B (PiB), pioneered noninvasive positron emission tomography (PET) imaging of amyloid deposits in the brain.^66^ Subsequently, 18F-labeled tracers, including florbetapir and florbetaben, which have improved imaging protocols and are now used routinely for Alzheimer’s disease (AD) diagnostics, were developed.^67–69^ Similar approaches have been applied to tau pathology, with first-generation tau PET tracers such as flortaucipir providing valuable but sometimes nonspecific imaging.^70^ Second-generation tracers, including RO948 and PI-2620, aim to increase specificity, although off-target binding remains a challenge.^71^ Promising candidates such as SNFT-1 are now emerging with improved sensitivity and reduced off-target effects for tau imaging.^72^

Together, these methods complement advanced structural techniques by enabling the detection, quantification, and in vivo visualization of protein aggregates, bridging molecular insights with disease-relevant pathology.

### Computational and theoretical approaches: simulating folding processes

In addition to experimental techniques, computational and theoretical methods have been central to elucidating the complex problem of protein folding—from early conceptual models to recent state-of-the-art AI-driven predictions that achieve near-atomic resolution.

Initial efforts in the mid-20th century employed simplified lattice models, notably the hydrophobic–polar (HP) model, which yielded fundamental insights into hydrophobic collapse and the role of energy landscapes in guiding folding.^73^ These foundational approaches paved the way for molecular dynamics (MD) simulations in the 1970s, which utilized detailed force fields such as CHARMM and GROMOS to model atomic-level folding pathways.^74^ By the late 20th century, advancements, including all-atom force fields, solvent models, enhanced sampling techniques (replica exchange, metadynamics), and distributed computing projects such as Folding@home, enabled MD simulations to explore larger systems and longer timescales.^75,76^ Subsequent refinements, including coarse-grained models and GPU acceleration, have further improved the efficiency and scalability of computational folding studies.^77,78^

A transformative breakthrough in computational methods for protein folding occurred with the advent of deep learning. Early machine learning efforts assisted in clustering conformational landscapes and analyzing simulation data.^79,80^ Later, techniques such as support vector machines and decision trees were applied to predict structural features from sequence data via known protein structures.^81^ However, a defining milestone was achieved with AlphaFold, which first participated in CASP13 in 2018 and revolutionized the field with AlphaFold2 at CASP14 in 2020.^82^

AlphaFold2 introduces a transformer-based architecture that integrates attention mechanisms and evolutionary couplings to predict heavy-atom coordinates from sequences alone with near-experimental accuracy.^82^ Prior to AlphaFold2, only approximately 17% of the human proteome had experimentally determined structures. Following its release, the publicly available AlphaFold protein structure database expanded structural coverage to over 200 million proteins, spanning nearly all known sequences, with built-in confidence scores that also enable identification of intrinsically disordered regions.^83^

Building on this success, other deep learning models, such as RoseTTAFold, ESMFold, and OmegaFold, have emerged, offering diverse strengths, from rapid single-sequence predictions to modular frameworks capable of incorporating user-defined constraints.^84–86^ Extending beyond single-protein predictions, AlphaFold-Multimer addresses protein complexes with improved interface predictions, which are especially effective for coevolved complexes but are currently limited in antibody‒antigen interactions.^87,88^ To address many of these limitations, AlphaFold3 leverages diffusion-based neural networks to model complex biomolecular assemblies, markedly enhancing predictions for protein–ligand, protein–nucleic acid, and antibody–antigen interactions.^89^ Similarly, complementary tools such as RoseTTAFold All‑Atom also extend predictions to heterogeneous biological complexes, bringing “color” to the “black‑and‑white” of prior protein-only models.

Despite the remarkable progress in AI-driven structure prediction, current models predominantly generate static conformations, leaving protein dynamics—including transient interactions, conformational ensembles, and intrinsically disordered regions—less well characterized. In this respect, AI models function as highly accurate “black boxes” for structure prediction but not for folding mechanisms. Therefore, traditional computational methods—MD simulations and physics-based coarse-grained models—are essential for investigating dynamic folding pathways and mechanistic insights. Recent innovations, such as the Wako-Saitô-Muñoz-Eaton (WSME-L) model, incorporate long-range interactions and disulfide bonding, rapidly generating interpretable folding landscapes and mechanisms that are consistent with experimental observations.^90,91^

Furthermore, with this wealth of unprecedented knowledge, protein design has entered the spotlight. AlphaProteo, a recently introduced AI‑driven system, moves beyond prediction to generate novel, high‑affinity protein binders.^92^ By leveraging extensive databases (including AlphaFold‑predicted structures), preliminary results from AlphaProteo have demonstrated remarkable success rates and significant improvements in binding affinities, opening new avenues for drug design and therapeutic development.

AI‑based predictions now routinely guide experimental design: static models provide blueprints for crystallography, cryo‑EM, and NMR, whereas MD and physics‑based simulations refine our understanding of protein dynamics and folding mechanisms. The feedback loop between computations and experiments is rapidly accelerating progress in structural biology, protein engineering, and drug discovery. Looking ahead, the trend is toward unified frameworks that predict not only a single static structure but also an entire conformational ensemble, accounting for dynamics, multicomponent interactions, and even co-translational effects. As AI methods converge with enhanced MD simulations and refined theoretical models, we approach a future where the complete biophysical behavior of proteins—including misfolding and functional modulation—can be predicted with high fidelity.

In summary, while early computational tools provided the theoretical foundation for understanding protein folding, the last five years—especially the last two—have seen an unprecedented transformation through AI. This revolution, together with evolving physics-based approaches, is not only enabling accurate structural predictions but also paving the way toward a deeper, mechanistic understanding of protein folding—and its rational manipulation for biomedical and biotechnological applications. As we better interpret these AI models, we might extract new principles of protein folding. A key emerging direction is the integration of structure prediction with the cellular context. Proteins fold in vivo within crowded, dynamic environments; future AI models will take this into account and may incorporate currently ignored biological constraints, enabling prediction of folding pathways as polypeptides emerge from the ribosome or modeling the effects of intermolecular interactions on folding efficiency and misfolding prevention.

## Molecular chaperones: facilitators of protein folding dynamics

Molecular chaperones are essential for maintaining protein homeostasis by assisting in the correct folding of proteins, preventing aggregation, and facilitating the degradation of misfolded proteins. They are broadly categorized on the basis of their molecular weight, structure, and function. Major families include heat shock proteins (HSP40, HSP60, HSP70, HSP90, and HSP100), small HSPs (sHSPs), and chaperonin-containing TCP-1 (CCT) complexes. A detailed classification, including their specific functions, cellular localization, oligomeric forms, and mechanisms of action, is summarized in Table 1.

**Table 1:** Classification and functional overview of human molecular chaperone families

Family	Genes	Oligomeric form	Core mechanism	Compartment(s)	Functions/Targets
HSP70 (17 members)	HSPA1A/1B/1 L/2/4/4 L/5–9/12 A/12B/13/14; HSPH1; HYOU1	Monomer (forms transient complexes)	ATP-dependent binding and release cycle for hydrophobic client regions	Cytosol, ER (BiP), mitochondria (mtHSP70/Grp75)	Nascent chains, stress-unfolded proteins, translocating polypeptides; prevents aggregation; co-translational folding and protein import, ERAD
HSP90 family (5 members)	HSP90A-A1/B1/A3P; HSP90B1;TRAP1	Obligate dimer	ATP-driven conformational clamp for client maturation	Cytosol, ER (GRP94), mitochondria (TRAP1), nucleus	Kinases, hormone receptors, transcription factors; and assists in late folding
Small HSPs (11 members)	HSPB1-11	Dynamic oligomers (dimers → 24+mers)	ATP-independent holdase, dynamic binding of misfolded proteins	Cytosol, mitochondria, nucleus and other organelles	Stress-denatured proteins, amyloidogenic proteins; prevents aggregation, aids in cytoskeletal stability, acts in oxidative stress
HSP40 family (49 members)	DNAJA1-4; DNAJB1-14 (except 10); DNAJC1-30; DNAJC5B/G	Monomer (JC) or dimer (JA and JB)	Co-chaperone of HSP70; substrate targeting and ATPase stimulation	Cytosol, ER, mitochondria, nucleus, and other organelles	Client delivery to HSP70, diverse isoform-specific functions including suppression of polyQ aggregation (DNAJB6), targets proteins for proteasomal degradation
HSP110	HSPH1	Monomer	NEF for HSP70; also has disaggregase activity	Cytosol	Key component in disaggregase triad with HSP70 and DNAJA1/B1
Chaperonins	HSPD1/E1	HSPD1: two stacked heptameric rings; HSP10: 7-mer	Encloses substrates for ATP-driven folding cycles	Mitochondrial matrix	Mitochondrial matrix proteins, folding imported proteins
	CCT1-8/6B (TRiC/CCT)	Two stacked octameric rings	Encloses substrates for ATP-driven folding cycles	Cytosol	Actin, tubulin, WD40 domain proteins, complex clients with β-propeller structures
	CLPB (HSP100 AAA⁺)	Double hexamer	ATP-dependent disaggregase	Mitochondrial intermembrane space	Misfolded or aggregated mitochondrial proteins
NEFs	BAG1–6, HSPBP1, SIL1, GRP170	Monomers or dimers	Catalyze ADP → ATP exchange on HSP70; regulate client release	Cytosol (BAG1–6), ER (SIL1, GRP170), nucleus	Controls duration of HSP70 binding; regulates decision between folding and degradation

**Fig. 2:**
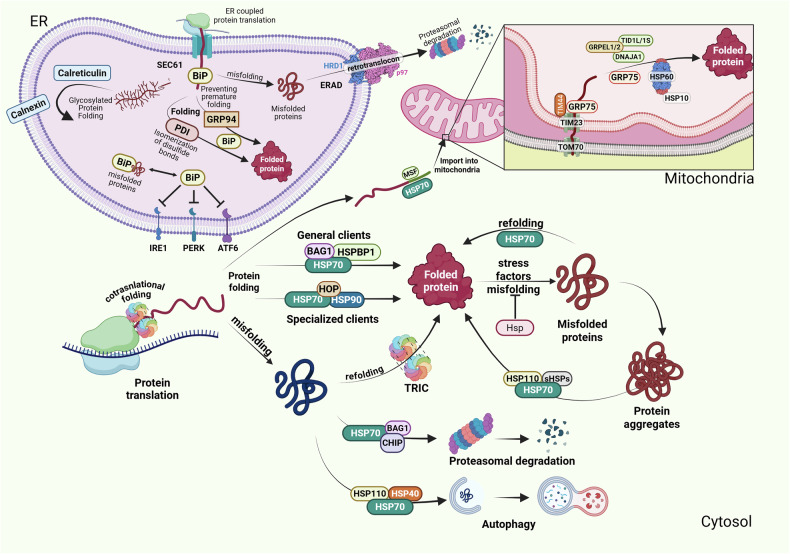
Protein folding, quality control mechanisms, and import into cellular organelles. Protein folding in the ER, mitochondria, and cytosol is tightly regulated by chaperones, co-chaperones and other regulatory proteins to ensure proteostasis. In the cytosol, newly synthesized proteins undergo folding with the assistance of molecular chaperones such as HSP70, which facilitates general protein folding with the aid of co-chaperones like BAG1 and HSPBP1. Some specialized clients require additional processing by HSP90, while others undergo folding via TRiC (TCP-1 Ring Complex). Misfolded proteins that cannot be refolded are targeted for proteasomal degradation or autophagy, with HSP110, small HSPs, and disaggregases playing key roles in aggregate clearance. In the ER, protein synthesis is coupled with folding, mediated by chaperones such as BiP (HSP70 family member), which prevents premature folding and regulates the UPR via IRE1, PERK, and ATF6. ERAD clears irreversibly misfolded proteins through retrotranslocation to the cytosol and proteasomal degradation. Folding of glycosylated proteins is assisted by calreticulin and calnexin, while GRP94 (HSP90 family member) and PDIs facilitate proper disulfide bond formation and stabilization. Mitochondrial protein import is mediated by the TOM/TIM complexes, with GRP75 facilitating the translocation into the mitochondrial matrix where proteins subsequently achieve their functional conformation with the aid of mitochondrial chaperones HSP60 and HSP10

In the following subsections, we discuss the key members of these chaperone families in greater detail, highlighting their specialized roles and functional interplay during protein folding in specific cellular compartments (Fig. 2).

### Cytosolic folding and triage hub

#### HSP70

The HSP70 family members are highly conserved and central to protein folding, refolding, and translocation across membranes.^93,94^ They represent up to 5% of total cellular proteins even under normal conditions.^95^ Human HSP70 family members, encoded by 16 genes, exhibit distinct localizations and functions. For example, HSPA8 (HSC70) is constitutively expressed in the cytosol, whereas HSPA1A/B (HSP70) is stress inducible. Organelle-specific isoforms include HSPA5 (BiP/GRP78) in the ER and HSPA9 (mtHsp70/mortalin/GRP75) in the mitochondria.

Structurally, HSP70 proteins contain two main domains: an N-terminal nucleotide-binding domain (NBD) and a C-terminal substrate binding domain (SBD).^93,94^ ATP binding and hydrolysis in the NBD drive conformational changes in the SBD, modulating its affinity for substrates. The SBD consists of a β-sheet region that captures hydrophobic stretches of unfolded proteins and an α-helical lid that controls substrate binding and release. ATP-bound HSP70 has lower substrate affinity, facilitating release, whereas hydrolysis to ADP stabilizes substrate binding, promoting protein folding.^96^

A flexible linker region transmits allosteric signals between the NBD and SBD, coordinating substrate interactions with ATP hydrolysis cycles.^97^ Co-chaperones, especially HSP40 members (J-domain proteins), stimulate ATP hydrolysis, enhance substrate binding, and facilitate client delivery to HSP70.^98^ Conversely, nucleotide exchange factors (NEFs), such as BAG1 (BCL-2-associated athanogene 1) and HSPBP1 (HSP Binding Protein 1), promote ADP release and ATP rebinding, resetting the HSP70 cycle for new substrate interactions.^98^ Since ADP release is often the rate-limiting step, NEFs play a central role in accelerating HSP70 turnover. By enhancing nucleotide exchange, NEFs facilitate timely substrate release and rebinding. Additionally, some NEFs, such as BAG1 and HSP110, link HSP70 to downstream pathways: BAG1 contains a ubiquitin-like domain that can recruit the proteasome, coupling failed folding attempts to protein degradation.

A critical function of HSP70 in cellular proteostasis is in making critical triage decisions regarding misfolded proteins—specifically, whether to attempt refolding or to target them for degradation (Fig. 2).^99^ HSP70 does not actively “discern” between repairable and irreparable proteins; rather, the fate of a substrate is largely determined by how long it remains bound to HSP70. When a misfolded protein engages HSP70, the co-chaperone CHIP (C-terminus of HSP70 Interacting Protein)—which functions as an E3 ubiquitin ligase—stochastically interacts with the HSP70–substrate complex.^99,100^ Because CHIP is present at a relatively low abundance (with estimates suggesting that only approximately 1–10% of HSP70 complexes are bound by CHIP at any given time), only a subset of substrates are ubiquitinated. The probability of ubiquitination increases with the duration of the substrate’s association with HSP70—that is, if the protein fails to achieve its proper conformation quickly, it remains bound longer and thus becomes more likely to be tagged for proteasomal degradation. In this way, CHIP-mediated ubiquitination is biased toward proteins that persistently misfold, serving as an intrinsic triage decision mechanism. Moreover, this process is fine-tuned by additional layers of regulation. Co-chaperones such as HOP and BAG1 promote protein folding by facilitating the transfer of certain substrates from HSP70 to HSP90.^101^ In contrast, CHIP competes with these co-chaperones for binding to HSP70 and directs bound substrates toward degradation.^99^ The mutual exclusivity of these interactions is due to overlapping binding sites on HSP70, particularly within its C-terminal EEVD motif, which is recognized by the tetratricopeptide repeat (TPR) domains of these co-chaperones.^98,102^

Post-translational modifications provide additional regulatory complexity to HSP70 activity by modifying chaperone interactions and substrate affinities.^103^ For example, phosphorylation at Thr636 of HSP70 shifts the substrate fate from degradation to refolding.^104^ Similarly, the AMPylation of ER-resident HSP70 (BiP) at Thr518 reduces its activity under non-stress conditions, whereas stress-induced deAMPylation restores its protein-folding ability.^105,106^ These reversible PTMs enable the dynamic adaptation of chaperone function to changes in the cellular environment.

#### HSP90

HSP90 functions downstream of HSP70, facilitating the maturation of specific client proteins, including kinases and steroid hormone receptors (Fig. 2).^107,108^ It operates as an obligate dimer composed of three conserved domains: an N-terminal domain involved in nucleotide binding (NTD), a middle domain involved in client binding and ATP hydrolysis, and a C-terminal domain (CTD) responsible for dimerization.^107,108^ Eukaryotic HSP90 isoforms also contain a flexible, charged linker between the NTD and middle domain, modulating conformational dynamics and co-chaperone interactions, and a conserved C-terminal MEEVD motif that binds TPR-domain co-chaperones such as HOP, CHIP, and immunophilins.

The HSP90 chaperone cycle is driven by ATP-dependent conformational changes: in the apo-protein or ADP-bound state, the dimer adopts an open, V-shaped conformation; ATP binding induces NTD dimerization, forming a closed clamp around the client protein.^109^ This dynamic “molecular clamp” undergoes twisting and tightening movements that stabilize partially folded clients. Unlike HSP70, which binds short peptide motifs, HSP90 engages clients holistically, threading them through its dimer lumen. In addition, HSP90 can act as a holdase, sequestering aggregation-prone proteins in an ATP-independent manner and buying time for proper folding or degradation.^110^ For further details, please see^109,111^

The transfer of client proteins from HSP70 to HSP90 is typically mediated by the co-chaperone HOP (HSP70-HSP90 Organizing Protein).^107^ HOP acts as a molecular bridge, simultaneously binding to HSP70 and HSP90 through its TPR domains, which specifically recognize the C-terminal EEVD motifs of both chaperones.^102^ This ensures efficient client handover. However, recent findings revealed that HOP is not strictly essential for proteostasis: HOP knockout human cells retain viability and even display improved folding of many HSP90 substrates.^112^ Immunoprecipitation experiments have shown that HSP70 and HSP90 can still interact in the absence of HOP, and only a subset of clients are negatively impacted, suggesting alternative pathways or compensatory mechanisms.^112^ The precise nature and regulation of these alternative pathways remain open research questions.

Conformational transitions of HSP90 are further modulated by co-chaperones. p23 stabilizes the closed ATP-bound state, prolonging the folding window for sensitive clients.^111^ Conversely, AHA1 accelerates ATP hydrolysis by interacting with the NTD and MD, shortening the closed-state dwell time—which is beneficial for some clients but detrimental if prematurely released.^113^ CDC37, a kinase-specific co-chaperone, binds the HSP90 NTD and inhibits ATPase activity while tethering kinase clients, maintaining HSP90 in an open conformation until proper timing for activation is reached.^114^ Collectively, these co‑chaperones fine‑tune the HSP90 clamp, ensuring that each client is released at the appropriate conformational stage for productive maturation—or, if necessary, directed toward degradation.

### Mitochondrial protein import

Mitochondria import ~99% of their proteome from the cytosol, which necessitates a highly regulated system for importing nuclear-encoded proteins into various mitochondrial subcompartments.^115–118^ Cytosolic HSP70, together with the mitochondrial import‑stimulating factor (MSF) and HSP90, recognizes N‑terminal presequences and maintains precursor polypeptides in an unfolded, import‑competent conformation.^118^ These chaperone–precursor complexes dock on the outer‑membrane receptor TOMM70 (translocase of the outer mitochondrial membrane 70), thereby guiding substrates to the translocase of the outer membrane for passage (Fig. 2).^118^

After traversing the outer membrane through the TOM complex, preproteins encounter the TIM23 (translocase of the inner membrane 23) complex, which works alongside the PAM (presequence translocase-associated motor) complex to facilitate their import into the mitochondrial matrix and inner membrane.^116^ Here, another member of the HSP70 family, mtHSP70 (Mortalin/GRP75), takes center stage (Fig. 2).^115,116^ mtHSP70, one of the most abundant proteins in the mitochondrial matrix, possesses a canonical HSP70 family structure but uniquely harbors a 46-residue mitochondrial presequence at its N-terminus and lacks the typical C-terminal EEVD motif.^119^ mtHSP70 binds to incoming preproteins in an ATP-dependent manner, pulling them into the matrix via the TIM23 channel.^116,117^ This energy-dependent translocation process is tightly regulated and facilitated by a coordinated network of co-chaperones. TIM44 anchors mtHSP70 to the TIM23 complex, ensuring its proper localization, whereas HEP1 prevents the aggregation of mtHSP70 and controls its ATPase activity.^116,120^ J-proteins (TID-1L, TID-1S) mediate substrate binding to mtHSP70, while GRPEL1 and GRPEL2 regulate its ATPase activity and strengthen interactions with incoming substrates.^121^ Importantly, while a fraction of mtHSP70 is dedicated to the import process, the majority of these proteins are involved in the folding of newly imported proteins.^93^

Upstream of HSP70s, the HSP40 family of chaperones plays a crucial role in optimizing mitochondrial protein import.^93^ For example, the deletion of Ydj1, the most abundant HSP40 homolog in yeast, significantly hinders this process.^122^ Similarly, in mammalian cells, Ydj1 homologs, such as DNAJA1, support the import of matrix-targeted proteins into mitochondria.^122,123^ The ER-bound J-domain protein Djp1, a yeast HSP40 homolog, directs certain proteins to the mitochondrial outer membrane via an unusual pathway termed ER surface-mediated protein targeting.^124^ Collectively, these cytosolic and membrane‑bound J‑proteins ensure that precursors arrive in the matrix poised for encapsulation by the HSP60/HSP10 system discussed in the following section.

### Mitochondrial protein folding-HSP60/HSP10 chaperonin machinery

After translocation into the matrix, many precursors achieve their native conformation inside the HSP60/HSP10 chaperonin cage (Fig. 2). Human HSP60 (HSPD1) is the mitochondrial member of the group I chaperonin family and works with its co‑chaperonin HSP10 (HSPE1), mirroring the GroEL/ES system first characterized in *E. coli*.^94,125^ Two stacked heptameric HSP60 rings create a barrel that enfolds substrates up to ~60 kDa. Binding of an unfolded chain and ATP triggers HSP10 to cap the cavity; within this isolated, hydrophilic chamber, the client folds and is released after ATP hydrolysis. Multiple rounds can occur, although some substrates mature in a single cycle.^126^

PTMs significantly enhance and regulate the functional versatility of HSP60 by modulating its activity, substrate specificity, and localization [for reviews, see^127,128^]. The phosphorylation of HSP60 at Tyr227 and Tyr243 enhances chaperone activity, thus supporting cancer cell survival and proliferation through the activation of oncogenic signaling pathways.^129^ Nitration of HSP60 under oxidative stress can impair its chaperone function by inducing conformational changes, leading to mitochondrial dysfunction due to the accumulation of misfolded proteins.^127^ Nitrated HSP60 may also be translocated to exosomes for secretion, where it acts as a proinflammatory signal in the extracellular environment.^127^

Intriguingly, the functions of HSP60 extend beyond the mitochondria, with its presence in various cellular compartments, such as the cytosol, plasma membrane, and extracellular environment.^130–132^ These findings challenge the initial notion that HSP60 is solely a mitochondrial protein and suggest a multifaceted role in cellular physiology. Recent work has revealed that, under mild oxidative stress, mitochondrial HSP60 can be actively released into the cytosol through a p38/MK2–MFF1–VDAC–dependent pathway.^133^ Once in the cytosol, HSP60 engages the IκB kinase complex, activating NF-κB–driven transcription of pro-survival genes. Blocking either its release or its interaction with IKK sensitizes cancer cells to oxidative stress and suppresses tumor growth in vivo, underscoring HSP60’s role as a messenger in a mitonuclear survival axis. Moreover, the presence of HSP60 on the cell surface and in the extracellular milieu has been linked with the activation of immune responses, where it acts as a damage-associated molecular pattern (DAMP) and signals to immune cells, thereby influencing inflammatory pathways,^134,135^ which is particularly relevant in understanding autoimmune diseases and cancer.

### Chaperones in the endoplasmic reticulum (ER)

The ER plays a central role in the synthesis, folding, modification, and trafficking of proteins destined for secretion, membrane insertion, or transport to other organelles, constituting approximately one-third of the cellular proteome.^136^ Like mitochondria, the ER houses a complex network of molecular chaperones and folding enzymes that ensure proper protein conformation and function. Misfolded proteins within the ER can trigger stress responses and compromise cell viability.^137,138^

ER chaperones can be grouped on the basis of their function in protein folding and quality control: ATP-dependent chaperones (e.g., BiP/HSPA5/GRP78), lectin chaperones (calnexin, calreticulin), and protein disulfide isomerases (PDIs) (Fig. 2).^139^ These proteins cooperate to prevent aggregation, promote folding, and retain unfolded proteins until proper conformation is achieved.

#### BiP: The central regulator of folding in the ER

BiP (GRP78/HSPA5) is the most abundant luminal chaperone in the ER and the archetypal member of the HSP70 family.^140^ By clamping exposed hydrophobic stretches on nascent or misfolded chains, BiP prevents premature aggregation, assists in folding, and acts as a molecular sentinel that gauges the protein folding load.^141^ Its ATP‑driven open‑and‑closed cycle not only controls substrate binding and release but also modulates the activation state of the three canonical UPR sensors; this couples local proteostatic demand to global transcriptional and translational reprogramming.^142,143^

The versatility of BiP is expanded by six ER‑resident J‑domain proteins (ERdjs) that stimulate its ATPase activity and target it to distinct client proteins.^141^ ERdj1 (DNAJC1) and ERdj2/SEC63 anchor at the Sec61 translocon, synchronizing translation with chaperone availability and actively threading precursors with weak signal sequences into the lumen.^144,145^ ERdj3 (DNAJB11) binds directly to unfolded proteins, facilitates their transfer to BiP, and stimulates the ATPase activity of BiP to enhance its chaperone function.^146^ Persistent non‑native conformers are triaged by ERdj4 (DNAJB9) and ERdj5 (DNAJC10), in addition, ERdj4 escorts aberrant clients to the retro‑translocon and, under stress, attenuates IRE1 signaling.^147,148^ ERdj5, an oxidoreductase of the PDI family, reduces incorrect disulfide bonds to prepare substrates for ER‑associated degradation (ERAD).^149^ This reductase activity prepares misfolded proteins for dislocation from the ER and subsequent degradation by the proteasome in the cytosol. ERdj6 (DNAJC3), also known as p58^IPK^, resides in the ER under normal conditions and binds to both unfolded protein substrates and BiP.^150,151^ During stress, its translocation into the ER becomes less efficient, leading to its accumulation in the cytosol.^150^ In the cytosol, p58^IPK^ inhibits the eIF2α protein kinases PKR, GCN2 and PERK, which are involved in regulating protein synthesis during stress responses.^152–154^ Through this coordinated network of specialized J‑proteins, BiP forms the hub of a dynamic quality‑control grid that links co-translational translocation, luminal folding, disposal of chronic misfolds, and UPR signaling into an integrated proteostasis module.

#### GRP94: ER‑resident HSP90

GRP94 (glucose-regulated protein 94, also known as GP96 or HSP90B1) is the sole Hsp90 paralog in the ER and is specialized for the maturation of a select subset of secretory and membrane proteins, including integrins, Toll-like receptors (TLRs), and insulin-like growth factors (IGFs).^155^ These clients are often large, multidomain proteins whose functional assembly depends on GRP94-mediated conformational refinement and quality control.

Structurally, GRP94 functions as an obligate homodimer composed of three domains per monomer: an N-terminal ATPase domain (NTD), a middle domain involved in client and co-chaperone interactions, and a C-terminal dimerization domain.^156^ Its activity is regulated by a unique pre-N-terminal extension that acts as a molecular gatekeeper, suppressing premature ATP-driven closure and thereby stabilizing partially open conformations conducive to client engagement.^156,157^ ATP binding triggers local conformational rearrangements, particularly in the NTD lid region, while ATP hydrolysis drives the transition into a catalytically competent closed state needed for productive client folding.

Recent structural and biophysical studies have mapped the conformational ensemble of GRP94 in solution, showing that even in the presence of ATP analogs or ADP, a substantial proportion ( ~60%) of the protein remains in a persistently open “V” state; the nucleotide state instead governs transitions among compact conformers (twist-V and closed-V).^158^ Single-molecule FRET reveals that the transition toward a fully closed state is rate limited and can be specifically accelerated by BiP.^159^ The Pre-N domain is essential in balancing this cycle—its removal increases the ATPase rate but impairs the maturation of authentic clients, confirming its allosteric regulatory role.^157^

In contrast to cytosolic HSP90s, which rely on an extensive network of co-chaperones to regulate their conformational cycles, GRP94 interacts directly with BiP to form a minimal but highly coordinated Hsp70‒Hsp90 system in the ER (Fig. 2).^159^ This collaboration does not require bridging co-chaperones like HOP. Instead, BiP, particularly in its ADP-bound state, binds to GRP94 via a conserved interface, stabilizing a partially closed GRP94 conformation and promoting dimer closure and ATP turnover. Structural and crosslinking studies have identified two functionally distinct BiP–GRP94 complexes: a “preloading” state involving the docking of BiP onto open GRP94 and a “loading” state stabilized by the engagement of a second BiP, which primes GRP94 for client capture.^160^ The ER J-protein DNAJB11 enhances this interaction by accelerating BiP’s ATP hydrolysis and maintaining BiP in a client-bound, ADP-stabilized conformation optimal for GRP94 recruitment.^161^

The chaperoning mechanism of GRP94 is multifaceted. Under mild stress conditions, it functions in tandem with BiP to support ATP-dependent folding. Under a heightened proteostatic burden, however, GRP94 can switch to an ATP hydrolysis–independent “holding” mode, buffering unfolded clients and preventing their aggregation until folding conditions improve.^156,161^ This dual capacity underlines the unique position of GRP94 in the ER folding landscape—bridging the gap between generalist and specialist chaperone functions.

Importantly, the regulation of client affinity by the nucleotide state is a key feature of the mechanism of GRP94.^156,162^ ATP binding reduces the substrate-binding affinity of GRP94, allowing for the release and subsequent maturation of clients, whereas loss of ATP binding results in persistent substrate retention and impaired folding. Recent structural studies have also revealed that BiP facilitates the conformational priming of GRP94 into a client-loading state, stabilizing intermediates that would otherwise be energetically inaccessible and enabling efficient handover of client proteins.^163^ Taken together, these findings emphasize the specialized role of GRP94 in ER proteostasis and its ability to respond dynamically to fluctuations in folding demand.

#### Calnexin and calreticulin: key ER chaperones in glycoprotein folding

Calnexin (CANX) and calreticulin (CALR) are paralogous lectin chaperones that assist in glycoprotein folding within the ER (Fig. 2).^164,165^ Calnexin is an integral membrane protein, whereas calreticulin is soluble; together, they form a membrane‑associated platform that recognizes monoglucosylated N‑linked glycans, a “fold‑me” tag displayed by proteins still going through maturation. Upon binding, chaperones shield hydrophobic patches, prevent premature exit from the ER, and recruit enzymatic partners that accelerate folding.^164^ Both proteins harbor a carbohydrate‑binding globular lectin domain with a proline‑rich, hook‑shaped P‑domain that projects into the lumen and tethers folding catalysts such as ER protein‑disulfide isomerases and peptidyl‑prolyl isomerases.^166^ This structural arrangement concentrates multiple activities on the client and increases the local catalyst concentration, thereby increasing the efficiency of glycoprotein maturation.

Entry into the calnexin/calreticulin cycle begins co-translationally, when the multi‑subunit oligosaccharyltransferase (OST) complex attaches a Glc₃Man₉GlcNAc₂ (G3M9) glycan to emerging polypeptides.^167^ Glucosidase I (MOGS) and glucosidase II (GANAB‑PRKCSH) then remove two glucose residues, generating Glc₁Man₉ (G1M9), the high‑affinity ligand for calnexin/calreticulin.^167,168^ While the client is bound, glucosidase II excises the final glucose, allowing release once the protein approaches its native state. If folding remains incomplete, UDP‑glucose:glycoprotein glucosyltransferase 1 (UGGT1) senses the misfolded conformation—often through exposed hydrophobics—reglucosylates the glycan and sends the substrate back for another round. Multiple iterations of this reglucosylation loop can occur until correct folding is achieved.^141,169^

Persistent non‑native glycoproteins are rerouted: slow-acting mannosidases trim Man₉ to Man₇, a signal recognized by the lectins ERLEC1 and OS9 that deliver the substrate to the ERAD machinery. Clients too large or aggregated for ERAD may instead be dispatched via ER‑to‑lysosome‑associated degradation (ERLAD).^170^

Recent work has expanded the portfolio of calnexins beyond lectin interactions. Its transmembrane domain can directly bind nonglycosylated, misfolded membrane proteins, stabilizing them until they engage canonical quality‑control pathways.^171–173^ Moreover, calnexin partners with the ER‑phagy receptor FAM134B to escort refractory clients—such as aggregation‑prone α‑synuclein—into autophagosomes for lysosomal clearance. Loss of calnexin impairs this pathway, whereas FAM134B overexpression reduces the α‑synuclein burden and ER stress.^171–173^ The nutrient‑responsive factor SESTRIN2 further couples ER stress to this degradative branch. Under XBP1‑driven stress, SESTRIN2 inhibits mTORC1‑mediated phosphorylation of TFEB/TFE3, thereby upregulating FAM134B and lysosomal genes and increasing calnexin‑FAM134B‑mediated ER‑phagy.^172^

Through this layered circuitry—lectin‑assisted folding, reglucosylation surveillance, mannosidase‑triggered ERAD, and calnexin‑guided ER‑phagy—the calnexin/calreticulin system provides the ER with a versatile toolkit for shepherding glycoproteins toward productive folding or timely disposal.

#### Protein disulfide isomerases (PDIs)

The PDI family comprises 21 human paralogs that reside predominantly in the ER lumen, where the oxidizing environment favors disulfide‑bond formation (Fig. 2).^174^ All share a modular architecture built from thioredoxin‑like domains: catalytically active modules carry the Cys‑X‑X‑Cys (CXXC) motif that toggles between oxidized and reduced states, whereas inactive modules act as scaffolds or substrate‑binding platforms.^174^ This mix‑and‑match design underpins the family’s functional diversity, allowing individual PDIs to specialize in distinct client sets or quality‑control pathways.

During oxidative folding, an oxidized PDI donates a disulfide to a substrate cysteine, becoming reduced in the process; reduced PDI is then re‑oxidized by ER oxidoreductin 1 (ERO1) or related flavoproteins, closing the catalytic loop.^175^ If mis‑paired bonds arise, the same enzyme—now in its reduced state—can break and reshuffle them until the native pattern is achieved.^176^ By cycling between oxidation and isomerization, PDIs speed up correct disulfide acquisition and prevent nonproductive cross‑links.

In addition to disulfide bond formation, many PDIs double as holdase chaperones that suppress aggregation.^177^ This redox‑independent activity broadens the scope of ER quality control, particularly for domains that fold slowly or remain partially reduced.

PDIs also interact with stress signaling. PDIA6 binds the luminal domains of IRE1α and PERK, attenuating their activity and preventing runaway UPR signaling; its loss prolongs XBP1 splicing and exacerbates RIDD activity.^178–180^ In contrast, ERp18 regulates the third sensor, ATF6, by transiently holding ATF6 in the ER and reducing its disulfides to enable proper COPII packaging and Golgi processing during stress.^181^ Through such sensor‑specific contacts, PDIs fine‑tune the amplitude and duration of each UPR branch, tipping the balance between adaptation and apoptosis.^181^

In summary, ER-localized chaperones are essential for maintaining the integrity of the secretory pathway by ensuring that proteins are properly folded and functional before exiting the ER. Through a variety of mechanisms, ranging from ATP-driven conformational changes, lectin‒glycoprotein interactions, disulfide bond formation, etc., the cooperative action of these versatile proteins ensures that the ER can handle the vast amount of proteins it processes, maintaining cellular homeostasis and protein quality control.

### HSP100/110 family: protein disaggregases

Protein aggregation, the clumping of misfolded proteins, disrupts cellular function and is implicated in disease.^182^ Disaggregases, a specialized subset of molecular chaperones, reverse this process. Protein disaggregase function was first discovered in 1994, where yeast Hsp104, a member of the HSP100 family, was shown to resolubilize heat-inactivated luciferase from insoluble aggregates, highlighting its critical role in maintaining proteostasis.^183^

HSP100 chaperones belong to the AAA+ (ATPases Associated with diverse cellular Activities) ATPase family and form hexameric rings with a central channel.^182^ These chaperones use ATP hydrolysis to thread aggregated polypeptides through the channel, mechanically disentangling and refolding them.^184,185^ This mechanism was confirmed by fusing bacterial ClpB (Caseinolytic peptidase B protein homolog; an HSP100 disaggregase) to ClpP (caseinolytic peptidase proteolytic subunit) peptidase.^186^ Substrates threaded through the channel of ClpB were degraded by ClpP, confirming directional translocation.

HSP100 disaggregases rarely act alone.^184,187^ In yeast and bacteria, they cooperate with HSP70 (e.g., Ssa1/DnaK) and HSP40 co-chaperones (e.g., Sis1/DnaJ). HSP70 targets HSP100 to aggregates via interactions with its middle domain.^182,188^ Intriguingly, metazoans, including humans, lack canonical HSP100 disaggregases.^93,187^ In the cytosol, instead, they rely on an HSP70-centric system: HSP70 (HSPA1A in humans), HSP110 (HSPH1 in humans, a nucleotide exchange factor/NEF), and J-proteins (DNAJA2, DNAJB1 in humans) collaborate to extract and refold aggregated proteins (Fig. 2).^93,187,189^ This machinery uses the HSP70 ATPase cycle, which is enhanced by the NEF activity of HSP110, to generate mechanical force for disaggregation.

In this system, J proteins such as DNAJA2 and DNAJB1 target aggregated substrates and promote the recruitment of multiple HSP70 molecules onto the aggregate surface.^98,189,190^ Dense arrays of HSP70 chaperones assemble cooperatively, and their ATP-driven binding and release cycles generate mechanical forces that progressively extract polypeptides from aggregates. HSP110 enhances this process by catalyzing nucleotide exchange in HSP70, accelerating chaperone turnover and promoting the formation of a thick HSP70 coating on aggregates. Together, these components transform the canonical HSP70 folding mechanism into a powerful, collective disaggregation machine capable of solubilizing even stable protein aggregates.

In the mitochondrial compartment, however, a dedicated HSP100 family disaggregase operates: CLPB (also known as SKD3).^191,192^ Imported via the TOM/TIM pathway and cleaved by PARL to remove its targeting sequence, CLPB matures in the IMS with two AAA⁺ nucleotide-binding domains (NBD1/NBD2) and a unique N-terminal ankyrin-repeat (ANK) domain for client capture.^192^

Under basal conditions, apo-CLPB assembles into a double-heptamer; upon ATP binding and substrate engagement, it remodels into a substrate-stabilized 12‑mer.^191^ Coordinated ATP hydrolysis in NBD1 and NBD2 drives pore‑loop movements that translocate aggregated polypeptides through the central channel at 2 residues per ATP cycle. The ANK domain recognizes exposed hydrophobic motifs on nonnative IMS proteins, recruiting them to the ring entrance for efficient threading.

Recent functional studies further linked the disaggregase activity of CLPB to mitochondrial calcium signaling and dynamics: CLPB loss causes the regulators of the mitochondrial calcium uniporter complex (mtCU)—MICU1 and MICU2—to become insoluble, reducing mitochondrial Ca²⁺ uptake independently of cytosolic calcium levels and the membrane potential.^193^ Moreover, CLPB directly interacts with the fusion mediator OPA1, preventing its aggregation; without CLPB, OPA1 insolubility increases, leading to mitochondrial fragmentation and a shift toward unstable kiss-and-run fusion events.^193^ Together, these findings reveal that CLPB safeguards both mtCU complex integrity and OPA1 processing to coordinate mitochondrial calcium homeostasis and membrane dynamics.

In addition to HSP100 and HSP70, sHSPs also contribute significantly to the cellular defense against protein aggregation.^182,194^ sHSPs, including HSPB1, HSPB5 (αB-crystallin), and HSPB8, act as ATP-independent “holdases” that bind to unfolded or misfolded proteins early during stress, preventing their irreversible aggregation. These chaperones recognize exposed hydrophobic regions on destabilized client proteins and stabilize them in a soluble, refolding-competent state.^195^ Unlike HSP100 and HSP70, sHSPs do not actively refold substrates; instead, they maintain substrates in a non-aggregated form, effectively “buying time” until ATP-dependent chaperone systems (such as HSP70-HSP110) can initiate refolding or degradation.^196^ Structurally, sHSPs assemble into dynamic oligomers whose size and composition adapt to cellular stress, and post-translational modifications such as phosphorylation (e.g., of HSPB1 at Ser15, Ser78, and Ser82) regulate their chaperone activity by altering oligomeric states.

Notably, specific sHSPs have evolved to partner with the HSP70 machinery directly. HSPB8, for example, forms a constitutive complex with the co-chaperone BAG3, which mediates the selective autophagy of misfolded proteins through the CASA (Chaperone-Assisted Selective Autophagy) pathway.^197,198^ In the cytoskeleton and under mechanical strain, sHSPs such as HSPB1, HSPB5, and HSPB8 work together to stabilize unfolding-prone structures like intermediate filaments and Z-disc components, shielding them from aggregation until repair or turnover can occur.^199,200^ Thus, while sHSPs do not perform mechanical disaggregation themselves, they form an essential frontline of proteostasis, buffering against proteotoxic stress and coordinating with ATP-dependent systems to maintain cellular protein homeostasis.

### TCP1 ring complex (TRiC): a eukaryotic folding chamber

Group II chaperonins are located in the eukaryotic cytosol and are also found in archaea. Unlike Group I chaperonins, which require a separate co-chaperonin (such as HSP10 in mitochondria), Group II chaperonins possess an intrinsic lid mechanism that facilitates protein folding without the need for an external co-chaperonin.^129^ This intrinsic lid is formed by helical protrusions that cover the central cavity upon ATP binding, creating an encapsulated environment for substrate proteins to fold correctly. In eukaryotes, the primary Group II chaperonin is CCT, also known as TRiC.^201^ This complex is composed of two identical stacked rings, each containing eight similar, but nonidentical, subunits named CCT1 through CCT8 (Fig. 2).^202^ Each subunit contributes to the formation of the central cavity and plays a role in substrate recognition and folding. The sequence divergence among the subunits allows TRiC to interact with a broader range of substrate proteins than the more homogeneous Group I chaperonins do.^202^

TRiC assists in the folding of approximately 10% of the eukaryotic proteome, targeting a select set of substrates that are often essential for cellular function.^201,203^ Notably, TRiC is central for the proper folding of cytoskeletal components such as actin and tubulin, as well as cell cycle regulators, including cyclin E, Cdc20, and the von Hippel–Lindau tumor suppressor (VHL).^201^ Many of these substrates are characterized by WD40 repeats, which form propeller-like domains that struggle to fold without assistance.^204^ Additionally, TRiC can fold substrates exceeding 100 kDa in size, which are too large for classical prokaryotic or eukaryotic chaperones to fold effectively.^202,205^

One important feature of TRiC is its ability to bind co-translationally to nascent polypeptide chains as they emerge from the ribosome^202^ (Fig. 2). This early engagement helps prevent aggregation and misfolding from the beginning of protein synthesis. While TRiC can independently facilitate the folding of its substrate proteins, it often collaborates with other chaperones to increase folding efficiency and prevent aggregation.^206,207^ For example, prefoldin, a hexameric chaperone complex, binds to nascent polypeptide chains, stabilizing them in an unfolded state and delivering them to TRiC for proper folding. Despite differences in structure and encapsulation mechanisms, the ATP-driven reaction cycles of both Group I and Group II chaperonins are fundamentally similar. Both utilize ATP binding and hydrolysis to induce the conformational changes necessary for substrate encapsulation and release.^202^ The equatorial domains of the chaperonins, which are responsible for ATP binding, are highly conserved across the two groups, highlighting a shared mechanism of energy transduction driving protein folding.^202,208^

The sequence divergence among the TRiC subunits has expanded the range of possible substrate motifs recognized by the complex.^202^ Unlike Group I chaperonins, which interact primarily with hydrophobic regions on unfolded proteins, TRiC subunits can recognize a variety of structural features. This diversity enables TRiC to assist in the folding of complex and multidomain proteins that are critical for eukaryotic cellular functions.

## The folding continuum: accounting for flexibility and disorder in protein conformations

A discussion of protein folding inevitably refers to the “proper folding” and “native conformation” of proteins; this may lead to the misconception that proteins are limited to one distinct fold permissive of normal function, with deviations representing misfolding and dysfunction. Anfinsen’s thermodynamic hypothesis is frequently simplified to indicate that the amino acid sequence of a protein determines its conformation; if correct, there should not be variations in a protein’s fold. However, Anfinsen’s hypothesis also includes other factors that determine conformation, such as the minimization of Gibbs free energy and “the totality of interatomic interactions […] in a given environment”.^209^ This suggests greater complexity than the simplified version and better mirrors the observed properties of proteins.

It is now well established that proteins differentially populate order and flexibility continua rather than adhering to a folded/unfolded dichotomy.^210^ Instead of referring to a protein’s native conformation, it may be more appropriate to refer to its conformational space, which can be narrow or broad. For example, we currently recognize several classes of proteins that reversibly or irreversibly switch between multiple functional folds or that contain regions that never attain a fixed conformation in isolation. Importantly, the conformational space of such proteins is still sequence-specific (as opposed to random or governed exclusively by other factors).^211^ Below, we discuss protein fold-switching and disorder and introduce different classes of proteins that exhibit fold multiplicity. The clinical importance of these protein classes is revisited later in the article.

Protein dynamics and disorder can be examined through a three-part taxonomy: monomorphic, metamorphic, and disordered proteins. Monomorphic and metamorphic proteins are also referred to as single-fold and fold-switching, respectively, and disordered proteins are best known under the name IDPs or as proteins with intrinsically disordered regions (IDRs), depending on the extent of the disorder. Whereas monomorphic proteins are characterized by a single well-defined conformation, metamorphic proteins may reversibly or irreversibly shift between two or more states.^212^ While metamorphic proteins exhibit distinct conformations that they stably adhere to over time, disordered proteins have no well-defined native conformations, but dynamically sample any intermediate folds between the extremes that are permitted by their sequence and the environment.^212^

There are nuances to these general statements. For example, monomorphic and metamorphic proteins, although generally stable, can still contain shorter flexible and dynamic regions, typically in extruding loops or in termini. They may also undergo more extensive conformational changes, such as with allosteric proteins, or exhibit larger-scale motion of folds relative to each other. For example, the nucleotide-free and nucleotide-bound structures of the bacterial ABC transporter MsbA (orthologous to human MDR1) are geometrically distinct owing to the pivoting and tilting of secondary structures or larger domains; nonetheless, they preserve the individual folds of these structures.^213^ Although this example may appear as fold-switching, the lack of more fundamental rearrangements is consistent with a monomorphic protein. In contrast, the metamorphic chemokine XCL1 can interconvert between an α + β-fold and an all-β-fold, representing a more radical conformational change.^212^ Another distinction is that IDPs, while essentially unfolded in isolation, can undergo induced folding upon certain interactions or environmental changes.^214^ An example is α-synuclein, which can form α-helical multimers upon membrane interaction or stack in β-sheets to form cytosolic aggregates.^215^

It is estimated that 0.5–4% of known protein structures may undergo fold-switching^216^ and that 20–33% of eukaryotic proteins contain IDRs that are at least 30 residues long.^214^ The DisProt database currently includes 1,270 human IDPs, of which 56 are annotated as 100% disordered.^217^ IDRs contribute various properties to proteins that are integral to their function, most notably increasing their number of potential binding partners.^218^ Many transcription factors contain IDRs that facilitate their regulation by a range of transcriptional coregulators or activators/inhibitors. A classic example is p53, which contains IDRs at both its N- and C-termini. The disorder at these regions allows interactions with many other proteins and contributes to the size of its nearly 1,000-protein interactome, each interaction with potentially differential effects on p53 conformation.^214,219^

IDPs also have important functions as scaffolding proteins, where the multiplicity of regulatory mechanisms inherited from the IDR has important implications for the efficiency and coordination of intracellular signaling events.^218^ In addition, a general feature of proteinaceous membrane-less organelles (PMLOs), such as Cajal bodies, nuclear speckles, and stress bodies, appears to be their large-scale inclusion of IDPs, which may contain properties favorable for PMLO biogenesis, including intrinsic flexibility and low-affinity interactions.^220^ Moreover, the prominent role of IDPs such as α-synuclein, tau, and amyloid-β in neurodegenerative diseases featuring protein aggregation, as well as other IDPs implicated in different diseases, may suggest their general clinical relevance^214^ (see below in Section “Causes and consequences of protein misfolding”).

## Causes and consequences of protein misfolding

Protein folding is essential for maintaining a functional proteome and ensuring proper and healthy cellular function.^221^ Failures in protein folding can have various consequences, including loss-of-function or toxic gain-of-function effects on individual proteins, the accumulation or aggregation of misfolded proteins, and the induction of downstream stress pathways. All of these factors may have systemic effects on cellular health, as well as on tissue or organ function, if larger cell populations are affected. This section will examine the factors that can interfere with proper protein folding and the possible ramifications of protein misfolding, including the cellular stress responses that are activated to mitigate folding errors.

### Multiple errors, dysfunctions, or stresses can cause protein misfolding

**Fig. 3:**
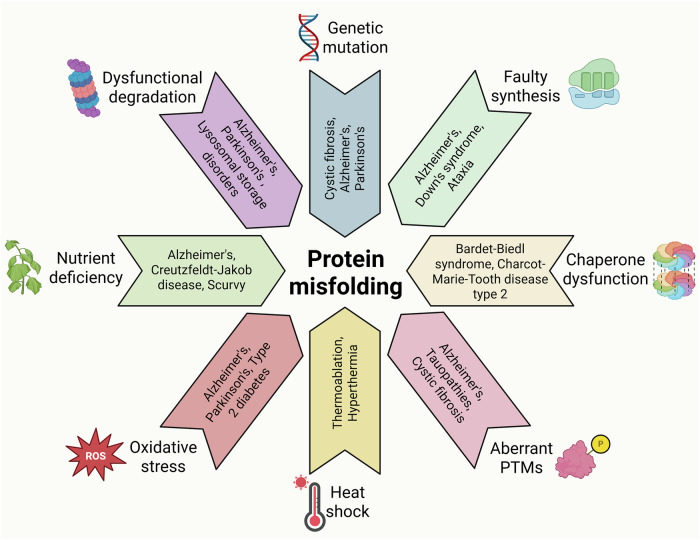
Causes and consequences of protein misfolding. Protein misfolding arises from various stressors, including genetic mutations, faulty synthesis, chaperone dysfunction, aberrant PTMs, heat shock, oxidative stress, nutrient deficiency, and dysfunctional degradation. These factors contribute to the pathogenesis of several neurodegenerative disorders (e.g., Alzheimer’s, Parkinson’s), metabolic diseases (e.g., type 2 diabetes), and other proteinopathies

The important factors that contribute to protein folding include its genetically encoded primary sequence, weak interactions both within the protein and between the protein and its solvent, and the molecular chaperones that aid in the folding process. Here, we outline how different disruptions to these factors may cause protein misfolding and their relevance in different diseases (Fig. 3). We also consider examples of how dysfunctional protein degradation may tip the balance toward an accumulation of misfolded protein species and evaluate the links between aging and protein misfolding. Finally, we consider some of the experimental techniques used to disrupt protein folding pathways in the study of general cellular responses to misfolding.

#### Errors in the protein synthesis pathway

Following the central dogma of molecular biology, every protein is encoded by a gene via its mRNA transcript. Neither the maintenance or replication of the genome nor the transcription and translation that leads to the synthesis of a protein product are mechanisms with perfect fidelity; thus, errors that can occur in any of these steps may result in protein misfolding.^222^

The classical example of a DNA mutation-induced misfolding disease is cystic fibrosis (CF). CF is a rare autosomal recessive disease that most prominently manifests through an accumulation of mucus in the lungs, with subsequent difficulties in breathing, and chronic pulmonary bacterial infections, in addition to effects on the GI tract, pancreas, and reproductive system.^223^ CF is caused by mutations in the cystic fibrosis transmembrane conductance regulator (*CFTR*) gene, a chloride channel with ~5,000 characterized mutations in the NCBI ClinVar database, of which ~1,600 are annotated as pathogenic or likely pathogenic.^224^ On the basis of their effects on the CFTR protein, these mutations are classified into six categories (classes I–VI), of which class II includes mutations that cause CFTR misfolding.^225^ This class includes the F508del mutation, which is the most frequent variant detected in CF and gives rise to a CFTR protein that exhibits defects in channel gating properties,^226^ as well as increased retention in the ER followed by degradation by the ubiquitin–proteasome system (UPS).^227^

In contrast to DNA mutations, transcriptional and translational errors are neither permanent nor typically as penetrant for protein folding. Nevertheless, investigations into the inverse correlation between the expression level and evolutionary rate of a protein indicate that eukaryotic cells are subject to selective pressures that favor the proper folding of proteins.^228^ There is evidence that transcriptional errors sometimes underlie the generation of toxic protein products, e.g., amyloid β precursor protein (APP) and ubiquitin B (UBB), in AD and Down syndrome, respectively, and that these errors occur more frequently in aged cells.^229,230^ In these cases, dinucleotide deletions of GA or GU near GAGAG motifs lead to +1 frameshifted mRNAs translated into C-terminally altered proteins that accumulate in amyloid plaques and neurofibrillary tangles.^230^ It has also been hypothesized that +1 frameshifted UBB—through impaired function of the UPS—may contribute to impaired clearance of aggregating tau oligomers and impair the general contributions of the UPS to proteostasis in neurons.^230^

At the transcript level, aberrant splicing is another cause of pathological protein products. In AD patients, decreased expression of the splicing regulator RBFOX1 has been observed, leading to upregulation of a specific isoform of APP, resulting in increased generation of the highly aggregation-prone amyloid-β peptide Aβ42.^231^ Additionally, approximately one-third of the pathological mutations observed in the gene encoding tau (MAPT) have been reported to affect tau splicing and the ratio of tau isoforms containing three or four microtubule-binding domains (3R-tau to 4R-tau ratio)—a ratio whose imbalance is linked to impaired microtubule dynamics in tauopathies such as AD.^231^

At the translational level, mutations that impair proofreading by alanyl-tRNA synthetase (AARS1), leading to mischarged tRNAs and amino acid misincorporation during translation, can lead to misfolded protein aggregation, ER stress, and Purkinje cell death in the pathogenesis of ataxia.^232^ Estimates have suggested that ~15% of all proteins in a human cell contain at least one misincorporated amino acid,^233^ with possible implications for protein folding. Recent research suggests that some hundred human proteins may exhibit frequent, or even preferential, substitutions of their genetically encoded amino acids at specific positions.^234^ Among these proteins, some are associated with neurodegenerative diseases, such as APOE in AD and TDP-43 or FUS in ALS.^234^ It remains to be explored whether these substitutions contribute to the etiology of folding-related diseases or whether they have other physiological functions that are not currently understood. In summary, imperfect or impaired fidelity in DNA replication or maintenance, transcription, or translation has the potential to cause protein misfolding linked to disease development.

#### Impairment of protein folding mechanisms: chaperonopathies

In Section “Molecular chaperones: Facilitators of protein folding dynamics”, we introduced molecular chaperones, enzymes that can regulate protein folding by interacting with and/or modifying unfolded proteins. Chaperones also fulfill other functions, including scaffolding activity between proteins or organelles, contributions to protein translocation across membranes, and the regulation of signaling pathways. Consequently, chaperone dysfunction can lead to many different pathologies, commonly termed chaperonopathies. The term has been used to describe not only specific diseases but also general states associated with chaperone insufficiency, e.g., aging.^235^ For the purpose of this review, genes annotated as molecular chaperones or co-chaperones in the HUGO Gene Nomenclature Committee (HGNC) database were compared with a list of gene‒disease relationships in the NCBI ClinVar database.^224^ Among the 179 genes annotated as molecular chaperones or co-chaperones in the HGNC database, 48 were associated with a total of 87 unique diseases in the ClinVar database. Among these diseases, some are directly related to dysfunctions in classical chaperone activity (protein folding), whereas others stem from events unrelated to protein folding (e.g., from signaling pathway dysregulation) or from an undetermined etiological mechanism. Below, we present some examples from the first category.

Crystallin alpha B (CRYAB) is an sHSP that contributes to the folding of both cytosolic and transmembrane proteins; in addition, it exhibits stress-dependent activation and protective effects against various amyloidoses.^236,237^ Mutant CRYAB is implicated in the development of an autosomal dominant form of cataract (type 16; CTRCT16) as well as multiple types of myopathy.^224,237^ Crystallins of the alpha, beta, and gamma classes (CRYA/-B/-G, of which only the CRYA proteins are sHSPs^237^) are the major protein constituents of the vertebrate lens, and the CRYA class alone has been reported to make up ~30% of the water-soluble proteins of lens fibers.^238^ Consequently, the structural and functional integrity of crystallins is important in maintaining lens function and vision. Among cataract-associated CRYAB mutations, the D140N variant results in an altered tertiary structure, increased surface hydrophobicity, and increased heat-induced aggregation, with a dominant negative effect on its wild-type counterpart.^239^ In addition, while wild-type CRYAB exhibited intact chaperone activity by inhibiting dithiothreitol (DTT)-induced aggregation of insulin, the D140N variant promoted insulin aggregation at room temperature.^239^ This finding suggests that both the aggregation of CRYAB itself and the negative effects of mutant CRYAB on the folding of other proteins (including wild-type crystallins) could contribute to the etiology of CTRCT16. CRYAB variants are also linked to diseases of muscle tissue, such as desmin-related myopathy (DRM; D109G, R120G) and dilated cardiomyopathy (DCM; D109G, R120G, R123W, G154S, R157H).^237^ An in vivo study on cardiomyocyte-specific expression of the R120G variant further demonstrated how it may affect the brain in the context of ischemia‒reperfusion stress (mimicking ischemic stroke). R120G-mutant CRYAB undergoes amyloidogenic misfolding in the heart, disseminates to the brain via exosomal transport, and leads to increased neuroinflammation, increased infarct volume, and impaired functional recovery.^240^ The protein has also attracted interest in neurodegenerative conditions, e.g., owing to its elevated concentration in AD brains, accumulation in amyloid plaques and a range of other aggregates or inclusion bodies, and potentially neurotoxic contributions in complex with the Aβ peptide.^241,242^

Other diseases related to chaperone dysfunction include a cystic fibrosis-like disease with respiratory, gastrointestinal, hepatic, and cardiovascular symptoms that is linked to loss-of-function mutations in AGR2, encoding a PDI.^243^ Mechanistically, AGR2 is involved in the production of the intestinal mucin MUC2, as well as the airway mucins MUC5AC and MUC5B^244,245^—these mucins are cysteine-rich proteins that depend on many disulfide bonds to attain their native conformation. Although this etiological mechanism has not been validated in patients, the symptoms of these AGR2-associated diseases are thus linked to the loss of PDI activity and the resulting mucin insufficiency, with presumptive effects on mucous barrier integrity in vital organs. In addition, four different chaperones are associated with the development of Charcot-Marie-Tooth (CMT) disease type 2 and distal hereditary motor neuropathy (dHMN): HSPB1, HSPB3, HSPB8, and DNAJB2. Approximately 30 different HSPB1 variants have been associated with CMT type 2 or dHMN—S135F being the most frequent and associated with both.^246^ HSPB1 mutations have diverse effects, many of which affect microtubule binding and axonal transport in neurons, including the disruption of aggresome formation and the subsequent autophagic clearance of misfolded proteins by the S135F variant.^246–248^ The effects of many HSPB1 variants on chaperone activity remain poorly understood, in part because current in vitro assays—such as aggregation suppression tests—typically assess function against a single model substrate. However, HSPB1 exhibits substrate-specific activity, and different variants may differentially affect the folding of distinct clients, complicating interpretation.^249^ This limits our ability to draw generalizable conclusions about variant pathogenicity. Therefore, more comprehensive and physiologically relevant approaches are needed to elucidate how specific HSPB1 mutations contribute to the CMT type 2 and dHMN phenotypes.

#### Errors in co- or post-translational protein modifications

Various protein modifications that occur during or after protein synthesis (co- or post-translational), such as cofactor incorporation, glycosylation, lipidation, and other modifications, affect the folding and conformation of many proteins. In addition, some protein modifications are used by cellular quality control and degradation processes to prevent the accumulation of misfolded proteins. This section describes some of the protein modifications that play a role in protein folding, quality control, stability, and degradation and the effects of their disruption.

##### Phosphorylation

Phosphorylation is among the most common PTMs in the human proteome. It is estimated to affect the conformation, function, stability, localization, or interactions of ~30% of all human proteins and is regulated by ~500 protein kinases and ~200 protein phosphatases.^250^ Given the widespread use of this PTM in maintaining cellular function, aberrant phosphorylation may have multiple disruptive effects, some of which are related to protein folding and aggregation. Although nine of the standard amino acids can be phosphorylated in principle, the phosphorylation of threonine, serine, and tyrosine are the most well studied and are likely the most abundant phosphorylation events in human cells.^251^ Phosphorylation reversibly adds a dianionic (at physiological pH) phosphoryl group to an amino acid side chain, altering its bulk and charge and modifying its bonding capacities (e.g., facilitating hydrogen bonding, salt bridges, or metal ion coordination). Phosphorylation may thus directly induce conformational changes in a protein but also participate indirectly through the regulation of other PTMs, e.g., by tagging Ser/Thr-Pro motifs for proline isomerization.^252^ In addition, phosphorylation may induce folding events in intrinsically disordered proteins, thereby regulating their activity.^253^

Multiple neurodegenerative diseases are associated with abnormal phosphorylation of aggregation-prone proteins or peptides. A prototypical example of this is the hyperphosphorylation of tau in AD and other tauopathies, such as subtypes of frontotemporal dementia (specifically FTDP-17). Tau is an important regulator of microtubule dynamics, and the microtubule-binding properties of tau are directly regulated by its phosphorylation both within and outside of its microtubule-binding domains.^254^ Early autopsy studies revealed that hyperphosphorylated tau is present in virtually all brain tissues from AD patients but is absent in non-AD controls.^255^ Quantitatively, these studies indicated that abnormally phosphorylated tau in AD patients contained three to four times more phosphate groups ( ~ 7–9 mol PO_4_^-^ per mol tau) than did normal tau ( ~ 2–3 mol PO_4_^-^ per mol tau).^255^ These clear trends are validated in more recent studies, where e.g., mass spectrometry has been used to show dramatic increases (approximately two orders of magnitude) in insoluble tau in AD brain tissue, as well as more extensive and frequent phosphorylation of tau in AD tissue than in control samples, in addition to other modifications, such as increased acetylation, ubiquitination, and methylation.^256^

Abnormal tau phosphorylation is also observed in the tauopathies FTD, corticobasal degeneration, and progressive supranuclear palsy, although the tau isoforms and phosphorylation patterns show some variation between these diseases.^257^ Tau hyperphosphorylation promotes its dissociation from microtubules, and while the prevailing view is that tau hyperphosphorylation promotes its aggregation, the direction of causality between tau phosphorylation and aggregation in disease, or the relative contributions of all tau PTMs, is not clear.^257–259^ Combined with the observation that tau aggregation correlates with AD progression, it is assumed that tau aggregation in combination with Aβ pathology and other contextual features contributes to AD development.^257,259^ Mechanistically, the identification of a relatively large number of protein kinases ( >20)^260^ and phosphatases^261^ that recognize tau as a substrate allows for multiple ways in which tau phosphorylation may be dysregulated. Notable tau kinases include GSK3α and -β—which act on the tau residues that are hyperphosphorylated in AD, and whose knockdown reduces tau phosphorylation and aggregation in a mouse model of AD—as well as CDK5, protein kinase A, MARK1/-2, casein kinase 2, and others.^257,259,260^ Multiple mechanisms may act in concert to tip the balance in favor of tau hyperphosphorylation, such as oxidative stress, calcium dyshomeostasis, age-related alterations, or even Aβ-mediated modulation of cell surface receptors at synapses^262^; more research is needed to assess this phenomenon. Other protein aggregates in different neurodegenerative diseases are also associated with abnormal phosphorylation; for example, hyperphosphorylation has been described for TDP-43 aggregates in amyotrophic lateral sclerosis (ALS)^263^ and α-synuclein (SNCA) in Lewy bodies in PD and dementia with Lewy bodies.^264^ Hypophosphorylation of huntingtin (HTT) at T3 has been associated with Huntington’s disease (HD), and HTT S13 phosphorylation has been shown to inhibit HTT aggregation and promote its autophagic clearance.^265^

A pressing question for all of these observations is whether phosphorylation regulates the aggregation of these proteins, or vice versa, or a combination of the two. The phosphorylation of different residues may also have different effects (i.e., promoting vs. inhibiting aggregation); furthermore, it is possible that the hyperphosphorylation patterns observed in AD may not have effects similar to those of regular tau phosphorylation in regulating tau microtubule binding. In AD, tau phosphorylation has been reported to precede the formation of tau neurofibrillary tangles (NFTs), supporting a direction of causality from phosphorylation to aggregation^266^; however, it is still possible that intermediate oligomeric forms of tau (preceding NFT formation) may promote some or all of the hyperphosphorylation. A study evaluating the microtubule-binding properties and polymerization kinetics of 16 different tau phosphomimetic mutants revealed that most mutants decreased the binding affinity of tau for tubulin, whereas their effects on tau polymerization, as well as the lag time and rate of polymerization, were more variable.^267^ These in vitro studies may not perfectly recapitulate the effects of specific phosphorylation events in vivo but illustrate that there is no universal rule for all possible tau phosphorylation patterns and that tau polymerization or aggregation is not a necessary consequence of tau phosphorylation. Taken together, the phosphorylation of abnormal protein aggregates is a common trend in many neurodegenerative diseases, but protein- and disease-specific phosphorylation codes are currently not understood. A more detailed understanding of the causes and consequences of the phosphorylation of pathogenic misfolded proteins could have therapeutic implications depending on the respective proteins’ etiological contributions and druggability.

In addition to its direct effects on conformation, phosphorylation is also an important regulator of chaperone ATPase activity, as are their interactions with co-chaperones.^268^ Specific C-terminal phosphorylations of the HSP70 and HSP90 family members HSPA1A and HSP90AA1 at T636 and T725/S726, respectively, which lead to their dissociation from the E3 ubiquitin ligase CHIP in favor of interaction with the co-chaperone HOP, were introduced previously.^104^ The CHIP/HOP balance is relevant for neurodegenerative diseases, as STUB1 is an important contributor to tau degradation; moreover, tau phosphorylation at S416 is associated with decreased recognition by CHIP and increased tau aggregation.^269^ Another previously discussed chaperone—CRYAB—is also phosphorylated under stress, resulting in a shift in its subcellular localization, purportedly increasing its activity.^237^

##### Glycosylation

Protein glycosylation consists of the covalent linkage of carbohydrate oligomers or polymers (glycans) to proteins. The monosaccharide makeup, branching pattern, length, and chemical modifications of the glycans, as well as their attachment points on the target proteins, lead to a vast diversity of glycoconjugates that may confer various properties to their associated proteins, regulating, e.g., their conformation, localization, interactions, stability, solubility, etc.^270^ It is estimated that up to 4% of the human genome encodes proteins involved in glycosylation, such as glycosyl- and oligosaccharyltransferases, glucosidases, epimerases, and nucleotide–sugar transporters.^270^ Owing to the cell type and state-dependent expression and regulation of these enzymes, a cell’s glycoproteome is consequently context-dependent.^270^

Glycosylation occurs primarily in the ER, where the co-translational N-linked glycosylation pathway and post-translational O-linked glycosylation pathways are initiated, and further processing occurs in the Golgi apparatus (as well as the initiation of several smaller glycosylation pathways).^270^ While the cores of N- and O-linked glycans typically remain attached permanently, transient and dynamic O-GlcNAcylation is also carried out in the cytosol and nucleus to regulate processes such as transcription, translation, or cell cycle progression in response to stress- and nutrient-related cues.^270^ Glycans may also be C-linked or C-terminally GPI-anchored (hybrid glycan/lipid modifications), but the focus of this review will be on N- and O-linked glycans and how they contribute to protein conformation, folding, and quality control.

In principle, there are numerous ways in which glycoconjugates may shape and stabilize the native conformation of a protein. Glycans are both bulky and primarily hydrophilic,^271,272^ with the potential to participate in hydrogen bonding and van der Waals interactions with both the polypeptide backbone and side chains. They may sterically restrict certain conformations, contribute with allosteric effects to other regions of the protein, mediate intra- and intermolecular interactions that stabilize tertiary and quaternary conformations, or shield hydrophobic regions to restrict folding errors from the hydrophobic effect, aggregation, and increase a protein’s solubility.^271–273^ Glycans can also influence protein folding through enthalpic and entropic effects on the unfolded or folded state of a protein. Folding is promoted by destabilization of the unfolded state (increased enthalpy or decreased entropy) or stabilization of the folded state (vice versa).^272^ Entropic effects, particularly in the unfolded state, are suggested to be the major contributors, and potential enthalpic effects depend on the hydrophobicity of the specific glycan.^272^

Interestingly, many glycoproteins can function normally when their glycan(s) are removed after folding, despite relying on the glycan for proper folding and exhibiting decreased stability in their folded state without glycans.^273^ For example, the human ribonuclease RNASE1 can be N-glycosylated at three different sites (N34, N76, and N88); N34 glycosylation is the most prevalent one in vivo, as well as being the most evolutionary conserved glycosylation site.^274^ While N-glycosylation (particularly at N34) enhances the thermostability of RNASE1 and resistance to proteolysis, a decrease in its in vitro enzymatic activity was observed for six of its seven glycoforms, of which only N34-monoglycosylated RNASE1 showed comparable activity to the aglycosylated enzyme.^274^

Recombinant human erythropoietin (rhEPO) also has increased activity in vitro when it is aglycosylated, whereas the aglycosylated form has almost no activity in vivo.^275^ rhEPO glycosylation decreases the rate of its metabolism both by the kidneys and liver and thereby greatly improves its half-life in circulation. Moreover, mutation of three of rhEPO’s four glycosylation sites (N36, N83, and S126, but not N24) has been shown to deplete secreted and intracellular soluble rhEPO from baby hamster kidney cells, leaving the only detectable rhEPO to be found in their insoluble fraction, suggesting that these aglycosylated states do not fold properly and are readily degraded.^276^ This example of rhEPO is a reminder that in vivo properties may not adhere to patterns observed in vitro, which has implications for the potential translational impact of recombinant protein therapeutics.

Disrupted glycosylation may also characterize some CFTR variants associated with cystic fibrosis. CFTR is N-glycosylated at N894 and N900, and mutation of these residues can abolish CANX binding to CFTR in the ER; loss of these residues also impairs CFTR maturation and folding efficiency with both CANX-dependent and CANX-independent components.^277^ ΔN900 mutants, in particular, are linked to the development of CF and appear to have substantially decreased stability compared with WT CFTR.^278^

Perhaps the best characterized role of glycans in protein folding is that of N-glycosylation in ER protein folding and quality control, as described in Section “Molecular chaperones: Facilitators of protein folding dynamics”. Experimentally, one of the ways in which ER stress is commonly induced is by tunicamycin, an inhibitor of N-linked glycosylation. In addition to the central role of N-linked glycosylation in ER protein folding and quality control, studies have shown that O-linked glycosylation may also contribute in the case of certain substrates. Two ER-localized soluble fucosyltransferases—POFUT1 and POFUT2—have been reported to O-fucosylate proteins containing EGF repeats and thrombospondin type I repeats, respectively, in a discriminatory manner where they act only on properly folded substrates.^279^ While this O-fucosylation is suggested to promote further folding, O-mannosylation catalyzed by ER-resident protein O-mannosyltransferases has been shown to label misfolding proteins for ERAD in yeast.^279^ However, as this modification is prevalent on many proteins and is essential for the function of some (e.g., E-cadherin [CDH1]), it likely has pleiotropic functions.^280^

In summary, while the folding contributions (or lack thereof) of various glycoconjugates remain uncharacterized for most proteins, there is evidence to support their impact on folding kinetics and protein stability. However, at present, there is not sufficient experimental evidence to unequivocally support or oppose this notion; thus, further research is needed in this area. Faulty or dysregulated glycosylation is implicated in multiple diseases. More than 170 congenital disorders of glycosylation (CDGs) have been identified, with roots in virtually any of the various glycosylation pathways in the cell and with clinical manifestations that range from mild to severe or even embryonic lethal.^281^ On the basis of the available evidence, the extent to which the etiologies of these diseases are linked to specific kinds of disruption, such as aberrant folding of the affected glycoproteins, is unclear. Glycosylation has also been investigated in AD, where a dysregulated glycome and increased glycosylation of tau have been reported, and other proteins that may participate in AD etiology are also described to be under regulation by their glycosylation pattern, such as the activity of the BACE1 protease and possibly the processing of APP.^282^ While there are currently few successful therapies for glycosylation-related diseases, further exploration of the glycobiology of AD and other diseases may help improve our understanding of their etiology, as well as the development of future diagnostic and therapeutic approaches.

##### Cofactor incorporation and metalation

Many proteins incorporate cofactors such as metal ions (Cu^+^, Fe^2+^, Mn^2+^) or organic molecules (vitamin B_12_, NADPH, FMN, etc.) that contribute to their structure and are required for their function. Estimates suggest that more than one-third of the human proteome, and more than 40% of its enzymes, are metalloproteins.^283,284^ Most metalloproteins directly accept their required metal ions into metal-binding sites (e.g., iron‒sulfur proteins), whereas some require that the metal ion is already incorporated into an organic molecule.^283^ For example, iron must be incorporated into protoporphyrin IX by a ferrochelatase to form a heme molecule before it can be utilized by hemoproteins such as hemoglobin and cytochrome *c*. Similarly, molybdenum must be incorporated into molybdopterin to form the molybdenum cofactor (Moco) used by various oxidoreductases, such as xanthine dehydrogenase (XDH).^283^

While some proteins accept their cofactors after folding into their native conformation, others incorporate their cofactors during the folding process and diverge in the folding kinetics, structure, and stability of their apo- and holoprotein forms. For example, it has been reported that the Cys_2_His_2_ zinc finger motif—which, e.g., mediates DNA binding for numerous human transcriptional regulators—fails to adapt its functional ββα fold in the absence of zinc.^284^ Research has also shown that depletion of riboflavin (vitamin B_2_), which is essential for the formation of the FMN and FAD cofactors, leads to a general decrease in the levels of flavoproteins due to increased degradation of apoflavoproteins.^285^ One of these flavoproteins, NQO1, is directed toward degradation by STUB1-mediated polyubiquitination upon riboflavin depletion; this degradation could be inhibited by truncation of NQO1’s C-terminal tail, which shows signs of unfolding in the absence of riboflavin.^285^ Moreover, when B16 cells were treated with the Aβ42 peptide to seed protein aggregation, total protein aggregation was substantially greater in riboflavin-depleted cells. This may be explained by an increased propensity of flavoproteins to aggregate upon riboflavin depletion, as truncated apo-NQO1 coaggregated less with Aβ42 than did wild-type apo-NQO1.^285^

Other examples of the impact of cofactor incorporation on protein structure include proteins implicated in neurodegenerative diseases. The prion protein (PrP), whose misfolding causes Creutzfeldt–Jakob disease in humans, attains a more rigid and misfolding-resistant conformation upon copper binding.^286^ Frataxin (FXN), whose deficiency (mostly due to genetic GAA repeat expansions in the *FXN* gene, impairing its transcription) is causative for Friedreich’s ataxia (FRDA), and a putative iron chaperone that contributes to the assembly of iron–sulfur proteins.^287^ Consequently, iron dyshomeostasis and deficient activity of iron‒sulfur enzymes are hallmarks of FRDA and may be major etiological components, perhaps together with increased ROS levels caused by mitochondrial iron accumulation.^287^ In addition, FXN missense mutations that are observed in some cases of FRDA affect FXN’s iron binding capacity and stability; this finding suggests that mutant FXN may not be pathological only because of dysfunctional iron incorporation into other proteins but also because of impaired iron binding of FXN itself, with consequences for its stability and/or metallochaperone activity.^287^

Finally, the demetalation of copper and zinc from SOD1 induces its misfolding and aggregation.^288^ This finding has important implications for ALS, where >100 different SOD1 mutations are linked to a familial form of the disease.^288^ Some of these mutations are associated with abrogating SOD1’s metal-binding sites,^288^ whereas others may inhibit functional interactions between SOD1 and its copper chaperone CCS.^289^ Notably, SOD1 aggregates are metal-deficient in a mouse model of familial ALS (while soluble SOD1 has a relatively high metal content), suggesting that SOD1 cofactor deficiency may have clinical relevance.^288^ In contrast, neuronal accumulation of metal ions and their interaction with disease-associated protein aggregates is also a hypothesized etiological mechanism in multiple neurodegenerative diseases, implying that supplementation may not be the simple solution.^290^ Together, these examples indicate that nutritional deficiencies or insufficient protein cofactor incorporation, e.g., due to loss of function in specific transporters, cofactor chaperones, or the cofactor-binding sites of proteins themselves, may lead to dysproteostasis through effects on protein folding and stability. Alternative upstream causes may include the inhibition of cofactor incorporation, or cofactor displacement, by certain toxins or heavy metals. While heavy metals disrupt protein folding, in most cases, it may not be clear whether this phenomenon is due to effects on protein cofactors or other mechanisms, such as the induction of oxidative stress.^291^

#### Genotoxic or proteotoxic stresses and deficiencies

##### Heat shock

Heat promotes protein denaturation by increasing the rate at which weak noncovalent intramolecular bonds in proteins are broken (e.g., van der Waals interactions and hydrogen bonds). Although van der Waals interactions and hydrogen bonds have bond dissociation energies as low as ~4 and ~23 kJ/mol, respectively, and the average thermal energy for particles in an aqueous solution at 37 °C is ~38.6 kJ/mol, multiple factors (rapid bond recycling, the hydrophobic effect) maintain protein conformation at physiological temperatures.^292^ An interesting question is whether the abnormal temperature increases experienced by humans in cases such as fever, hyperthermia, or even thermoablative/hyperthermic therapies increase the rate of bond dissociation sufficiently to challenge protein folding and proteostasis.

Fever and hyperthermia both refer to elevated basal body temperatures above the normal range of ~36.0 to ~37.5 °C. Previous work has shown that these temperatures have pleiotropic effects on cells, altering membrane properties, metabolism, anabolic processes, and cell morphology.^293^ In vitro studies have shown appreciable cytotoxicity at temperatures ≥41 °C, with more dramatic effects at temperatures ≥42.5 °C, possibly with cell line-dependent variations.^294^ Proteins differ in their thermolability, but evidence from differential scanning calorimetry (DSC) in human and mammalian cell lines suggests that signs of protein denaturation are detectable at 37–38 °C, are significant at 40–41 °C, and rise steeply with increasing temperature.^293^ While the long-term consequences at these temperatures are less clear, they are likely limited by some level of thermotolerance, mediated, e.g., by activation of the HSR. Multiple studies in human and mouse cell lines, as well as in vivo experiments, have shown that febrile temperatures (38–39.5 °C) activate the HSR—as measured by HSF1 trimerization, HSF1 DNA-binding activity, and/or HSP70 family RNA and protein expression—in a dose-dependent manner, with minimal effects from temperatures at the lower end of the range or under brief heat exposure.^295,296^

Fewer studies have examined other stress response pathways at physiologically relevant temperatures, and findings suggest that temperature effects are context dependent. In HeLa cells, all three arms of the UPR were activated after three hours at 43 °C but not at 40 °C.^297^ In AD293 cells, 40 °C upregulated UPR markers (HSPA5, DNAJC3, GADD34, CHOP, and XBP1s), whereas 43 °C repressed them.^298^ In this study, Hepa-1, HepG2, INS1 832/13 cells, and MEFs incubated at 40 °C presented limited and heterogeneous responses to some of the same UPR markers. A common trend was GADD34 induction, which was only accompanied by CHOP upregulation in INS1 832/13 cells, perhaps suggesting divergent regulation of the PERK arm of the UPR.^298^ In contrast, HSPA1A was consistently upregulated upon mild heat shock in these cell lines, suggesting a more predictable induction of the HSR.^298^

Comparisons of the HSR and UPR in MEFs and U2OS cells revealed differences in temperature sensitivity between both responses and cell lines.^299^ HSF1 was upregulated at 39–44 °C in U2OS cells but not in MEFs. HSPA1A was strongly induced in both cell lines, whereas HSPB1 was slightly upregulated in U2OS cells and was primarily upregulated in MEFs at 42–43 °C. Mild heat shock had little effect on the UPR sensors: IRE1α and ATF6 were not transcriptionally induced, and PERK increased slightly at 40–41 °C in U2OS cells. The UPR effectors showed stronger responses in U2OS cells—XBP1s and CHOP increased at 40–44 °C and 39–42 °C, respectively—although XBP1s was only slightly upregulated in MEFs at 43–44 °C. Interestingly, ATF4 mRNA levels were downregulated in MEFs at 39–42 °C but remained unchanged in U2OS cells. While the evidence is limited, HSR activation downstream of mild heat shock appears to be less context-dependent than UPR activation. This may be due to differences between the ER and cytosolic proteomes, where those composed of transmembrane and secreted proteins in the ER may be more heat tolerant.^299^

##### Inflammation and oxidative stress

Inflammation is a response to tissue injury or infection that normally promotes the elimination of foreign objects and self-repair.^300^ An important component of inflammation is the generation of oxidative stress, which in turn may cause more proinflammatory signaling.^300^ Key mediators of oxidative stress include reactive oxygen species (ROS) and reactive nitrogen species (RNS). ROS and RNS are not only generated in response to proinflammatory signaling but are also natural byproducts of multiple constitutively occurring cellular processes and, in some cases, are also utilized as secondary messengers. The ROS constitute molecules such as the hydroxyl radical (^•^OH) generated from H_2_O_2_ by the Fenton and Haber-Weiss reactions.^301,302^ A second major ROS is the superoxide anion radical (O_2_^•–^), which is produced by the reaction of O_2_ with electron carriers of complexes I and III in the mitochondrial electron transport chain or by NADHP oxidases.^303^ A third key ROS is hydrogen peroxide (H_2_O_2_), which is produced by superoxide dismutase (SOD)-mediated conversion of O_2_^•–^ and from ERO1A-mediated oxidation of protein disulfide isomerases during ER disulfide bond formation.^304^ Among these three types of ROS, H_2_O_2_ is a weaker oxidant and is associated with more selectively applied oxidative damage, whereas the strong oxidant HO^•^ may cause extensive damage.^301,305^ Moreover, different oxidants, particularly the less reactive ones, exhibit target selectivity, differing, e.g., in the amino acid side chains, they typically react with.^306^ The RNS include nitric oxide (NO^•^) and peroxynitrite anion (ONOO^–^) — the latter generated in a reaction between O_2_^•–^ and NO^•^^302^. NO^•^ functions as a neurotransmitter or vasodilator when it is produced by NOS1 (nNOS) or NOS3 (eNOS), respectively, whereas NOS2 drives NO^•^ production downstream of cytokine or PAMP signaling.^302^ In addition to its oxidant effects, NO^•^ may also exacerbate oxidative stress through the inhibition of glutamate-cysteine ligase (GCLC), leading to decreased synthesis of the abundant and important endogenous antioxidant glutathione (GSH).^307^

Oxidative stress may disrupt proteostasis through multiple mechanisms. First, ROS and RNS are mutagenic substances that may react with DNA to produce different DNA lesions, such as 8-oxoG and 8-nitroG, which may cause G to T transversions or single-strand breaks by damaging the 2-deoxyribose backbone.^308^ If unrepaired, these lesions may cause genetic entrenchment of aberrantly folded and/or functioning protein sequences. ROS and RNS may damage not only nucleic acids but also other macromolecules, including lipids, glycans, and proteins.^305^ Proteins can be oxidized both at their backbone and at their sidechains, with possible consequences including fragmentation, crosslinking, cystine (or mixed disulfide dimer) formation, decarboxylation, deamination, tryptophane loss, and adduct formation, with implications for protein conformation, polarity, and interactions.^305^ Moreover, sidechain radicals generated by oxidative damage can in turn oxidize other macromolecules, including other proteins, lipids, and DNA, potentially propagating oxidative damage.^305^ Most side chain oxidation products are hydrophilic and may therefore be more disruptive to protein conformation when they occur at residues with higher side chain hydrophobicity.^305^ Since these residues are typically buried within the protein, however, they are simultaneously less readily oxidized.^305^ Common products from the oxidation of aliphatic side chains are alcohol, carbonyl, and hydroperoxide groups, whereas aromatic ring oxidation may introduce hydroxy adducts, electrophilic substituents (e.g., nitro-, chloro-, bromo-), ring crosslinking, or ring opening (e.g., tryptophan → kynurenine).^306^ This notwithstanding, the oxidation of hydrophilic side chains can also lead to substantial impacts on the bulk, hydrophilicity, charge, and bonding propensities, such as aspartate or glutamate decarboxylation (removing a negative charge) or arginine oxidation to glutamic semialdehyde (losing the positively charged guanidinium group).^306^ Importantly, in addition to the reduction of cystines and some other examples, proteins suffering oxidative damage are rarely repaired but are frequently subjected to proteolysis (albeit depending on the modification).^305^

Inflammation and oxidative stress have been identified as contributing causes of multiple groups of diseases or degenerative processes, including obesity and metabolic conditions, cancer, neurodegenerative diseases, cardiovascular diseases, and aging.^309,310^ A substantial body of research associates oxidative stress with neurodegenerative diseases. For example, aging brains exhibit metal dyshomeostasis, including iron and copper accumulation, of which copper has been linked to promoting Aβ and tau aggregation in AD; iron and copper may co-aggregate with Aβ, and these aggregates have oxidant activity, generating H_2_O_2_.^290,311^ Age- and AD-related iron or copper accumulation may increase the rate of Fenton and Haber–Weiss reactions, which could contribute to the elevated oxidative stress markers observed in the aged and AD brains.^311^ Similarly, a bidirectional link between zinc and oxidative stress, where oxidative stress can promote zinc accumulation and where zinc accumulation exerts prooxidant effects, has also been proposed for neurodegenerative diseases.^312^ A pertinent question here is whether protein aggregation may introduce oxidative stress in some disease contexts. There is evidence that different species of protein aggregates associated with neurodegenerative diseases may act to promote ROS generation.^290^ In addition to the oxidant activity of Aβ/copper aggregates, PD-associated α-synuclein oligomers are also oxidants and have been shown to increase cytosolic ROS production, as well as cause mitochondrial lipid and protein oxidation, leading to mitochondrial dysfunction.^313^

##### Micro- and macronutrient deficiencies

The possible consequences of deficits or dysfunction in cofactor incorporation that were discussed previously relate to the larger-spanning topic of how micro- or macronutrient deficiencies may perturb protein folding and proteostasis. A recent estimate suggested that 99.3% of the world’s population may suffer from inadequate micronutrient intake.^314^ In addition, nutrient deficiencies may also stem from diseases or genetic predispositions, adding to their prevalence and impact. For example, vitamin E, vitamin C, and selenium deficiencies are estimated to affect 67%, 53%, and 38% of the global population, respectively.^314^ These three micronutrients are important antioxidants: selenium is a required cofactor for multiple antioxidant enzymes (including glutathione peroxidases and thioredoxin reductases), whereas vitamins C and E interact directly with ROS to reduce them, with vitamin C also contributing to the recycling of oxidized antioxidants (e.g., vitamin E).^315^

With this background in mind and the posited effects of oxidative stress on protein folding as outlined in the previous section, the deficiencies mentioned above have clear implications for proteostasis. For example, severe vitamin C deficiency causes scurvy, which consists of connective tissue degeneration and is lethal when left untreated.^292^ This is due to the essential role of vitamin C as a cofactor for the lysyl and prolyl hydroxylases that are involved in collagen biosynthesis and the integral role of hydroxylysine and hydroxyproline modifications in collagen conformation and crosslinking.^292^ Without vitamin C, the lysines and prolines in collagen are not sufficiently hydroxylated, and the collagen monomers fail to fold and crosslink properly, leading to connective tissue pathology.^292^

Another important antioxidant is the tripeptide glutathione (γ-glutamyl-cysteinyl-glycine [GSH]), whose biosynthesis depends on amino acid precursor availability and may be impaired by a protein-deficient diet (or certain diseases), specifically cysteine (and methionine), glutamate (and glutamine), or glycine deficiencies.^307^ Amino acid deficiency may also disrupt proteostasis in a more direct manner. One example is tryptophan deficiency, which, e.g., is observed downstream of the upregulation of IDO1 (a tryptophan-catabolizing enzyme) in certain tumors.^316^ Tryptophan deficiency leads to the misincorporation of phenylalanine at tryptophan codons by WARS1 (so-called W >F substitutions) and was observed to cause proteome-wide W >F enrichment, with suspected implications for protein folding and function (in many cases, due to the occurrence of highly conserved residues).^316^ Notably, T-cell-mediated interferon-γ (IFNG) secretion (which may be generalized to inflammation) appears to be an upstream regulator of IDO1 expression.^316^ These findings have therapeutic implications, as tryptophan-deficient tumors also display a greater number of neoantigens, promoting the immune recognition of tumor cells.^316^ Tryptophan is also used in the biosynthesis of niacin (vitamin B3; which otherwise can be obtained through the diet), a precursor for nicotinamide adenine dinucleotide (NAD), which is an essential cofactor for several enzymes.^317^ Among these enzymes is the sirtuin family of NAD-dependent protein deacetylases,^317^ whose member SIRT1 supports the activity of HSF1 (the master regulator of the heat shock response), as well as other stress-inducible transcriptional regulators.^318^ The disruptive effects of amino acid deficiency on proteostasis are also apparent from the induction of the integrated stress response (ISR) through GCN2 (EIF2AK4) under such conditions, leading to a global attenuation of protein synthesis and selective expression of certain genes, such as amino acid transporters and certain biosynthetic enzymes.^319^

#### Dysfunctions in protein quality control and degradation machinery

Protein degradation is integral for removing both misfolded, damaged or outdated proteins from the cell and is continuously carried out via different pathways. Two of the main pathways involved in this process are the UPS and lysosomal proteolysis downstream, of e.g., autophagy or endocytosis.^320^ Related to the UPS is the ERAD pathway, which specifically recognizes misfolded proteins within the ER and translocates them for UPS-mediated degradation in the cytosol^170^; similarly, protein degradation processes are associated with other intracellular compartments, such as the nucleus^321^ and mitochondria.^322^ The major routes for protein degradation are discussed in more detail below, primarily in the context of stress responses, but it is important to note that they are also continuously active in unstressed cells.

The UPS mediates the degradation of ~15% of nascent polypeptides (co-translational ubiquitination), even in unperturbed cells; this degradation significantly increases when cells are subjected to signals that promote protein misfolding or translational stalling.^323^ Accordingly, UPS dysfunction has been implicated in multiple diseases. In AD, both Αβ and Tau, the major pathological aggregating proteins, are UPS substrates. In addition, as mentioned in Section “Errors in the protein synthesis pathway”, the age- and AD-associated increases in +1-frameshifted ubiquitin B can block polyubiquitination and lead to an impairment in UPS function.^230^ Intriguingly, it has been suggested that Aβ accumulation in AD may stem from decreased clearance rather than increased generation.^324^ In PD, some familial variants are linked to mutations in *PRKN*, which encodes an E3 ubiquitin ligase, and PD-associated α-synuclein accumulation has also been shown to inhibit UPS activity (for a detailed discussion, see Lim & Tan (2007)^325^). Moreover, a few additional genes encoding UPS components have been linked to other neurodegenerative disorders (including UBQLN2 and VCP2 in ALS) and other pathological protein aggregates (e.g., Tau, TDP-43, and polyQ proteins) that also exert inhibitory effects on the UPS.^326^

While there is no strong evidence that UPS dysfunction is the primary etiological factor in these diseases, its integral role in maintaining proteostasis supports a contribution to their progression. For instance, pancreatic β cell-specific disruption of ERAD in mice leads to disrupted insulin maturation and hyperglycemia.^327^ In myocardial ischemia, there is evidence that the UPS contributes to removing proteins damaged by oxidative stress and that ischemic conditions lead to oxidative damage to proteasome components themselves, with a corresponding accumulation of ubiquitinated proteins.^328^ There is also evidence suggesting that oxidative damage to proteasome components may contribute to decreased proteasome activity in hypertrophic cardiomyopathy and heart failure.^329^ Moreover, under proinflammatory conditions (e.g., exposure to IFNG or TNF), the catalytic components of the proteasome are exchanged, leading to the upregulation of an alternative form of the complex, called the immunoproteasome.^330^ Notably, immunoproteasome upregulation has been associated with aging, and while it enhances the degradation of recombinant tau in the test tube,^331^ an important question is to what extent it exhibits differential activity toward tau aggregates and other substrates in vivo and its significance in neurodegenerative diseases and whether it exhibits age-related declines in activity, as observed for the regular proteasome.^332^

Other major protein degradation pathways converge on the lysosomes, downstream of either endocytosis of extracellular and cell surface proteins, or autophagy of cytoplasmic and organellar contents.^333^ Dysfunction in lysosomal degradation, due to recessively inherited mutations in the genes encoding its enzymes, transporters, or biogenesis factors, results in a range of diseases collectively referred to as lysosomal storage diseases (LSDs), whose common pathology usually involves an accumulation of insufficiently degraded matter inside the lysosomes, and extensive lysosomal failure.^334,335^ For LSDs, cell death or damage, chronic inflammation, and autoimmunity are common etiological components.^335^ In addition, more than two-thirds of LSDs have neurological manifestations, suggesting a significant susceptibility of the nervous system to lysosomal dysfunction.^334^ Since LSDs are rare and generally not comprehensively characterized, it is challenging to evaluate whether disrupted degradation of misfolded proteins specifically contributes to their etiology independently of the general accumulation of undegraded matter within lysosomes. There are several examples in which misfolded proteins may constitute an independent factor.^336^ For example, neurons derived from the skin fibroblasts of a Gaucher’s disease patient demonstrated significant α-synuclein accumulation. This α-synuclein, in turn, disrupts lysosomal GLB1 activity, leading to elevated glucosylceramide levels, which subsequently further promote α-synuclein oligomerization.^337^ This may, however, not be widespread, as it has been reported that ER stress and UPR activation are rarely reported in LSDs. Exceptions exist, such as in GM1 gangliosidosis resulting from beta-galactosidase (GLB1) dysfunction.^338^ Conversely, consistent with the notion that many LSDs result from genetic mutations that disrupt the folding of the encoded protein, it has been observed in vitro that UPR or HSR activation (leading to the upregulation of molecular chaperones) can restore some enzyme activity, e.g., for Gaucher’s disease-associated β-glucocerebrosidase (*GBA1*) mutations and Niemann–Pick disease, type C-associated *NPC1* mutations.^339^

Similar to the UPS, the autophagy–lysosome system also contributes to the degradation of protein aggregates linked to neurodegenerative disorders, such as tau aggregates linked to AD.^340^ Mutations in the E3 ubiquitin ligase TRIAD3A, which facilitates the autophagic removal of tau, have been associated with neurodegenerative disorders such as HD and exacerbate tau aggregation and propagation in a mouse model.^341^ Multiple AD-associated PSEN1 mutations disrupt the targeting of the vacuolar ATPase subunit (ATP6V0A1) to the lysosome, inhibiting lysosomal acidification and the activity of lysosomal proteases.^342^ Notably, these results were linked to impaired ATP6V0A1 glycosylation, and similar effects were also observed when cells were treated with the N-glycosylation inhibitor tunicamycin, revealing how a commonly used experimental method to induce ER stress and UPR signaling may affect other components of the proteostasis network.^342^ In summary, it is evident that the multiple proteolytic pathways of the cell are integral to maintaining proteostasis, and their dysfunction is intimately linked to various diseases.

#### Aging

While aging itself does not directly cause folding disorders or dysproteostasis via mechanisms that are distinct from those mentioned above, most of these mechanisms are associated with an age-correlated increase in frequency, prevalence, or extent. For example, strong links between constitutive inflammation and oxidative stress have been characterized in terms of “inflammaging”^343^ and “free radical theory of aging”.^344^ Moreover, age is a risk factor for proteinopathies such as neurodegenerative diseases, as discussed in Section “Diseases associated with protein folding defects”. Consequently, “loss of proteostasis” is considered one of the hallmarks of aging.^345^

The expression of both autophagic and lysosomal components decreases with age, and disruption of autophagy and endocytosis causes neurodegeneration in mouse models, suggesting a possible explanation for the age dependency of this group of diseases.^345–347^ The age-related declines in degradation activity that have been discussed for the UPS and autophagy–lysosome system are also shared by other proteolytic mechanisms, such as the mitochondrial Lon protease (LONP1), which degrades mitochondrial proteins with oxidative damage (not an infrequent occurrence due to significant ROS generation by the electron transport chain) and whose knockdown causes mitochondrial protein aggregation, leading to mitochondrial dysfunction and disruption.^348^ Concurrent with decreased LONP1 activity in aged murine models, there is an increase in oxidized proteins in the mitochondria.^348^

Another fundamental age-related change that may contribute to dysproteostasis is the accumulation of genetic mutations. Aging involves an ever-increasing cumulative load of DNA damage from replication error and mutagen exposure, coupled with deterioration in DNA repair capacity.^345,349^ For example, human conditions of premature aging (progeroid syndromes) are characterized by loss of function of DNA repair components, such as the DNA helicase WRN, which is mutated in Werner syndrome.^349^ As outlined previously, these mutations may either generate misfolded proteins or introduce loss-of-function mutations in the protein folding, modification, QC, or degradation machinery, with downstream consequences for cellular proteostasis.

Although transcription errors, translation errors, or protein damage are not permanent, such as DNA mutations, due to constant protein turnover by dilution and degradation, they may still accumulate over time. While the majority of proteins exhibit half-lives in the range of a few hours to a few days, others are long-lived and may persist from months to decades.^350,351^ Examples of long-lived proteins include collagen (with a tissue-dependent half-life of ~100–200 years,^352^ crystallins, nuclear pore complex components, histones, and others.^351,352^ Errors in the transcription or translation of these proteins may result in significant accumulation of unfolded/misfolded moieties with potential pathological consequences. Moreover, during aging, proteins may undergo chemical modifications or aggregation that impede or prevent their degradation via the usual mechanisms. The deamidation of Asn and Gln into Asp and Glu, respectively, as well as the epimerization of amino acids from their L to D forms, are referred to as “molecular clocks”, as they accumulate over time in long-lived proteins.^352^ Deamidation may alter the charge and structure of affected proteins and has been implicated in promoting the aggregation of the Aβ peptide, tau, and other proteins that accumulate in brain plaques.^353–355^ Deamidation may also generate neoepitopes that incite autoimmunity, as exemplified by a handful of deamidated peptides that are presented on pancreatic β cells and targeted by T cells in patients with type 1 diabetes.^356^ Similarly, amino acid epimerization may also accelerate protein aggregation: Asp epimerization in the Aβ peptide has been found to interfere with its lysosomal degradation.^357^

Oxidative damage may also promote the accumulation of macromolecules. An example is lipofuscin, a conglomerate of oxidatively damaged lipids and proteins (as well as other constituents) that accumulate with both age and oxidative stress.^358^ Moreover, S-nitrosylation of PDI inhibits its enzymatic activity, abrogates its cytoprotective effects upon proteostasis disruption and is enriched in brain tissue from AD and PD patients compared with control subjects.^359^ As such, one aspect of the age-related decline in proteostasis is the accumulation of altered or damaged protein products.^345^ This is accentuated both by age-associated increases in some of the stresses responsible for damage, as well as a progressive dysfunction in the quality control and degradation pathways that safeguard the proteome against damaged proteins. There are age-related changes in molecular chaperone induction and activity and a trend where basal expression levels of certain chaperones are increased in older animals, whereas the induction of other chaperones by heat shock is diminished (for a review, see^360^). Age-related declines in the UPS,^332,345^ the autophagy–lysosome pathway,^345–347^ and other protein degradation mechanisms^348^ may exacerbate the situation caused by increased protein damage and deficient protein folding. In addition, there is evidence that the capacity of stress response pathways such as the HSR also decreases with age.^361^

#### Experimental perturbation of protein folding and proteostasis

To investigate the mechanisms of protein folding and the cellular response to misfolding, a wide range of chemical agents have been developed to perturb specific folding processes within the ER. These compounds interfere with different aspects of protein maturation, such as glycosylation, disulfide bond formation, calcium-dependent chaperone activity, or protein trafficking, thereby inducing the accumulation of misfolded proteins and triggering the UPR. While these tools provide essential experimental models to dissect folding stress pathways, their pleiotropic effects must be considered when experimental outcomes are interpreted.

As described above, N-linked glycosylation is one of the earliest and most essential modifications assisting protein folding in the ER.^362^ The addition and processing of glycan moieties not only stabilize nascent polypeptides but also serve as molecular signals for quality control pathways. Among the most widely used compounds to perturb this process is tunicamycin, which blocks the first step of glycan precursor synthesis, preventing the addition of glycans to polypeptides.^362^ This leads to the accumulation of unglycosylated, misfolded proteins within the ER lumen, activating all branches of the UPR.

Another frequently used compound, 2-deoxyglucose (2-DG), indirectly impairs glycosylation by inhibiting glycolysis, limiting the production of sugar nucleotides required for glycan synthesis.^363^ Research also suggests that 2-DG-mediated inhibition of glycosylation may come from competition with mannose in the generation of dolichol-linked oligosaccharides and that exogenous mannose treatment is able to ameliorate 2-DG toxicity.^364^ Another glycosylation inhibitor, kifunensine, targets mannosidase I, which leads to the accumulation of high-mannose glycoproteins.^365^ Although kifunensine has been described as a suppressor of the UPR in several studies, it is more likely that it reduces the perceived level of ER stress by preventing misfolded glycoproteins from being recognized as defective, thereby limiting their degradation through the ERAD pathway.

As discussed in Section “HSP100/110 family: Protein disaggregases”, ER calcium stores are fundamental for protein folding and serve as cofactors for numerous folding enzymes and chaperones, including calreticulin and calnexin. Thapsigargin (Tg), a potent and specific inhibitor of the sarco/ER Ca²⁺-ATPase (SERCA), depletes ER calcium levels, severely impairing the folding environment.^366^ Tg-induced calcium depletion rapidly triggers the UPR and downstream stress responses. However, beyond its effects on ER folding capacity, Tg elevates cytosolic calcium levels, activating calcium-dependent signaling pathways that influence apoptosis, gene expression, and mitochondrial function.^367^ For example, Tg-evoked Ca²⁺ signals engage CaMKII, promoting pro-apoptotic JNK signaling and enhancing mitochondrial Ca²⁺ uptake that can precipitate Δψm loss and cytochrome-c release; altered ER–mitochondria Ca²⁺ transfer at contact sites (MAMs) further reshapes mitochondrial metabolism and dynamics.^368^

Additional agents, such as cyclopiazonic acid, inhibit SERCA with lower potency and reversibility, resulting in different kinetic profiles of ER stress induction.^369^ Calcium ionophores such as A23187 and ionomycin increase membrane permeability to calcium, disrupting gradients and contributing to folding stress via calcium imbalance.^370^

Disulfide bond formation is essential for stabilizing the tertiary and quaternary structures of many secretory proteins.^371^ DTT and β-mercaptoethanol are classic reducing agents used to experimentally cleave disulfide bonds, resulting in widespread protein unfolding and UPR activation.^372^ These agents are often applied transiently to study folding stress recovery dynamics, as cells must rapidly reoxidize disulfide bonds upon removal of reductive stress.^373^

Elevated levels of homocysteine, a metabolic intermediate, can also interfere with disulfide bond formation.^374^ Homocysteine may form mixed disulfides with cysteine residues on nascent polypeptides or folding enzymes, leading to their misfolding.^374^ These mechanisms are particularly relevant to disease states like cardiovascular pathology, where homocysteine accumulation is common.^375^

Protein folding within the ER is tightly coupled to trafficking toward the Golgi and eventual secretion.^376^ Interference with these processes can lead to indirect folding stress by causing protein accumulation in the ER.^376^ Brefeldin A (BFA), a well-characterized inhibitor of ER-to-Golgi transport, blocks vesicle formation by targeting guanine nucleotide exchange factors (GEFs), leading to Golgi collapse and retention of cargo proteins within the ER.^372,376^ Similarly, the ionophore monensin disrupts ion gradients within the Golgi, impairing PTMs and folding quality control.^377^

Finally, the physical and chemical properties of the ER membrane itself are critical for maintaining folding homeostasis.^378,379^ Alterations in lipid composition or cholesterol content can compromise the fluidity and structural integrity of the ER, affecting how transmembrane sensors such as IRE1, PERK, and ATF6 detect unfolded proteins.^379^ Such membrane perturbations can alter the sensitivity of the UPR machinery, linking folding stress not only to protein-specific defects but also to metabolic and lipid-associated conditions such as nonalcoholic fatty liver disease.^379^

### Pathological consequences of protein misfolding

In addition to deleterious loss-of-function effects, protein misfolding may have lasting ramifications for cellular homeostasis through toxic gain-of-function. A prominent manifestation of this is when misfolded proteins acquire the ability to partake in extensive and nonproductive protein–protein interactions—that is, to aggregate. The accumulation of such aggregates is a correlative and/or causative feature of multiple disease types, including major neurodegenerative diseases and systemic amyloidoses.^380^ There is also evidence, however, that some species of protein aggregates may have non-pathogenic physiological roles, which will be an interesting avenue for future research.^380^

The formation of protein aggregates usually starts with disordered and homotypic peptide or protein monomers that can self-assemble into a variety of oligomeric structures.^380^ Further aggregation consists of the association or addition of more oligomers or monomers that may lead to the formation of elongated protofibrils/protofilaments, which commonly twist around each other or associate laterally to form longer amyloid fibrils.^380^ The proteins recognized to aggregate upon misfolding are heterogeneous in both sequence and structure.^380^ Nevertheless, the regions that mediate the aggregation of these proteins have some common properties, such as increased hydrophobicity, amphipathic patterning (alternating between hydrophilic and phobic residues), and few charged residues.^380^ Many of the initiating peptides or proteins are intrinsically disordered.^380^

Typically, precursor species exhibit a high propensity for forming β-sheets, and the stacking of these β-sheets constitutes the predominant structural motif of higher-order amyloid fibrils.^380^ These fibrils share a characteristic cross-β architecture, defined by β-strands oriented perpendicular to the fibril’s long axis.^381^ The term “amyloid” itself is, in part, defined by the presence of a cross-β structure. Notably, cross-β fibrils can also arise from α-helical proteins that undergo conformational transitions into β-sheet-rich structures.^381^ In rare cases, cross-α amyloid-like fibrils have been observed, consisting of α-helical segments stacked perpendicular to the fibril axis.^381^ While the elongation of amyloid fibrils often involves conformational changes in the oligomers that initially form, other kinds of protein aggregates, such as amorphous/disordered and native-like aggregates, undergo less restructuring during their elongation.^380,381^ Amyloid fibrils, together with nonamyloid amorphous aggregates and native-like assemblies, represent the three major categories of aggregation end products, all of which are linked to specific diseases.^380^

To describe the kinetics of amyloid fibril formation, several models have been proposed, including nucleated polymerization, nucleated conformational conversion, and native-like aggregation.^382^ A key distinction between the first two lies in whether the initial oligomers serve as elongation-competent nuclei or require further conformational changes to become so.^382^ The last model describes circumstances where the aggregate-spawning monomers are not in a distinctively disordered or aggregation-prone conformation but rather in a native-like conformation where local unfolding or thermal fluctuations may initiate an aggregation cascade that causes downstream conformational changes and fibril elongation.^382^ These different models are all representative of different aggregation processes observed for proteins implicated in conformational diseases.^382^

Amyloid fibrils are structurally polymorphic and exhibit distinct pathological profiles.^383^ Their deleterious effects arise from multiple mechanisms, including pro-inflammatory activity; disruption of cellular morphology; interference with organelle dynamics; sequestration of heterotypic interacting proteins involved in essential processes; and inhibition of proteasomal and autophagic pathways.^383^ Moreover, the consequences of amyloidogenicity are not confined to the cell of origin—these fibrils can also propagate to neighboring cells, contributing to disease spread.^383^ Notably, growing evidence suggests that intermediates along the aggregation pathway may be the primary cytotoxic species in conformational diseases.^380^ Their smaller size and surface exposure of hydrophobic groups facilitate disruptive interactions with membranes and soluble biomolecules, including other proteins, RNA, and metabolites.^381^

### Multiple stress response pathways respond to protein misfolding

In previous sections, we explored the cellular dysfunctions and stressors that contribute to protein misfolding, along with its immediate consequences. Here, we expand the picture by examining key stress response pathways that detect and respond to protein misfolding or upstream stress signals. The proteostasis network, conceptualized as comprising over 1000 proteins, orchestrates protein folding, modification, quality control, and degradation.^384^ Within this framework, the stress response pathways represent a regulatory layer outside the network itself. Specifically, we introduce the HSR, UPR, and mitochondrial UPR (UPR^mt^). These pathways share molecular components that enable crosstalk, prompting us to consider both their specificity and functional overlap.

#### The heat shock response (HSR) senses and reacts to cytosolic dysproteostasis

**Fig. 4:**
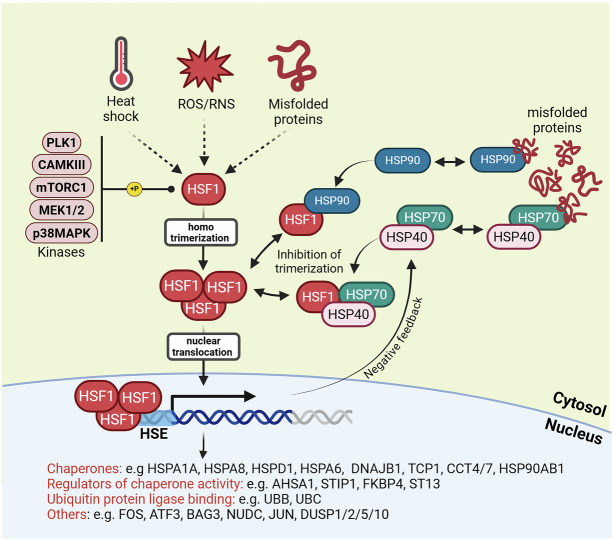
The canonical heat shock response orchestrated by HSF1. Under proteotoxic stress, such as heat shock, reactive oxygen/nitrogen species (ROS/RNS), or accumulation of misfolded proteins, HSF1 is activated through a multi-step process. In unstressed cells, HSF1 is maintained in a monomeric, inactive state through its association with molecular chaperones, particularly HSP90 and HSP70/HSP40. Upon stress, these chaperones are recruited to misfolded proteins, acting as a proteostasis rheostat: as misfolded proteins increase, more HSPs are titrated away from HSF1, allowing its dissociation. Freed HSF1 undergoes trimerization and is phosphorylated by upstream kinases which modulates its activity. The HSF1 trimer translocates to the nucleus, binds to heat shock elements (HSE) in target promoters, and drives transcription of a broad cohort of heat shock genes. As protein folding homeostasis is restored, the newly synthesized HSPs re-bind to HSF1, promoting its inactivation and disassembly—a negative feedback loop that fine-tunes the heat-shock response

It has been estimated that about half of the human proteome is translated on free ribosomes in the cytosol, although this is hard to determine and subject to cell line-specific differences.^385^ A substantial portion of these proteins reside primarily in specific compartments, such as the nucleus or mitochondria, where they need to be translocated to their target compartments post-translationally.^93,386^ During the cytosolic translation of these proteins, the nascent polypeptide chains that emerge from the ribosomes are rapidly bound by chaperones that prevent their aggregation with other unfolded proteins and mediate the next steps in their folding process.^93^ An imbalance in the ratio of chaperones to unfolded proteins, or events that interfere with chaperone activity, may lead to an accumulation of unfolded proteins in the cytosol, which induces the HSR (Fig. 4).^387^ Despite its name, the HSR responds to protein misfolding and dysproteostasis downstream of other stressors than heat shock, as well as to physiological cues that signal incipient stress on the proteostasis network, such as growth factor signaling.^387–390^

The key actor in the human HSR is heat shock transcription factor 1 (HSF1), which mainly resides in the cytosol in a monomeric and inactive state under normal conditions.^361^ The activation and inhibition of HSF1 involves a complex interplay of intra- and intermolecular interactions as well as PTMs; these interactions steer specific steps in HSF1 activation, such as its trimerization, nuclear translocation, DNA binding, association with transcriptional and epigenetic coregulators, and transcriptional activity. For example, the heptad repeat C (HR-C) region of HSF1 may interfere with HSF1 trimerization through intramolecular interactions with its HR-A/B region(s), which mediate intermolecular contacts in the trimer.^361,387^ This autoinhibitory intramolecular interaction is directly regulated by heat-induced conformational changes in HSF1.^391^ While PTMs of the HR-C domain have not been defined, S195 phosphorylation in the HR-A/B domain is associated with abolished autoinhibition by the HR-C domain, and multiple other phosphorylation, acetylation, and SUMOylation events in the other HSF1 domains are important regulators of its activity.^361,390,391^ Moreover, both ROS and RNS, as well as endogenous oxidized or nitrated lipids, can activate HSF1. In the case of ROS, this effect appears to depend on the formation of a disulfide bridge that promotes HSF1 trimerization, whereas RNS-mediated activation may depend on the inhibition of HSP90 through S-nitrosylation.^390^

In tandem with these events, the interaction of HSF1 with molecular chaperones is an important determinant of its activation. The HSP90 complex retains HSF1 in a monomeric state under normal conditions but may preferentially bind misfolded proteins under certain stress conditions, thereby releasing HSF1 from inhibition. HSF1–HSP90 interactions, which are promoted by HSF1 phosphorylation at S121, are also observed for trimeric activated HSF1, suggesting that they also contribute to attenuating the HSF1 response to stress.^361,390^ In addition, HSP70/HSP40 complexes have been reported to negatively regulate DNA-bound HSF1 both by interfering with its ability to bind DNA and its ability to recruit transcriptional coactivators independent of DNA binding. This inhibition may be relieved by increasing the levels of unfolded proteins and reinstated as a negative feedback mechanism downstream of the HSF1 transcriptional response.^361^ A 2018 study in *S. cerevisiae* provided insight into how different stressors may lead to HSR activation through different mechanisms, particularly with respect to whether HSR activation is driven by the absence of HSP90.^392^ Active HSF1 trimers bind heat shock elements (HSEs) in the promoters of target genes and activate their transcription to support an adaptive response against dysproteostasis. HSF1-activated genes include those encoding cytosolic chaperones, such as HSP40 and -70 members (e.g., HSPA1A, HSPA6, HSPB1, DNAJB1, and DNAJB6), as well as regulators of chaperone activity (AHSA1 and STIP1), as well as some ~100 other targets.^388,393^ Many of these latter target genes are related to additional HSF1 functions independent of the HSR, such as developmental processes (e.g., corticogenesis and spermatogenesis) and energy sensing.^389^

Despite its central importance, HSF1 and its downstream target genes are not the only key components of the HSR; many sensors and signaling pathways that contribute to HSR regulation converge on HSF1 or its targets. In humans, two paralogs of HSF1 exist: HSF2 and HSF4. Tissue-specific HSF2 can activate both classical heat shock genes and other targets, either as homotrimers or as heterotrimers with HSF1. HSF4 is more ubiquitous and, apart from HSR, is best known for its role in development and lens physiology.^390^ The importance of HSF2 for CRYAB expression in an HD mouse model, heterotrimerization of HSF1 and HSF2, and inhibition of HSR-related HSF2 activity by HSF4 suggest that both paralogs significantly contribute to maintaining proteostasis together with HSF1.^390,394^ The HSF family also includes fewer characterized members, such as HSF5 and the more distantly related HSFX(1–4) and HSFY(1/2) proteins, which are encoded by genes on the X and Y chromosomes of humans, respectively.^394,395^

As mentioned above, HSF1 is subject to numerous PTMs that govern its activation or inhibition at different levels, and the mediators of these PTMs themselves, or their upstream receptors or sensors, help integrate multiple different stimuli in the HSF1-mediated response.^361,390^ Among the kinases recognized to phosphorylate and activate HSF1 are EEF2K (alias CaMKIII), mTORC1, MAP2K1/-2 (alias MEK1/-2), MAPK14 (p38 MAPK), and PLK1, suggesting that HSF1 activation may be regulated by calcium signaling, energy and nutrient availability, as well as growth factors, cytokines, and other extracellular signaling molecules, including possible coordination with the cell cycle.^390^ For instance, nutrient availability affects HSF1 not only through mTORC1 activation but also via positive regulation by SIRT1 and negative regulation by AMPK.^389^ Given the therapeutic relevance of protein kinases, targeting them with clinically approved inhibitors may offer a tractable strategy to modulate HSF1 indirectly. In cancer cells, for example, MEK inhibitors have been shown to induce cytotoxic amyloidogenesis through downstream suppression of HSF1 activity.^396^

The HSR may play unique roles in various specific tissues. In humans and other animals, specific cell populations are equipped with temperature-sensing capabilities, namely, sensory neurons (thermoreceptors) of the peripheral nervous system that signal to neurons in the pons and, ultimately, the preoptic area of the hypothalamus, which in turn can elicit systemic responses.^397^ While some of the notable responses sparked by such heat sensing are behavioral (e.g., seeking a cooler area) or largely supracellular (vasodilation, sweating, arrector pili relaxation),^397^ evidence from model organisms indicates that HSF1 signaling may be upstream and/or downstream of noncell-autonomous regulation of the HSR in heterotypic tissues (i.e., signaling between different tissues). Most of this research is derived from *C. elegans*, where studies have shown that the overexpression of its HSF1 ortholog, both in neural and glial-like cells, enhances HSF1 signaling and the HSR in peripheral tissues.^398,399^ Moreover, such signaling does not necessarily need to be unidirectional from neurons to peripheral cells, as *C. elegans* germ cells activate an HSF1-based transcriptional response to serotonin release from maternal neurons,^399^ and stressed *C. elegans* embryos induce an HSF1-dependent response in maternal vulval tissue.^400^ Interestingly, inter-tissue stress signaling in *C. elegans* may also occur through HSF1-independent mechanisms, as proteostatic disruption in muscle cells causes chaperone induction in multiple other tissues; furthermore, the overexpression of HSP90 in neural and intestinal tissues could in turn ameliorate dysproteostasis in muscle cells.^401^ Similar organismal coordination of cellular HSF1 or chaperone activity in humans has not been described to date. Certain simplifying aspects of *C. elegans* as a model organism, such as the relative ease of signaling through gap junctions or the pseudocoelom compared with more complex analogous routes of intercellular or inter-tissue communication in humans, may render these mechanisms unique to smaller organisms such as nematodes.^402^ However, owing to the appreciable extracellular input to multiple human stress response pathways, their possible regulation by systemic factors may be an exciting venue for future research.

#### UPR: An adaptive or apoptotic response to ER stress

The ER is a central hub for protein synthesis in eukaryotic cells. It serves as the primary site for the translation of secretory and transmembrane proteins—estimated to comprise one-third of the proteome—as well as many soluble proteins, including cytosolic ones.^385,386^ Consequently, approximately half of the cellular proteome is estimated to be translated on the ER.^385^ Beyond synthesis, the ER plays a critical role in protein folding, quality control (QC), and degradation, supported by a sophisticated array of resident chaperones, glycosyltransferases, oxidoreductases, and other folding machinery. While the ER is well-equipped to maintain proteostasis, excessive influx of nascent proteins or perturbations that impair its folding and QC capacity can lead to the accumulation of misfolded or unfolded proteins—a condition known as ER stress. This stress state activates a three-pronged adaptive response known as the UPR, which aims to restore ER homeostasis.^403–405^

**Fig. 5:**
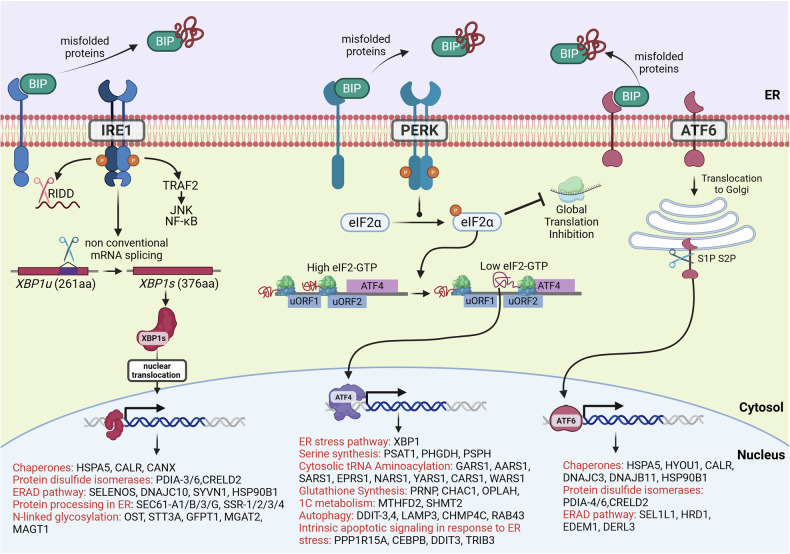
The three canonical UPR pathways signal ER stress to the nucleus. The UPR is a key signaling network that mitigates ER stress through three primary signaling pathways: IRE1α, PERK, and ATF6. Central to this response is HSP70 chaperone BiP (GRP78) that associates with these three proteins to keep them in an inactive state under normal conditions. However, during ER stress, BiP preferentially binds misfolded proteins, releasing and activating the three UPR sensors. Upon activation, IRE1α undergoes oligomerization and induces the unconventional cytosolic splicing of XBP1 mRNA, producing the transcription factor XBP1s, which translocates to the nucleus and upregulates genes involved in protein folding, ERAD, and glycosylation. In addition, activated IRE1 degrades specific RNAs through RIDD (Regulated IRE1 dependent decay). Furthermore, through its kinase function, IRE1α activates TRAF2, which in turn activates the NF-κB and JNK pathways. PERK activation leads to the phosphorylation of eIF2α, globally reducing protein synthesis to prevent further ER overload while selectively enhancing ATF4 translation, which regulates expression of genes associated with amino acid metabolism, oxidative stress response, autophagy, and apoptosis. Upon activation, ATF6 translocates to the Golgi where it is cleaved by S1P and S2P proteases, resulting in the active ATF6 transcription factor that translocates to the nucleus and upregulates expression of genes encoding ER chaperones, lipid biosynthesis enzymes, and ERAD components to increase ER folding capacity. Together, these pathways orchestrate a balance between adaptive stress responses and, if unresolved, apoptotic pathways, ensuring cellular proteostasis under ER stress conditions

The metazoan UPR, including that of humans, is initiated by three transmembrane proteins that contribute to resolving ER stress through both distinct and shared mechanisms.^403–405^ The three stress sensors are inositol-requiring enzyme 1 (IRE1α, also known as ERN1), PKR-like ER kinase (PERK, also known as EIF2AK3), and activating transcription factor 6 (ATF6) (Fig. 5). These stress sensors display a mode of activation similar to both each other and that of HSF1 in the HSR. Under ER proteostasis, the ER has sufficient protein-folding capacity to cover the influx of newly synthesized proteins, and there is an excess of the HSP70 family member BiP (alias HSPA5 or GRP78), which binds to the luminal domains of IRE1α, PERK, and ATF6, analogous to the HSP90-mediated inhibition of HSF1. BiP masks the regions involved in the activation of these sensors, rendering them inactive.^181,403^ Upon ER stress, unfolded proteins accumulate in the ER, leading to gradual dissociation of BiP from IRE1α, PERK, and ATF6, which activates them.^403^ Alternative models have also been posited and suggest, e.g., that additional factors may actively promote BiP dissociation under ER stress.^406^ The levels of active BiP (capable of exerting chaperone activity or inhibiting the three UPR sensors) are also buffered by at least two mechanisms: (1) homo-oligomerization of BiP under low levels of unfolded proteins and (2) BiP AMPylation at T518 by the protein adenylyltransferase FICD.^403^ This process and the downstream activation steps of the three stress sensors diverge in their mechanisms and are introduced individually below.

##### The IRE1α pathway and its relationship with XBP1, RIDD, and JNK

IRE1α is an ER-resident type I transmembrane protein with a luminal stress-sensing domain and dual cytosolic effector domains: one domain confers protein serine/threonine (S/T) kinase activity, while the other domain provides endoribonuclease (RNase) activity.^407^ Notably, outside of an observed in vitro phosphorylation of a heterologous peptide, the only recognized target of IRE1α protein kinase activity is IRE1α itself. Autophosphorylation of IRE1α is a prerequisite for the activation of its RNase activity.^408^ The close paralog of IRE1α—IRE1β (ERN2), which is expressed predominantly or exclusively in epithelial cells of the gastrointestinal (GI) tract—exhibits similar but diverging RNase activities to IRE1α and can even antagonize IRE1α activity.^409^

Historically, inactive IRE1α has been thought to exist as monomers bound and inhibited by BiP, with activation involving homodimerization and trans-autophosphorylation following BiP dissociation.^410^ Beyond these homodimers, larger IRE1α oligomers—specifically back-to-back dimers—have also been identified. Subsequent studies suggested that these higher-order oligomers may be essential for full RNase activity, whereas the dimeric form may represent an inactive or less active state of the enzyme.^411^ Previously, it has also been proposed that IRE1α dimers and oligomers may differentially contribute to XBP1 splicing and RIDD, with the former possibly being more dependent on higher-order IRE1α oligomerization.^412^ Another model in which the direct binding of unfolded proteins to a luminal peptide-binding groove of IRE1α promotes its dimerization and activation has also been suggested.^410^ This model was initially based on studies of yeast Ire1 and was suggested not to apply to human IRE1α owing to their structural differences. A later study, however, also revealed direct binding of the human IRE1α luminal domain to unfolded proteins, causing conformational changes and promoting its oligomerization and RNase activity.^410^ Thus, the relative importance of the direct and indirect effects of unfolded proteins on IRE1α activation—through direct binding of unfolded proteins or BiP dissociation, respectively—is still not fully understood.^403^ Independent of its luminal domain, IRE1α may also be activated by altered ER membrane lipid composition through its transmembrane domain.^413^ In addition, other pathways may regulate IRE1α activity, such as TLR2- and TLR4-mediated activation,^414,415^ AKT1-mediated inhibition,^414,415^ or ERK1/-2 (MAPK3, MAPK1)- and AKT1-mediated stabilization.^416^

IRE1α RNase activity mediates two distinct functions: unconventional (extranuclear) splicing of the XBP1 transcript and the cleavage of other cytosolic RNA species (e.g., mRNAs and miRNAs), with the latter termed regulated IRE1-dependent decay (RIDD). IRE1α RNase activity targets RNAs containing the CUGCAG consensus sequence in a stem loop (present, e.g., XBP1) but has also been found to cleave RNAs without this motif.^417,418^ RIDD may therefore not exclusively target a specific subset of RNAs but could exhibit some nonspecific activity with more general effects on the cellular transcriptome. An in vitro cleavage assay in H929 cells upon ectopic expression of IRE1α and RNA sequencing (RNA-seq) identified 863 putative RIDD target mRNAs.^417^ Among 32 selected mRNAs, 11 had the CUGCAG consensus sequence in a steam-loop motif at the observed cleavage site, and their expression was downregulated upon ectopic IRE1α expression.^417^

The best characterized role of IRE1α is in the activation of the transcription factor XBP1s (Fig. 5). While the native XBP1 transcript is translated into a functional protein (XBP1u; u = unspliced), its cleavage at two sites by IRE1α excises a 26 nucleotide intron that leads to a frameshifted transcript encoding the functionally distinct XBP1s (s = spliced) protein, which is an active transcription factor.^404^ After IRE1α-mediated cleavage of XBP1, the transcript is reconstituted to form XBP1s mRNA by the RNA ligase RTCB; this step may be regulated by RTCB phosphorylation.^419^ As such, XBP1u is the dominant protein isoform in unstressed cells (albeit modestly expressed), whereas ER stress and UPR induction leads to XBP1 mRNA upregulation and increased XBP1s protein levels. This is followed by XBP1u protein upregulation, as elevated XBP1 mRNA levels persist while IRE1α activity diminishes.^420^ The two XBP1 isoforms may be functionally antagonistic: XBP1u represses some XBP1s target genes (e.g., BiP), possibly through cytosolic sequestration and increased degradation of XBP1s.^420^

XBP1s activates the transcription of genes that contain ER stress response elements (ERSE, ERSE-II) or UPR elements (UPREs) in their regulatory regions.^421^ Among the genes activated by XBP1s are those that encode molecular chaperones (HSPA5, ERO1B, DNAJB9, DNAJC3, and PDIA6), ER quality control/ERAD components (EDEM1, UGGT1, and ERLEC1), and UPR signaling components themselves (XBP1 and ATF6).^422,423^ Some of the XBP1s target genes are also under regulation by other UPR pathways; moreover, XBP1s can participate in transcriptional programs regarded as distinct from the UPR (e.g., plasma cell differentiation^424^ or proinflammatory signaling in macrophages^414^). A 2016 Perturb-seq study has helped elucidate the individual and joint transcriptional activities of the IRE1α, PERK, and ATF6 arms of the UPR under ER stress.^423^ While XBP1s contributes to reinstating proteostasis by upregulating genes involved in ER protein import, folding, and quality control, it likely also exerts negative feedback on the IRE1α arm through its upregulation of DNAJB9, which promotes the inhibitory interaction of BiP with IRE1α.^148^

In addition to instigating the XBP1s-mediated transcriptional response, activated IRE1α also serves as a scaffolding protein that facilitates the activation of other signaling pathways. In the best characterized example, the cytoplasmic domain of IRE1α recruits TRAF2 and ASK1 (alias MAP3K5), leading to downstream activation of JNK (MAPK8) under ER stress.^425,426^ In mouse embryonic fibroblasts, IRE1α-mediated JNK activation under ER stress is dependent on tumor necrosis factor receptor 1 (TNFRSF1A).^427^ In general, JNK activation is tightly linked to proapoptotic signaling through mechanisms such as phosphorylation and activation of the proapoptotic Bcl-2 family members BIM (BCL2L11), BMF, or BAD; in addition, it can activate apoptosis through the phosphorylation of the transcription factor JUN and the downstream upregulation of proapoptotic genes, including TNF, FASLG, and BAK1.^428^ Consistent with this, IRE1α–JNK signaling is considered a contributing factor to apoptosis upon prolonged or excessive ER stress.^404,429^ The IRE1α–XBP1s axis may also positively regulate c-JUN expression through downregulation of miR-216b.^430^ However, a study on a liver-specific IRE1α knockout (KO) mouse model revealed c-JUN upregulation under ER stress only in IRE1α-KO livers but not in WT livers, suggesting that the positive regulation of c-JUN is not universal and may be context dependent.^431^

IRE1α scaffolding activity may also lead to the activation of p38 MAPK (MAPK14), ERK1 (MAPK3), and NF-κB (NFKB) signaling, although these links have not been extensively studied and validated.^432,433^ Different studies have pointed to the importance of IRE1α PTMs for its scaffolding activity. For example, a kinase-inactive IRE1α mutant has been shown to partially hinder JNK activation under ER stress.^425^ Additionally, STUB1-mediated ubiquitination of IRE1α may be required for its phosphorylation and TRAF2 recruitment during ER stress^434^, while IRE1α sulfenylation appears necessary for TRAF2/ASK1/p38 activation (as well as being mutually exclusive with IRE1α phosphorylation and XBP1 splicing) under oxidative stress.^435^ While further studies may be necessary to reconcile all these observations, collectively, they still allude to the importance of PTMs in regulating the activities of IRE1α. In addition to the interplay of IRE1α with JNK, its RIDD activity is also associated with proapoptotic effects, e.g., through the cleavage of miR-17 and other miRNAs to relieve the suppression of TXNIP and CASP2.^418^ In summary, the activities of IRE1α under ER stress may have both adaptive and proapoptotic effects, depending on the external modulation of IRE1α itself or on the kinetics of the pathways it initiates.

##### The PERK pathway intersects with the integrated stress response (ISR) at eIF2α phosphorylation

PERK is a type I transmembrane ER protein containing a luminal domain similar to that of IRE1α and a cytosolic S/T protein kinase domain; this latter domain, unlike IRE1α, has multiple recognized targets, including both itself and other proteins, such as eIF2α and NRF2 (NFE2L2).^403,405^ Moreover, PERK also has lipid kinase activity and may catalyze the conversion of diacylglycerol to phosphatidic acid.^436^ Like IRE1α, PERK is normally inhibited by BiP binding, and its activation consists of BiP dissociation, homodimerization, and trans-autophosphorylation, allowing the recruitment and phosphorylation of other target proteins.^403–405^ Furthermore, PERK may also share other modes of activation with IRE1α, such as direct binding to unfolded proteins^437^ or an altered lipid composition of the ER membrane.^413^

The best characterized pathway downstream of PERK activation involves PERK-mediated phosphorylation of eIF2α (EIF2S1) (eukaryotic translation initiation factor 2 subunit alpha) at S51 (Fig. 5). eIF2α must not be confused with EIF2A, which also participates in translation initiation, albeit only for specific mRNAs in a codon-dependent manner.^438^ eIF2α, eIF2β (EIF2S2), and eIF2γ (EIF2S3) together form the EIF2 heterotrimer.^439^ EIF2, in turn, is part of the 43S preinitiation complex (43 PIC), which consists of the 40S small ribosomal subunit, together with the initiation factors eIF1, eIF1A, eIF2, eIF3, eIF5.^439^ The 43 PIC is recruited to the 5’-mG cap of mRNAs by the cap-binding EIF4E protein of the EIF4F complex.^439,440^ GTP-bound EIF2 is responsible for the binding and delivery of Met-tRNAi^Met^ to the 43 PIC, where this initiator tRNA base pairs with the mRNA start codon AUG and delivers methionine as the first amino acid of the nascent polypeptide chain. After Met-tRNAi^Met^ dissociation, EIF5 hydrolyzes the GTP of EIF2 to GDP, and EIF2 dissociates from the 43 PIC.^439,441^ Recycling of EIF2-GDP to allow its recruitment of a new Met-tRNAi^Met^ and initiation of a new translation process requires the guanine nucleotide exchange factor (GEF) activity of the EIF2B α/β/δ/ε/γ heteropentamer.^441^ EIF2B is not to be confused with eIF2β—the beta subunit of EIF2. For comprehensive reviews of eukaryotic translation initiation, see^439^ and.^440^

PERK is one of the four mammalian eIF2α (EIF2S1) kinases that interferes with translation initiation by phosphorylating eIF2α at S51; the others being HRI (EIF2AK1), PKR (EIF2AK2), and GCN2 (EIF2AK4), which are activated by distinct upstream stimuli.^319,440^ This general mechanism of eIF2α S51 phosphorylation by an eIF2α kinase is referred to as the integrated stress response (ISR), as it can integrate many different upstream stimuli into a coordinated stress response.^319^ However, due to each kinase having multiple downstream targets, the different contexts in which they may be activated and their effects on other cellular pathways, the ISR has different characteristics and outcomes depending on its mechanism of activation.^319^

S51-phosphorylated eIF2α binds EIF2B with higher affinity, sequestering it and preventing it from catalyzing the recycling of the EIF2 ternary complex.^441^ Therefore, the translation of mRNAs that require cap recognition by the EIF4F complex for translation initiation is inhibited, and the global rate of translation is reduced, decreasing the cellular load of nascent and unfolded proteins. In contrast, certain mRNAs (e.g., the ISR effector ATF4) contain short upstream open reading frames (uORFs) in their 5’ untranslated region (UTR), which promotes their translation under conditions of eIF2α phosphorylation.^442^ In the absence of eIF2α phosphorylation, the ribosome translates these uORFs, as active EIF2B allows for EIF2 recycling before the ribosome reaches the next uORF. This prevents translation of the ATF4 coding sequence (CDS), as the last uORF extends multiple codons into the ATF4 CDS in a –1-frameshifted manner.^440^ Consequently, the ribosome may not reinitiate translation of the ATF4 transcript from its bona fide start codon. However, when eIF2α is phosphorylated and EIF2B activity is decreased, EIF2 recycling proceeds more slowly, and for a substantial number of ribosomes, it occurs after the start site of the last uORF but before the ATF4 CDS, allowing translation of the full-length protein.^440^ Therefore, ATF4 is upregulated upon ER stress or other stress conditions that activate the ISR.^319^

ATF4 shares this mode of regulation with multiple other transcripts that are preferentially expressed under ER stress or the ISR, and many of these genes are also transcriptionally activated by ATF4, thereby requiring both ATF4 and eIF2α phosphorylation for their expression. Examples of this include GADD34 (PPP1R15A)^443^ and CHOP.^444^ GADD34 is a regulatory subunit of protein phosphatase 1 that promotes eIF2α dephosphorylation and thereby mediates negative feedback on PERK/ATF4 signaling.^403^ While GADD34 may help attenuate the UPR before it progresses to an apoptotic phase, its reactivation of translation may also exacerbate ER stress to a cytotoxic effect under persistent dysproteostasis.^445^ Consistent with this notion, pharmacological inhibition of eIF2α dephosphorylation with salubrinal is associated with increased survival of, e.g., rat PC12 cells under ER stress.^446^

In addition to being a target gene, CHOP is one of the principal transcriptional coregulators of ATF4. A ChIP-seq study in mouse embryonic fibroblasts (MEFs) identified 321 CHOP-bound genes and 472 ATF4-bound genes, with 218 targets shared between the two.^447^ Consistent with this overlap, RNA-seq analysis of ER-stressed MEFs lacking either ATF4 or CHOP revealed multiple regulatory patterns: some genes were similarly affected by both knockouts, while others were oppositely regulated or uniquely responsive to only one factor.^447^ It revealed that both ATF4 and CHOP positively regulate genes involved in translation (e.g., initiation factors and aminoacyl-tRNA synthetases), in addition to more ATF4-specific contributions to amino acid biosynthesis (e.g., ASNS, PSAT1, and PHGDH) and transport (e.g., SLC7A11, SLC38A2, and SLC7A1).^447^ Moreover, the study revealed mixed contributions of ATF4 and CHOP to ER protein folding and quality control processes through positive regulation of genes such as BiP, PIN1, SEL1L, PDIA4, SERPINH1, etc., as well as the other major UPR transcription factors XBP1 and ATF6.^447,448^ These observations are in line with other studies,^448^ including the targeted UPR perturb-seq study, which identified the PERK/ATF4 axis as having the largest unique regulon among the UPR arms (including its influence on amino acid homeostasis and translation) while also contributing to canonical UPR responses (bolstering protein folding and quality control) together with IRE1α/XBP1s and ATF6.^423^ A notable feature of the ATF4 regulon is its inclusion of many ATF4 transcriptional coregulators, such as ATF3, CHOP, NRF2, and CEBPB/-D/-G, which may have implications for how the ATF4 transcriptional program is modulated over time.^448^ Owing to its importance as a convergence point for the ISR, the range of influence of ATF4 also extends to the mitochondrial proteome and to genes involved in autophagy^448^ — points that will be revisited in subsequent sections of this article.

Like the IRE1α/XBP1s axis, PERK/ATF4 signaling is also implicated in mediating the apoptotic response to persistent and excessive ER stress.^429^ ATF4 increases the expression of the proapoptotic Bcl-2 family members PUMA (BBC3), NOXA (PMAIP1), and MCL1,^448^ which are generally antiapoptotic unless alternatively spliced.^449^ Moreover, downstream of ATF4 (and to some extent also downstream of XBP1s and ATF6^429,450^), CHOP contributes to proapoptotic signaling through the activation of genes such as BIM (BCL2L11), DR5 (TNFRSF108), PUMA, BAX, CHAC1, and TRIB3, as well as the downregulation of antiapoptotic BCL2.^429^ CHOP deletion has been shown to have cytoprotective effects in multiple mouse models of diseases characterized by constitutive ER stress and UPR activation,^445,450^ suggesting that it may be an important transducer of proapoptotic UPR signaling in several contexts. This is further supported by observations that PERK/ATF4/CHOP activity, but not IRE1α or ATF6 activity, is sustained upon prolonged ER stress, leading to apoptosis, with PERK activation further impairing cell viability, whereas IRE1α activation improves it.^451^ However, the extent to which proapoptotic UPR signaling is mediated primarily by individual genes or by the integrated output of entire signaling pathways, as well as how this process varies across different contexts, remains to be determined. For example, ATF4 and CHOP target TRIB3, which is another gene whose deletion decreases UPR-associated apoptosis, despite exerting negative feedback on ATF4 and CHOP signaling,^452^ similar to the feedback from GADD34 and the effects of its inactivation.^445^ Consequently, UPR activity appears to consist of delicately balanced adaptive and proapoptotic output. While this complicates efforts to delineate the UPR, it also suggests that the stress response may be sensitive to perturbations and a suitable target for therapeutic interventions.

Finally, we revisited the activities of PERK beyond eIF2α phosphorylation. The next best characterized substrate of PERK is NRF2, whose PERK-mediated phosphorylation prevents its binding to KEAP1, thereby relieving its cytosolic sequestration and allowing its nuclear translocation to transcriptionally regulate target genes as a key part of the antioxidant response.^453^ Notably, similar to CHOP, NRF2 is also both a target gene and a transcriptional coregulator of ATF4, establishing it as an important effector of the PERK arm of the UPR.^448^ Moreover, ATF4 and NRF2 may cooperate in other ISR pathways, such as under certain amino acid deficiencies, where GCN2 activates ATF4, and a lack of precursors for glutathione synthesis (glutamate, cystine) leads to NRF2 activation.^454^ The principal targets of NRF2 mediate antioxidant activities and detoxification, such as glutathione biosynthesis enzymes (GCLC, GCLM), glutathione S-transferases (GSTs), redoxins (TXN, SRXN1), and UDP-glucuronosyltransferases (UGTs), and more.^455^ While ATF4 and NRF2 share some target genes (SLC7A11, HMOX), their regulons appear to be mostly distinct from each other.^448,454,455^ In fact, some of their transcriptional programs may be antagonistic, such as the induction of glutathione-cleaving CHAC1 by ATF4, which counteracts NRF2-driven expression of glutathione biosynthesis genes but may also serve to maintain NRF2 activation through this mechanism.^454^ As such, NRF2 may perform functions downstream of PERK that the eIF2α/ATF4 axis alone is not capable of handling. In addition to NRF2, PERK may be involved in the activation of the pathways described above, such as p38 MAPK and NF-κB, which are also downstream of IRE1α scaffolding activity.^456,457^

##### The ATF6 pathway

The third arm of the UPR is perhaps also the least complex, as only one protein—the transcription factor ATF6—takes on both the sensor and effector roles. ATF6 is an ER-resident type II transmembrane protein with a basic-leucine zipper (bZIP) domain in its N-terminal/cytoplasmic half.^405^ Like IRE1α and PERK, its luminal region is bound by BiP, which dissociates upon ER stress to expose ATF6 Golgi localization signals, allowing the initiation of events that constitute its activation.^405^ Following BiP dissociation, ATF6 is trafficked to the Golgi, where it undergoes sequential cleavage by Golgi-resident site 1 and site 2 proteases (S1P/MBTPS1 and S2P/MBTPS2, respectively), releasing a 50 kDa cleavage product that corresponds to the cytoplasmic half of ATF6 (referred to as ATF6p50 or ATF6-N [N = nuclear]), which subsequently translocates to the nucleus to mediate transcriptional regulation.^405^

Evidence indicates that the reduction of ATF6 disulfide bridges by the ER oxidoreductase TXNDC12 (also known as ERp18) may facilitate ATF6 processing by S1P, as both S1P mutants and TXNDC12 knockout result in an additional 70 kDa ATF6 product and a diminished induction of ATF6 target genes^181^; a similar role in ATF6 activation has also been proposed for the disulfide isomerase PDIA5.^458^ Additional mechanisms have also been proposed to enhance ATF6 activation, such as ATF6 hypoglycosylation or interactions with members of the thrombospondin family of proteins.^405^ Notably, the IRE1α and PERK arms may also contribute to ATF6 activation in multiple ways. For example, both ATF4 and XBP1s can activate ATF6 transcription, at least modestly.^405^ Moreover, both ATF4 and XBP1s transcriptionally regulate multiple genes involved in ER-to-Golgi transport, which, albeit not being ATF6-specific, could help maintain the integrity of a central step in ATF6 activation.^459,460^ Finally, p38 MAPK, whose induction under ER stress may be dependent on both IRE1α- and PERK, can contribute to regulating the nuclear translocation and activity of ATF6.^461^

Like XBP1s, ATF6 binds ERSE and ERSE-II motifs in the regulatory elements of its target genes (and possibly also UPREs) and consequently exhibits an overlapping regulon with XBP1s; however, compared with XBP1s, ATF6 may be a more powerful inducer of ERSE-regulated genes but a less powerful inducer of ERSE-II-regulated genes.^421^ In contrast to XBP1s, there is conflicting evidence regarding whether ATF6 binds UPREs.^421,462^ ATF6 additionally recognizes cAMP response elements (CREs), which, together with their different coregulators and affinities for regulatory elements, help differentiate the contributions of XBP1s and ATF6 to the overall UPR transcriptional program.^421^ Interestingly, the available evidence suggests that few UPR target genes are activated solely by ATF6, independent of contributions from XBP1s or ATF4. For example, an early microarray study indicated that ATF6 knockdown in MEFs had little impact on the expression of tunicamycin-inducible genes^422^; the perturb-seq study mentioned above also supported the notion that many ATF6 target genes are under joint regulation by either XBP1s or ATF4, although ATF6 frequently exhibited the largest contributions to their expression.^423^ Other findings emphasize that ATF6 deletion still impairs the net adaptive capacity of the UPR and is deleterious for stressed cells.^462^ This finding corroborates the earlier point about the sensitivity of the UPR to perturbations, as apparently redundant mechanisms, when disrupted, have dramatic consequences for the cell. Among the ATF6 target genes, we find molecular chaperones (BiP, HSP90B1, and DNAJC3) and ER quality control components (CALR, EDEM1, and HERPUD1), which is consistent with the categories of genes induced by XBP1s and the general output of the UPR.^405,423,462^ Interestingly, ATF6 has also been shown to regulate the expression of several oxidative stress-associated genes, including genes encoding the antioxidant proteins catalase (CAT), PRDX5, and SELENOS, which are not among the proteins traditionally linked to the antioxidant activities of the ER.^463^ Moreover, like the IRE1α and PERK arms, ATF6 has also been implicated in the activation of NF-κB signaling (possibly through AKT1) upon treatment of cells with the subtilase cytotoxin.^464^

#### The mitochondrial unfolded protein response (UPR^mt^) and other mitochondrial stress responses

As noted above, the majority of mitochondrial proteins are synthesized in the cytosol and imported into mitochondria in an unfolded conformation.^465^ Successful mitochondrial protein folding requires coordinated actions of mitochondrial-resident molecular chaperones, which mediate the unfolding and subsequent refolding of proteins during and after their translocation into mitochondria.^466^ Additionally, mitochondrial proteases cleave presequences from matrix-targeted proteins, a process crucial for correct protein folding and maturation.^466^ However, mitochondria present unique challenges to proteostasis, including oxidative damage caused by reactive oxygen species (ROS) from the respiratory chain and constant remodeling, fusion, and fission events according to metabolic demands.^303,466^ Thus, mitochondrial proteostasis relies on dedicated constitutive and inducible processes collectively termed mitochondrial stress responses.

**Fig. 6:**
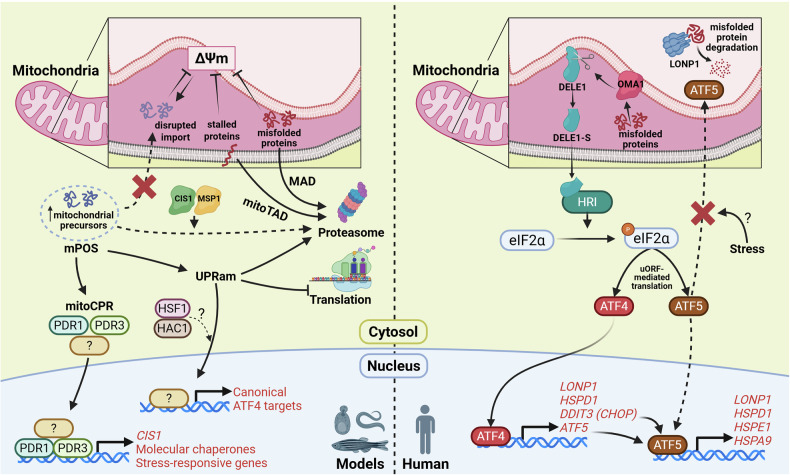
Mitochondrial UPR via the DELE1–HRI–eIF2α axis and downstream ATF4/ATF5 signaling. Left: In common model organisms, multiple processes that contribute to mitochondrial proteostasis have been characterized, a selection of which are illustrated here. Disrupted or stalled protein import, as well as protein misfolding, can perturb the mitochondrial membrane potential (ΔΨm), which exacerbates challenges to mitochondrial proteostasis. Misfolded and stalled mitochondrial proteins are subjected to proteasomal degradation through MAD and mitoTAD, respectively. A cytosolic accumulation of mitochondrial precursor proteins is termed mPOS and activates the mitoCPR and UPRam. mitoCPR is characterized by increased expression of molecular chaperones and other stress-responsive genes, as well as a multidrug resistance-like transcriptional program activated by PDR1 and PDR-3. mitoCPR also enhances the expression of CIS1, which may promote the degradation of mitochondrial precursors together with MSP1. The UPRam is associated with a general decrease in protein synthesis and increase in protein degradation, as well as a gene expression program resembling canonical ATF4 target genes. Other UPRam-mediated changes in gene expression may be linked to HSF1 or HAC1 activity. Since the figure represents pathways from multiple model organisms, human gene and protein nomenclature is used. Right: In human cells, a more unified model of the mitochondrial stress response (UPR^mt^) has been characterized as relying on the ISR. A mitochondrial accumulation of misfolded proteins can activate the OMA1–DELE1–HRI axis, leading to a reduction in general translation, but an increase in the translation of uORF-regulated transcripts. These include the transcription factors ATF4 and ATF5, which activate the expression of molecular chaperones and other factors that can ameliorate mitochondrial dysproteostasis. ATF5 activation may also be enhanced by de-sequestration when its mitochondrial localization is disrupted by mitochondrial stress, putatively increasing its nuclear occupancy

Mitochondrial protein folding stress arises predominantly from impaired protein import or intramitochondrial perturbations that disrupt protein folding, maturation, or stability.^467^ Unlike ER stress pathways, mitochondrial stress responses are more diverse and are activated by distinct triggers rather than a single unified stimulus. These triggers commonly disrupt the mitochondrial membrane potential (ΔΨm) and electron transport chain integrity.^467^ While extensively studied in model organisms (Fig. 6, left), particularly yeast and *C. elegans*, the conservation and mechanisms of these pathways in human cells remain somewhat unresolved.

In yeast, mitochondria-associated degradation (MAD)—analogous to ERAD—ensures proteostasis by extracting, ubiquitinating, and degrading misfolded mitochondrial proteins in the cytosol.^170,468^ A specialized form called mitochondrial translocation-associated degradation (mitoTAD) specifically handles proteins stalled during import.^469^ Excess accumulation of mitochondrial precursors due to import dysfunction results in mitochondrial precursor overaccumulation stress (mPOS).^470^ This triggers at least two defined responses: UPRam (UPR activated by mistargeted proteins), which involves translational attenuation and increased proteasomal activity, and mitoCPR (mitochondrial Compromised Protein Response), which includes a multidrug resistance-like response governed by PDR3 and possibly PDR1—transcription factors with no clear human orthologs.^471,472^

An intriguing question is why the HSR does not appear to be prominently involved in responding to mPOS. The original studies characterizing UPRam and mitoCPR included transcriptomic analyses that can be used to evaluate transcriptional signatures. Our unpublished reanalysis of these datasets (conducted for the purpose of this review) suggests potential contributions of HSF1 and HAC1 (the yeast ortholog of XBP1s) to the upregulated genes in the UPRam response. In contrast, mitoCPR exhibits a more diverse transcriptional profile, including upregulation of molecular chaperones and other stress-inducible genes responsive to heat shock, oxidative stress, and nutrient deprivation, alongside canonical PDR1/3 targets. Notably, only 18 transcripts were commonly upregulated across the two studies, underscoring the distinct nature of these stress responses.

Interestingly, a zebrafish study employing the same experimental perturbation as the yeast UPRam model—expression of mutant MIA40—did not reveal a direct overlap in upregulated transcripts. Instead, transcriptomic enrichment analyses highlighted pathways such as aminoacyl-tRNA synthetases, glutathione metabolism, molecular chaperones, one-carbon metabolism, and other canonical ATF4 targets. This divergence suggests that while mitochondrial import stress elicits a conserved cellular concern across species, the transcriptional programs mobilized in response may be highly context dependent.

A deeper understanding of how mitochondrial folding stress is sensed and transduced in human cells has been enabled by high-throughput approaches such as perturb-seq. A landmark study utilized single-cell RNA sequencing (scRNA-seq) to profile transcriptomic changes following genome-wide CRISPR interference (CRISPRi) in human K562 cells.^473^ Among the 1,973 gene perturbations that induce strong transcriptional responses, those affecting the mitochondrial protein import machinery—including MIA40 (CHCHD4), TOMM22, TIMM23B, and components of the PAM and SAM complexes—elicited a distinct expression signature.^473^ Notably, this signature clustered closely with perturbations of tRNA synthesis and components of the EIF2 complex, all of which activated canonical stress programs such as the integrated stress response (ISR), mTORC1 signaling, and nonsense-mediated decay pathways while concurrently suppressing oxidative phosphorylation and mitochondrial translocation-related gene expression. These findings were corroborated by an independent study in mice where disruption of the mitochondrial ribosomal subunit MRPS5 produced a similar transcriptional landscape, supporting a common response to disturbances in mitochondrial translation and import.^474^

Strikingly, in the Perturb-seq study, perturbations of mitochondrial translation and translocation correlated more strongly with tRNA synthesis and EIF2 complex inhibition than with disruptions of cytosolic translation, despite the latter affecting a much broader portion of the proteome.^473^ This correlation of tRNA synthesis/EIF2 perturbation and perturbation of mitochondrial translation or translocation may point to a particularly important role for the ISR in maintaining mitochondrial homeostasis, given that the ISR is a downstream event of both EIF2 insufficiency and ribosome stalling.^319,475^

These features in the Perturb-seq data elegantly recapitulate the main findings of previous studies showing that the ISR also constitutes the backbone of a stress response to various mitochondrial insults in human cells, sometimes referred to as a mitochondrial unfolded protein response (mtUPR or UPR^mt^).^476,477^ While it was recognized already in 2002 that CHOP mediated a nuclear response to protein aggregation in the mitochondrial matrix,^478^ recent findings established the canonical upstream regulator of CHOP, ATF4, as a major mediator of the UPR^mt^ (Fig. 6, right). Under mitochondrial stress caused by oligomycin-mediated respiratory chain inhibition, the OMA1 protease cleaves DELE1 at the inner membrane, followed by cytosolic accumulation of the DELE1 cleavage product and its interaction with HRI (EIF2AK1), enhancing the eIF2α kinase activity of HRI based on cell-free in vitro studies.^477^ However, while DELE1 fragments were found to increase HRI activity and induce ATF4 expression under oligomycin treatment, OMA1 or DELE1 knockdown surprisingly enhanced eIF2α phosphorylation in oligomycin-treated HEK293T cells. This could indicate that DELE1 fragments also participate in negative feedback on eIF2α phosphorylation and/or directly affect ATF4 mRNA translation under stress conditions^477^; regardless of the mechanism, this finding indicates an additional complexity in OMA1/DELE1/HRI-mediated UPR^mt^ activation that is yet to be characterized.

ATF4 regulates several coregulators contributing to the human UPR^mt^, including CHOP and its paralog ATF5, which promote the expression of mitochondrial chaperones and the LONP1 protease under mitochondrial stress.^479^ While overexpression of human ATF5 can rescue the UPR^mt^ in *C. elegans* deficient in its ortholog ATFS-1—perhaps the principal regulator of the UPR^mt^ in this model—its role in the mammalian UPR^mt^ remains less clear, with evidence suggesting that ATF4 may act as the dominant effector under most mitochondrial stress conditions.^476,479^ Nonetheless, ATF5 may play a role in an ATF4-independent, HSF1-mediated UPR^mt^ that is induced by the mitochondrial HSP90 inhibitor GTPP or the LONP1 and HTRA2 protease inhibitors CDDO and Ucf-101, respectively.^480^ Like *C. elegans* ATFS-1 (although weaker), human ATF5 also contains a mitochondrial localization signal, suggesting that the same mechanism, consisting of its mitochondrial sequestration under basal conditions and cytosolic and nuclear accumulation under mitochondrial stress, could play a role in human cells.^479^ Additional signaling pathways, including those involving PKR (EIF2AK2), PERK (EIF2AK3), lysosomal acidification, and sirtuin-regulated antioxidant programs, have also been implicated in shaping the mitochondrial stress response.^477,481–483^

In summary, mitochondrial protein folding stress may arise, e.g., from disrupted protein import, oxidative protein damage, protein misfolding, and perhaps more generally, perturbations to the inner membrane potential. The UPR^mt^ integrates organelle-specific surveillance with cytosolic stress pathways—particularly the ISR—to restore mitochondrial proteostasis. While considerable mechanistic insight has been gained from model systems and high-throughput transcriptomic screens, further work is needed to resolve the cell type- and context-specific elements of this response in mammals and to clarify how folding-specific signals are distinguished from other mitochondrial insults.

## Diseases associated with protein folding defects

It is now well established that protein misfolding and aggregation are the main or contributing etiological factors for a multitude of diseases (Fig. 3).^345,384^ These diseases—particularly neurodegenerative diseases—are often referred to as proteinopathies or conformational diseases. A subset of proteinopathies is characterized by an accumulation of amyloid fibrils and is referred to as amyloidoses. In addition, protein misfolding may be exacerbated secondary to a preexisting disease condition and contribute to disease progression or additional pathology. Protein folding defects and dysproteostasis have also emerged as key factors in cancer. This section explores the links between protein misfolding and several major disease categories.

### Neurodegenerative diseases

Neurodegenerative diseases (NDDs) are major global burdens of disease and are expected to continue to rise in incidence due to increasing life expectancy. In 2021, the global age-adjusted mortality of neurological disorders was estimated to be ~33/100,000, making it the seventh most prevalent cause of death among the major disease groups.^484^ NDDs include diseases such as AD, PD, Huntington’s disease (HD), and ALS, which are characterized by a progressive dysfunction and loss of neurons in specific brain regions, leading to symptoms such as cognitive and motor deficits (e.g., dementia and tremors).

A hallmark of NDDs is the accumulation of toxic protein aggregates in the brain. These aggregates often arise from proteins that, due to mutation, post-translational modification, or aberrant processing, adopt aggregation-prone conformations and gradually accumulate.^326^ In some NDDs, such as traumatic brain injury or spinal cord injury, protein aggregates—like tau—are considered secondary to the disease but may nonetheless contribute to its persistence or progression.^326^ A small subset of NDDs, such as hereditary PD attributed to LRRK2 mutation, are not associated with toxic protein aggregation.^326^ Nevertheless, protein aggregation is considered a primary contributor to disease development in most NDDs, including major classes such as AD and PD, which are covered in more detail in the following sections. In addition, dysproteostasis in a wider sense, including disruption of protein degradation pathways such as the UPS and the autophagy‒lysosome system, has been considered a separate hallmark of NDDs.^326^

#### Alzheimer’s disease

AD is the most prevalent NDD and the leading contributor to dementia, accounting for two-thirds of dementia cases.^485^ AD has highly age-dependent incidence rates, is far more common in older populations, and is also more prevalent in women than in men.^485^ Due to the risk conferred by advanced age and global increases in life expectancy, the incidence of AD is estimated to triple by the year 2050.^485^ AD risk is also increased by certain genetic predispositions, including at least 29 multigene loci,^486^ and both genetic and nongenetic factors contribute to the estimated 60–80% heritability of AD.^485^ AD is characterized by progressive cognitive impairment over its course, typically culminating in severe memory deficits, mood and behavioral changes, aphasia, and motor deficits.^487^

AD is characterized by dysproteostasis at many different levels, most notably through the well-characterized presence of abnormal protein aggregates in AD brains. This is exemplified by recent guidelines on AD research, which have promoted a framework in which the disease is defined exclusively on the basis of biomarkers, such as the ATN system, wherein (A)myloid-β plaques, (T)au neurofibrillary tangles, and (N)eurodegeneration constitute the three classes of biomarkers.^485,488^ In this framework, the use of clinical symptoms or signs in AD diagnosis is de-emphasized or discontinued altogether. This is motivated, for instance, by the observations that 10–30% of patients diagnosed with AD dementia do not exhibit the biomarkers by which AD is defined and, conversely, that these biomarkers are present upon biopsy in a large fraction (30–40%) of asymptomatic (cognitively unimpaired) elderly individuals.^488^ As such, a shift to the biomarker-based ATN framework in AD research may position future studies to more precisely identify AD-specific etiological factors. Measuring the levels of the ATN biomarkers in patients, however, requires a small battery of invasive or costly tests, such as cerebrospinal fluid (CSF) sampling, positron emission tomography (PET) scans with amyloid or tau ligands, and magnetic resonance imaging (MRI) to evaluate neurodegeneration, and is currently not a feasible approach for the everyday clinical diagnosis of AD.^488^ In the clinic, cognitive tests and patient/carer history remain the mainstay methods for identifying AD signs and symptoms (amnesia, disorientation, mood changes, etc.), and additional laboratory tests (full blood count, metabolic panel, etc.) are used to rule out differential diagnoses (e.g., metabolic causes of dementia), often with radiological imaging (MRI/PET) to assess neurodegeneration and rule out vascular dementia.^487^

Protein aggregates in AD primarily come in two flavors: the abovementioned amyloid-β (Aβ) peptides and tau neurofibrillary tangles (NFTs). Aggregation-prone Aβ peptides are derived from amyloid-beta precursor protein (APP) when cleaved by β-secretase and γ-secretase complexes (the amyloidogenic pathway); the latter complex releases a 37–49-residue-long region of the APP transmembrane domain.^489^ The length of the Aβ peptide determines its physicochemical properties and propensity for aggregation. Aβ40 (where 40 describes the peptide’s length) predominates in healthy individuals and does not aggregate readily, whereas Aβ42 includes two extra hydrophobic residues (I41 and A42) and is the main contributor to Aβ aggregation in AD.^489^ Notably, mutations in PSEN1 or PSEN2 (constituting alternative catalytic components of the γ-secretase complex), which lead to increased production of Aβ42, are responsible for large fractions of hereditary AD cases, and APP mutations that influence the interaction of the APP protein with secretase complexes to increase the generation of aggregation-prone Aβ peptides, are also risk-conferring for AD.^489^ The Aβ aggregates themselves form extracellularly after the secretion of Aβ peptides.^489^

As outlined in Section “Errors in co- or post-translational protein modifications”, tau NFT generation may be prefaced by tau hyperphosphorylation, leading to the adoption of an aberrant conformation and dissociation of tau from microtubules and conferring resistance to proteasomal and autophagic degradation.^257–259^ Consequently, tau proteins form beta-sheet-rich paired helical filaments (PHFs) that aggregate into NFTs and accumulate intracellularly. NFT (neurofibrillary tangle) is a term based on the early identification of extensive cytoskeletal polymers (microtubules, intermediate filaments/neurofilaments) in neurons by microscopy, at a time when they cannot be further discerned and are collectively referred to as neurofibrils.^490^ Subsequently, tau aggregates were identified as abnormal “neurofibrils” with a tangled appearance,^491^ and the NFT term has stuck with these tau aggregates till present day. A definitive diagnosis of AD requires the detection of both Aβ aggregates and tau NFTs and is corroborated by coincident neurodegeneration.^488^ Abnormal levels of amyloidogenic Aβ peptides or hyperphosphorylated tau may be detected via positron emission tomography (PET) scans in tandem with radiotracer labeling of the corresponding proteins or through sampling of cerebrospinal fluid (CSF), whereas neurodegeneration is more readily visualized via magnetic resonance imaging (MRI).^326,488^

According to the amyloid hypothesis, AD pathogenesis is initiated by Aβ aggregation, which promotes subsequent progressive tau hyperphosphorylation and tau NFT generation; the latter correlates more strongly with clinical manifestations (e.g., cognitive decline) than does the former.^258,485^ On the basis of these findings, multiple putative etiological mechanisms, such as the indirect or direct modulation of a two-digit number of neuronal cell surface receptors by extracellular Aβ, have been proposed as effects of Aβ and tau buildups.^258,489^ The activation of PRRs on microglia and astroglia by misfolded proteins may promote extensive neuroinflammation and contribute to AD progression.^492^ Yet other mechanisms may contribute to the generation of Aβ and tau pathology or act in parallel. Above, we have introduced several examples, such as metal dyshomeostasis mediated oxidative stress,^290,311^ oxidative stress due to mitochondrial dysfunction,^493^ deficiencies in proteasome- or lysosome-mediated protein degradation,^324,342^ and aging-associated protein deamidation or amino acid epimerization.^353–355,357^ Aβ and tau pathology has also been implicated in the facilitation of neuronal hyperexcitability, possibly contributing to the cognitive dysfunction observed in AD, together with other mechanisms such as calcium dyshomeostasis and dysregulated glutamatergic or GABAergic signaling.^494^ Taken together, evidence suggests that errors at multiple levels of the proteostasis network, many of which may act additively or synergistically, may contribute to AD pathogenesis, highlighting the potential for further research in this field to possibly advance the management of AD and other NDDs.

#### Parkinson’s disease

PD is the second most frequently diagnosed neurodegenerative disorder, and, like AD, its incidence increases with increasing age.^495^ Consequently, PD is also expected to increase in prevalence owing to global trends of increasing life expectancy, but a significant portion of its observed increase in incidence—suggested to be the most rapid among neurodegenerative disorders—cannot be explained by aging population structures.^496^ Other factors, such as increased environmental pollution, have been proposed to provide additional explanations for the observed rise in PD diagnoses.^496^ PD is a progressive disease whose symptoms often present a decade prior to diagnosis and commonly features motor dysfunction (tremors, bradykinesia, instability, rigidity), depression, fatigue, cognitive problems, constipation, and other symptoms.^496^ Motor symptoms are the primary signs employed as diagnostic criteria and, on their own, constitute parkinsonism (also referred to as Parkinson’s syndrome).^497^ A certain PD diagnosis requires post-mortem identification of α-synuclein protein aggregates in intracellular Lewy bodies (which also include some ~300 other protein species) and Lewy neurites in the brain, particularly in the substantia nigra, accompanied by a loss of dopaminergic neurons, which explains the observed motor symptoms.^496,498^ While α-synuclein aggregates in the substantia nigra provide the most compelling link to the disease’s motor manifestations, they first develop in other brain regions (e.g., the olfactory bulb) and gradually develop more extensively throughout the brain.^498^ Notably, evidence suggests that α-synuclein aggregates may spread to other brain regions through exocytosis and endocytosis of proteins in tandem with a prion-like process in which misfolded conformers catalyze the misfolding of normal conformers.^496,499^ The presence of α-synuclein aggregates makes PD a synucleinopathy, along with dementia with Lewy bodies and multiple system atrophy, which are characterized as atypical cases of parkinsonism.^496^

The hallmark presence of α-synuclein aggregates in PD, similar to that of Aβ and tau in AD, implicates dysproteostasis in its etiology, and central questions include how the aggregates form and to what extent they are causative for the disease. Multiple genetic variants of varying penetrance have been linked to PD and are predominantly represented among early-onset (age <50–60 years) cases of the disease. These include mutations in *SNCA* itself, in *PRKN* and *PINK1* (discussed in the context of mitophagy) in early-onset PD, and in *LRRK2* and *DNAJC13* in classic PD.^500^ Many of the genes or loci whose mutations are linked to PD converge on some common pathways, such as vesicular trafficking (*SNCA*, *VAMP4*, *RAB29*), mitochondrial function (*ALAS1*, *PINK1*, *MCCC1*), lysosomal function (*GBA1*, *ATP6V01*, *GALC*), and the proteostasis network (*BAG3*, *DNAJ6*, *PRKN*), possibly pointing toward different routes to similar states of cellular dysfunction.^500^ α-Synuclein aggregates themselves are associated with disruption of ER-to-Golgi trafficking as well as mitochondrial and lysosomal functions, and accordingly, the overall landscape of possible etiological processes in PD includes many theoretical feedback mechanisms in which the different dysfunctional pathways magnify each other’s dysfunction.^500^ For example, lysosomal function depends on vesicular trafficking for the delivery of its enzymes and mitochondrial respiration for ATP to drive transport, acidification, and catabolic processes. PD-linked mutations may interfere with any of these pathways, leading, e.g., to decreased lysosomal degradation of α-synuclein aggregates or increased aggregation of α-synuclein due to oxidative damage downstream of ROS release from defective mitochondria. As such, different kinds of mutations, environmental insults, or age-related alterations in the activity of these central processes may be connected in an ever-regressing circle.^500^

### Metabolic disorders

Metabolic disorder is a broad term that subsumes conditions and diseases characterized by complications or pathology that, in part, or completely, is caused by sustained imbalances in macronutrient intake and/or metabolism or by physiological responses to such sustained imbalances. For instance, obesity is a metabolic disorder characterized by excess body fat due to the maintenance of a prolonged positive energy balance and often has disadvantageous effects on metrics such as blood lipid and blood sugar levels, inflammation, and hormonal signaling.^501^ Type 2 diabetes mellitus (T2D) is another metabolic disorder characterized by insufficient pancreatic insulin production due to a loss of pancreatic β-cells and the consequent dysregulation of glucose and lipid metabolism—a hallmark of persistently elevated blood glucose levels.^502^ Finally, the etiology of the majority of cardiovascular diseases (CVDs)—the largest cause of death globally as of 2021^495^—are intimately linked to multiple other conditions regarded as metabolic disorders (e.g., obesity, T2D, hyperlipidemia),^503^ underscoring their impact on global health. In this section, we explore the alterations and disruptions to proteostasis observed in these disorders, as well as the secondary contributions of stress response pathways to their pathophysiology.

#### Type 2 diabetes

T2D is a condition in which peripheral tissue insulin resistance combined with decreased pancreatic β-cell mass and/or function leads to chronic hyperglycemia.^502^ Multiple other mechanisms—recently referred to as “the deleterious dozen”—have also been characterized as contributing to the development of hyperglycemia.^502^ The associated glucotoxicity, oxidative stress, and other stresses, together with contributions from common comorbidities such as obesity and hypertension, act upon multiple tissues and organs to accelerate metabolic aging, frailty, physical dysfunction, and disability.^504^ Closely following the global progression toward more sedentary lifestyles and unhealthy diets, the incidence of T2D is increasing and poses a formidable challenge to public health.^502^

As with other secretory cell types, proteostasis in β-cells is under constant pressure due to their high rates of protein synthesis and secretion. Specifically, upon elevated blood glucose, β-cells synthesize proinsulin, which is proteolytically processed and secreted as insulin. Studies suggest that insulin production may constitute 10–50% of total β-cell protein synthesis, depending on the level of stimulation.^505^ Proinsulin is inherently prone to misfolding, with an estimated misfolding rate of 20% for the wild-type protein—increasing with certain *INS* gene variants, perturbations to parameters such as pH and Ca^2+^ levels in the ER, or inadequate function in pathways that maintain or restore ER function, including the UPR.^505^ For instance, *Perk*-knockout mice progressively develop a T2D-like phenotype, and in humans, *PERK* loss-of-function mutations are causative for Wolcott‒Rallison syndrome, which involves the onset of diabetes during infancy.^506^ Mice with pancreatic *Ire1* deletion display defects in the glucose-induced expression of genes involved in insulin biosynthesis, leading to decreased insulin secretion and elevated blood glucose.^507^ However, hyperactive IRE1α signaling is also associated with β-cell deficits, and ATF4 and CHOP deletion may have protective effects against β-cell dysfunction or T2D, suggesting that the UPR may also mediate apoptotic signaling in stressed β-cells.^404,508^ Attempts at expressing ^35^S-labeled proinsulin in non-β-cell lines have shown that it excessively aggregates and is subject to large-scale degradation, indicating that β-cells rely on specialized adaptations and a fine-tuned ER environment to handle the requirements for insulin expression and secretion.^509^ Consequently, genetic predispositions or other insults that exacerbate proinsulin aggregation have been proposed as common upstream contributors to the development of T2D.^505^ In line with this, treatments that putatively enhance the protein folding capacity of the ER, such as the chemical chaperones 4-PBA and TUDCA (tauroursodeoxycholic acid), have shown promise in alleviating hyperglycemia and insulin insensitivity in obese and diabetic mice.^510^ In one example, ER stress and β-cell death in the Akita mouse model of diabetes, which carries an *INS* mutation that promotes its misfolding, has been alleviated through autophagy activation via rapamycin-mediated mTOR inhibition.^511^

T2D is also associated with pancreatic aggregation of the islet amyloid polypeptide (IAPP), which is processed by the same proteases as insulin and cosecreted with insulin by β-cells.^512^ Pancreatic IAPP aggregates exhibit β-cell toxicity and correlate with β-cell loss in humans.^512^ This intuitively seems contradictory, as β-cell loss in T2D patients should decrease IAPP production and secretion, possibly suggesting that IAPP aggregates may form at an earlier stage of the disease, where insulin resistance causes still highly functional β-cells to upregulate their secretory activities. Research indicates that IAPP aggregates are amenable to clearance by autophagy and that mice with β-cell-specific *Atg7* knockout are more susceptible to β-cell toxicity and the development of diabetes when they express human IAPP (hIAPP),^513^ whereas hIAPP-expressing mice treated with trehalose exhibit enhanced autophagic activity and improved glucose tolerance.^514^ Other studies have demonstrated that β-cell-specific autophagy deficiency is cytotoxic to mice, suggesting more general contributions to β-cell viability.^515^ Despite the observed significance of an intact autophagy pathway in vitro for human β-cell lines and in vivo for rodent β-cells, autophagy deficiency has not been explicitly established as a contributing etiological factor in T2D itself.^502^

#### Nonalcoholic fatty liver disease

Another metabolic disorder closely linked to obesity is nonalcoholic fatty liver disease (NAFLD), an umbrella term for liver conditions of different severities, ranging from the generally nonprogressive NAFL to progressive and more severe nonalcoholic steatohepatitis (NASH).^516^ NAFLD involves hepatic triglyceride accumulation (steatosis) accompanied by inflammation and liver degeneration and may progress to both more severe liver conditions (cirrhosis, hepatocellular carcinoma) as well as to extrahepatic disease (T2D, cardiovascular disease).^516^ Both the total number of cases of NAFLD, as well as the proportion of severe cases, are increasing in prevalence; a 2016 meta-analysis estimated the global NAFLD prevalence to be ~25%.^517^ Despite this, therapeutic opportunities are currently scarce, although candidates may be emerging in clinical trials.^516,518^

Owing to its role in lipid synthesis, the ER has been at the center of attention in NAFLD, and studies have indicated ways in which pathways regulating ER function (e.g., the UPR) may contribute to NAFLD pathogenesis, as well as characterized disruptions to the proteostasis network that are commonly observed in NAFLD. For instance, ER stress markers are upregulated in the livers of obese subjects but decrease after gastric bypass surgery, correlating with a decrease in liver steatosis^519^; moreover, indicators of increased ER stress are detected in liver biopsies from NAFLD patients.^520^ Steatosis may, e.g., induce ER stress through alterations in ER membrane composition, which has been shown to disrupt the SERCA channel and trigger Ca^2+^ dyshomeostasis.^521^ The hepatic deletion of either *Ire1α* or *Xbp1* in mice leads to dysregulated lipid homeostasis, but in diverging ways, increasing or decreasing liver steatosis, respectively; this could perhaps be due to observations that IRE1 RIDD activity toward certain miRNAs has been characterized as protective against steatosis.^522^ The contributions of the other UPR arms to lipid homeostasis have also been described, including protective effects of both the PERK/eIF2α and ATF6 branches against hepatosteatosis in ER-stressed mice and an ATF6-mediated upregulation of fatty acid oxidation and downregulation of lipogenesis in hepatocytes.^522,523^ Taken together, the dual roles of the ER and UPR in both lipid and protein homeostasis likely enables some level of crosstalk between the two. However, persistent ER stress has also been implicated in driving NAFLD progression through UPR-mediated activation of pro-inflammatory (NF-κB, JNK, and the NLRP3 inflammasome) and apoptotic pathways.^522,523^

In addition to the UPR, the HSR may also contribute to protection against NAFLD progression. A progressive downregulation of HSF1 has been reported under NASH pathogenesis, where HSF1 activation by SYSU-3d was observed to decrease NASH pathology in mice, in part through alleviation of oxidative stress.^524^ This gradual decline in HSF1 and downstream chaperone activity during NAFLD progression, inversely correlated with plasma markers of oxidative stress, has also been demonstrated in obese patients,^525^ and a study applying heat shock and mild electrical stimulation to putatively activate the HSR has shown promise in decreasing NAFLD markers in patients with metabolic syndrome or T2D.^526^

Due to the role of autophagy in lipid metabolism and the regulation of intracellular lipid stores—termed lipophagy—as well as its function in the removal of damaged macromolecules and organelles, studies have also examined the potential involvement of autophagy in NAFLD. Other reviews have described how the overexpression of specific autophagy genes, or their upstream transcriptional regulators, can alleviate steatosis in mouse models of obesity or hyperinsulinemia that exhibit autophagy deficiency.^527^ Conversely, perturbation of autophagy in hepatocytes may exacerbate oxidative damage and proapoptotic signaling.^527^ Liver biopsies from NAFLD patients show signs of impaired autophagic flux,^520^ indicating that observations from cell lines and animal models may also hold up in human patients. In vitro experiments highlight the mTOR pathway as one of the possible mechanisms that may mediate autophagy suppression in hepatocytes upon fatty acid accumulation.^520^ Taken together, there is evidence for the dysregulation of several central proteostasis pathways in NAFLD. Given the promising effects of their manipulation in preclinical models, some of these pathways may represent candidate targets for future therapies aimed at these liver pathologies.

### Cancer

Cancer encompasses diseases characterized by uncontrolled clonal cell proliferation driven by genetic alterations that enable rapid growth and high mutability.^528^ As tumors evolve in harsh environments, they experience significant proteotoxic stress that challenges their protein-folding machinery.^529^ Oncogenic mutations often result in unstable or misfolded proteins, whereas chromosomal abnormalities such as aneuploidy produce imbalances in protein complex subunits, leading to the accumulation of unpaired polypeptides. In this context, increased protein synthesis, especially in cancers driven by oncogenes such as MYC, floods the cell with nascent polypeptides requiring proper folding.^529^

These internal stressors are further intensified by the tumor microenvironment (TME). Dysregulated metabolism, oxidative stress, hypoxia, and nutrient deprivation not only promote protein damage and misfolding but also exacerbate the burden on folding systems.^529^ Collectively, these conditions create a state of chronic proteotoxic stress in cancer cells.

To survive these challenges, cancer cells activate stress response pathways—including the UPR, autophagy, and the HSR—which together increase folding capacity, reduce protein load, and sustain cell viability.^529,530^ These pathways are not merely protective; their successful engagement supports continued proliferation, apoptosis evasion, immune escape, and therapy resistance.^529^ In contrast, failure to maintain proteostasis results in growth arrest or cell death. This divergence presents potential therapeutic vulnerability, as it may allow the selective targeting of cancer cells by disrupting their reliance on proteostasis mechanisms.

Central to this adaptive machinery are molecular chaperones, particularly HSPs, which are markedly upregulated in many tumor types and frequently correlate with poor prognosis and resistance to therapy.^531^ Among them, HSP90 is especially critical, stabilizing a broad range of oncogenic clients—including kinases, transcription factors, and ubiquitin ligases.^107^ Critical oncogenic signaling proteins such as EGFR, HER2, AKT, and BCR-ABL rely on HSP90 for stability and function.^107,109,532^ Additionally, mutant TP53 proteins, which frequently exhibit dominant-negative or gain-of-function mutations, are safeguarded by HSP90, preventing proteasomal degradation and sustaining tumor progression.^107^

In addition to stabilizing oncoproteins, molecular chaperones also actively suppress apoptosis. For example, HSP70 sequesters key proapoptotic factors such as APAF-1 and AIF, thereby increasing the threshold for apoptosis induction.^533–535^ Other chaperones, such as HSP27 and associated co-chaperones further enhance cell survival, and disrupting these interactions can restore the sensitivity of cancer cells to apoptosis.^535^

A major site of proteostasis disruption in cancer is the ER, where high translational activity and secretory demands can easily overwhelm folding capacity. Oncogenes such as MYC, RAS, BRAF, and HER2 stimulate excessive protein synthesis, quickly saturating the folding capacity of the ER.^529,536^ For example, c-MYC overexpression in MEFs activates UPR signaling, which can be reversed by treatment with chemical chaperones such as 4-phenylbutyric acid.^537^ In the Eμ-Myc mouse model, the UPR is activated in lymphocytes and is dependent on elevated protein synthesis, as reducing the translational load (e.g., via L24 haploinsufficiency) prevents UPR induction.

Cancers with high secretory activity—such as multiple myeloma, which produces high levels of immunoglobulins, or mucin-secreting lung, breast, and colon tumors—place continuous folding demands on the ER, potentially increasing their reliance on the UPR.^530^ The TME further induces ER stress. As tumors outgrow their blood supply, they develop hypoxic and nutrient-deprived regions.^530,538^ Hypoxia impairs oxidative protein folding due to limited oxygen availability for disulfide bond formation,^539^ whereas glucose deprivation impairs glycosylation and calcium homeostasis, which are essential for ER function.^538,540^ These stressors exacerbate protein misfolding, activating UPR signaling, particularly in hypoxic tumor regions.^541^

To adapt, cancer cells exploit three branches of the UPR—IRE1, PERK, and ATF6—which collectively reduce the ER burden, increase folding and degradation capacity, and sustain survival under stress.^538^ However, in cancer, these pathways often hijack to support not only proteostasis but also oncogenic growth, metabolic reprogramming, immune evasion, and drug resistance.^530,542^ Below, we examine how each arm of the UPR is rewired in cancer to reinforce malignant progression.

#### PERK signaling in cancer

The PERK–ATF4 arm of the UPR plays a critical role in maintaining protein homeostasis in cancer cells under proteotoxic stress.^530,537,543–546^ By balancing global protein synthesis and coordinating adaptive transcriptional programs, PERK signaling supports cell survival, metabolic adaptation, and resistance to stress.

Recent studies have indicated that ATF4 and the oncoprotein MYC co-occupy the promoter regions of more than 30 MYC target genes to regulate metabolic pathways and protein synthesis, establishing the cellular homeostasis necessary for rapid cell growth.^543^ Among these proteins is 4E-binding protein 1 (4E-BP1), a negative regulator of translation that buffers the proteome against MYC-induced misfolding. Experimental ablation of PERK or eIF2α phosphorylation in MYC-expressing cells triggers apoptosis and abrogates tumorigenesis in vivo,^537^ highlighting the role of PERK as a gatekeeper of proteostasis in rapidly proliferating cancer cells.

PERK–ATF4 signaling also promotes metabolic rewiring, notably through the upregulation of MTHFD2, a key enzyme in mitochondrial one-carbon metabolism.^544^ MTHFD2 levels are significantly increased in many cancer types, and its silencing significantly impairs prostate cancer (PCa) tumor development in preclinical models, which is consistent with its overexpression in human PCa.^544^ Under stress conditions, when MYC levels are downregulated due to the inhibition of protein translation, ATF4 compensates by inducing MTHFD2 expression, ensuring continued support for the m1C cycle and maintaining cellular metabolic homeostasis. Additionally, PERK promotes autophagy, both as a mechanism for ER quality control and as a source of metabolic substrates.^537,547^ ATF4 and its downstream target CHOP promote the expression of autophagy genes such as ATG5, ATG12, and BECN1, facilitating the clearance of unfolded proteins when the proteasome is overwhelmed.^548^ Pharmacological autophagy inhibition sensitizes tumors to hypoxia and irradiation, whereas PERK–ATF4-mediated autophagy promotes resistance to anoikis in invasive cancer cells by limiting ROS accumulation.^545–547^ This was suggested as a mechanism of reduced lung metastasis of fibrosarcoma cells upon ATF4 knockdown.^545^

PERK signaling supports antioxidant defense mechanisms by activating NRF2 through phosphorylation, leading to its release from KEAP1 and nuclear translocation to induce the expression of ROS-detoxifying genes, aiding cancer cell survival under oxidative stress.^453,542^ Nrf2-knockout MEF cells display significantly increased sensitivity to tunicamycin.^453^ PERK also promotes antioxidant defense via ATF4, which induces NRF2 and genes for GSH synthesis, thereby reducing ROS levels. Paradoxically, a recent study reported that ATF4 also upregulates the expression of CHAC1, a GSH-degrading enzyme that reduces GSH levels.^454^ CHAC1 knockdown led to a strong increase in GSH levels and fully prevented NRF2 stabilization upon ATF4 induction. Measurement of intracellular or extracellular GSH, cystine and glutamate levels indicated that cells deplete GSH to increase cysteine availability. This raises the question as to why cells need extra cysteine under stress conditions. Upon ATF4 induction, the increased expression of the xCT antiporter system, which imports extracellular cystine in exchange for intracellular glutamate, is considered a cellular mechanism to induce GSH synthesis to detoxify ROS. However, the reduced levels of GSH may indicate that cells may need cysteine for another purpose.

ATF4-mediated induction of the cysteine-rich protein CRELD2 may help explain the increased cysteine demand observed during stress.^549^ While cysteine is one of the least abundant amino acids—comprising less than 2% of residues in most proteins—CRELD2, which localizes to the ER and Golgi, is exceptionally enriched in cysteine (10.8%) and contributes to ER homeostasis by interacting with chaperones and stress-related enzymes.^549^ In skeletal cells, CRELD2 functions as a chaperone for the LRP1 protein and has also been reported to regulate ER calcium release.^549^ According to the genome-wide Perturb-seq data analyzed by GeneSetR (our unpublished data), CRELD2 expression is markedly upregulated upon the knockdown of BiP, or DHDDS, an enzyme essential for N-linked protein glycosylation.^550^ Studies in CRELD2-deficient mice further suggest that the protein enhances ER stress tolerance and protein folding capacity.^551^ Together, these findings support the idea that PERK/ATF4-driven cysteine accumulation may promote the synthesis of CRELD2, a critical ER chaperone, under stress conditions.

In addition to its role in proteostasis, CRELD2 has been implicated in cancer metastasis and immunomodulation. It is linked to tumor progression in several cancers and enhances endothelial cell invasiveness.^552–554^ By facilitating proper protein folding during ER stress, PERK/ATF4 signaling may indirectly support CRELD2-driven metastatic potential. Additionally, CRELD2 plays a key role in reprogramming the TME by promoting the transformation of fibroblasts into cancer-associated fibroblasts (CAFs), thereby fostering a tumor-supportive stroma.^553,554^ In breast and cutaneous squamous cell carcinoma, ROCK signaling induces CRELD2 expression and secretion via the PERK/ATF4 axis. Conditioned medium from ROCK-activated tumor cells elevates CRELD2 levels, which in turn enhances fibroblast motility and transforms their phenotype to a tumor-promoting state. In contrast, fibroblasts exposed to media lacking CRELD2 exhibit reduced motility, whereas recombinant CRELD2 restores motility, highlighting its role as a paracrine driver of CAF activity. In vivo coengrafting of ROCK-educated fibroblasts with tumor cells accelerates tumor growth, demonstrating that CRELD2 is both necessary and sufficient to drive a tumor-supportive fibroblast phenotype. Thus, ATF4/CRELD2 signaling not only supports cancer cell survival under stress but also reprograms the surrounding stroma to foster tumor growth and progression.

PERK/ATF4 signaling promotes cancer metastasis through multiple mechanisms, particularly by supporting epithelial-to-mesenchymal transition (EMT), a process that enhances cancer cell invasion and motility.^555^ In cells that have undergone EMT, PERK/ATF4 signaling is constitutively activated, likely to manage the increased secretory load resulting from elevated ECM production, which promotes cell motility and invasion.^555^ Primary breast cancer (BCa) cells with EMT markers exhibit increased PERK levels, increased eIF2α phosphorylation, and ATF4 gene signatures correlated with EMT programs across various tumors.^555^ PERK inhibition reduces invasion and tumorsphere formation, highlighting its role in metastasis.^555^ Additionally, ATF4 promotes EMT by upregulating the expression of transcription factors such as Slug and enhancing angiogenesis via VEGF.^556,557^

In addition to its direct role in cancer cells, PERK/ATF4 signaling contributes to tumor progression through immunomodulation. In myeloid-derived suppressor cells (MDSCs), ATF4 activation promotes an immunosuppressive phenotype.^558^ PERK deletion impairs NRF2-dependent antioxidant defense, disrupts mitochondrial homeostasis, and activates STING-dependent type I interferon signaling, reducing MDSC-mediated T-cell suppression and slowing tumor growth. In vitro, PERK deletion in MDSCs significantly reduced their ability to suppress CD8 + T-cell proliferation, whereas in vivo, it markedly delayed tumor growth, highlighting the critical role of ATF4 in maintaining immunosuppressive MDSCs. Additionally, ATF4-mediated CHOP expression in MDSCs enhances immune suppression by driving interleukin-6 (IL-6) production through CCAAT/enhancer-binding protein-β (C/EBPβ) signaling.^559^

PERK-ATF4-C/EBPβ signaling is also critical for reprogramming hematopoietic stem/progenitor cells (HSPCs) into MDSC precursors in the spleen.^560^ Given the toxicity of systemic PERK inhibition, targeted delivery of PERK inhibitors to the spleen was tested in hepatoma and lung carcinoma models. Splenic administration of PERK inhibitors (GSK2606414, AMG PERK 44) reduces tumor growth more effectively than direct tumor targeting does, suggesting that localized PERK inhibition may enhance antitumor immunity while minimizing toxicity.^560,561^

PERK/ATF4 signaling also regulates macrophage polarization within the TME. In bone marrow-derived macrophages, PERK activity increases during differentiation into the immunosuppressive M2 phenotype, supporting mitochondrial function through serine biosynthesis and α-ketoglutarate production.^562^ Macrophages from Psat1-deficient mice (an Atf4 target gene) show reduced M2 polarization and impaired T-cell suppression. In vivo, Psat1 deletion in myeloid cells reduces melanoma tumor growth, highlighting a role for ATF4/PSAT1 signaling in the immunosuppressive functions of M2 macrophages.^562^

PERK inhibition may also enhance antitumor immunity by inducing immunogenic cell death (ICD). In melanoma models, PERK deletion or pharmacological inhibition triggers SEC61β-mediated paraptosis in ER-stressed cancer cells, leading to ICD and the activation of type I interferon responses in dendritic cells (DCs).^563^ These activated DCs promote T-cell recruitment and activation, driving robust antitumor immunity. PERK inhibition delays tumor growth in a T-cell-dependent manner and enhances the efficacy of immune checkpoint blockade therapies such as anti-PD-1.^563^

Additionally, PERK/ATF4 signaling modulates CD8^+^ T-cell survival and function.^564,565^ Under stress, PERK-ATF4-CHOP-Bim activation induces CD8^+^ T-cell death, which can be rescued by PERK inhibition.^564^ Taurine deficiency in CD ^+^ T cells also triggers exhaustion through PERK/ATF4 signaling, a process exacerbated in the TME, where tumor cells outcompete T cells for taurine uptake.^565^ Therefore, targeting PERK may enhance CD8^+^ T-cell viability and antitumor function.

Collectively, these findings highlight the multifaceted role of PERK/ATF4 signaling in affecting proteostasis in cancer metastasis and immune regulation. By controlling factors such as CRELD2 and modulating immune cell function, PERK/ATF4 drives tumor progression through both cancer cell-intrinsic and microenvironmental mechanisms. Targeting this pathway offers promising therapeutic strategies to limit metastasis and improve immunotherapy outcomes.

#### IRE1α signaling in cancer

The IRE1α-XBP1 signaling axis is increasingly recognized as a critical regulator of tumor progression and immune modulation across multiple cancer types, including triple-negative breast cancer (TNBC), PCa, and non-small cell lung cancer (NSCLC). Elevated IRE1α activity has been observed in these malignancies, and its inhibition—either genetically or pharmacologically—has been shown to suppress tumor growth in preclinical models.^566–568^ For example, shRNA-mediated knockdown of XBP1 in fibrosarcoma cells or expression of dominant-negative IRE1α in glioma cells significantly inhibited tumor growth and angiogenesis. Notably, the overexpression of XBP1s in fibrosarcoma cells with dominant-negative IRE1α expression rescued these effects, confirming the functional importance of this pathway in tumor biology.^569,570^

In addition to its role in supporting tumor cell growth, IRE1α signaling profoundly influences the tumor immune microenvironment.^566^ In PCa, androgen receptor signaling activates IRE1α-XBP1s to promote c-MYC-driven progression.^567,571^ In syngeneic mouse models, IRE1α loss impaired tumor growth in an XBP1s-dependent manner and remodeled the TME by reducing the number of immunosuppressive TAMs and Tregs while increasing the number of effector T and NK cells.^566^ Mechanistically, IRE1α inhibition enhances interferon-γ signaling and downregulates tumor-promoting pathways such as mTORC1 and glycolysis. Consistently, the IRE1α RNase inhibitor MKC8866 sensitized PCa tumors to anti-PD-1 therapy. Similar effects were observed in NSCLC, where IRE1α loss delayed tumor growth and boosted effector T-cell infiltration while reducing Tregs. This effect was reversed by XBP1s overexpression. Tumor-derived prostaglandin E2 (PGE2), which is reduced upon IRE1α loss, was identified as a key modulator of immune suppression.^572^

In ovarian cancer, tumor-derived factors activate IRE1α-XBP1 in T cells, impairing mitochondrial function and IFN-γ production.^573^ Patient T cells showed increased XBP1 splicing, which was correlated with reduced infiltration and antitumor activity. XBP1-deficient T cells in mice enhance antitumor immunity, suggesting their therapeutic potential in targeting this pathway.

The IRE1α-XBP1s signaling pathway is also important for NK cell proliferation and immune responses against viral infections and tumors.^574^ NK cells lacking IRE1α or XBP1 exhibited significantly compromised proliferation and expansion. Pharmacological blockade of IRE1α resulted in reduced oxidative phosphorylation and mitochondrial respiration, which are critical for NK cell function.^574^ Moreover, similar to findings in PCa cells, c-MYC was identified as a direct target of XBP1s, which contributes to NK cell proliferation.^567^ This mechanism was further validated in vivo via an intravenous B16 melanoma model, in which IRE1α-deficient NK cells failed to control lung tumor colonization, resulting in fewer infiltrating NK cells in the lungs and reduced expression of c-MYC and the proliferation marker Ki-67.^574^

Another recent study explored how tumor-derived lipids promote protumorigenic polarization in macrophages through the activation of the IRE1-XBP1s pathway.^575^ Conditioned medium (CM) from melanoma cells significantly increased lipid accumulation and the expression of protumorigenic markers, such as arginase 1 (ARG1) and mannose receptor C-type I (MRC1), in bone marrow-derived macrophages (BMDMs). Interestingly, CM treatment induced the splicing of XBP1 without significantly activating the PERK pathway or stimulating the expression of ATF6 targets. STF083010, an IRE1α inhibitor, suppressed these protumorigenic changes.^575^ In vivo, myeloid-specific XBP1 deletion reduced melanoma tumor growth and TAM numbers. The macrophage-inducible calcium-dependent lectin receptor (MINCLE) is induced by lipid signals from tumor cells, which facilitate the activation of the IRE1-XBP1s pathway in macrophages.^575^ CM from tumor cells stimulated MINCLE expression, and its blockade impaired CM-induced protumorigenic responses in BMDMs. Notably, the lipid β-glucosylceramide was identified as a key bioactive component driving these effects. Genetic and pharmacological approaches have confirmed that targeting IRE1-XBP1s signaling and lipid metabolism could effectively diminish the protumorigenic polarization of TAMs and inhibit tumor progression, presenting promising therapeutic strategies for cancer treatment.^575^

XBP1 is also overexpressed in basal-like TNBC cell lines compared with luminal subtypes.^576^ In orthotopic xenografts, inducible XBP1 knockdown in MDA-MB-231 cells reduced tumor growth, metastasis, angiogenesis, and the CD44⁺CD24⁻ cancer stem-like population. Doxorubicin combined with XBP1 knockdown fully suppressed tumor growth and stemness. Patient-derived CD44^high^/CD24^low^ cells also presented increased XBP1 splicing and UPR marker expression. This study also revealed a significant correlation between XBP1s binding and HIF1α motifs, indicating that XBP1s may play a role in hypoxia adaptation and stem cell maintenance.

Together with these findings on the basis of activation of the XBP1s pathway, a recent study suggested that IRE1α plays a significant role in modulating the immune response to taxane chemotherapy in TNBC through the regulated IRE1α-dependent decay (RIDD) pathway.^577^ RIDD degrades double-stranded RNA (dsRNA) generated by docetaxel, limiting inflammasome activation. MKC8866 plus docetaxel triggered dsRNA accumulation, NLRP3 activation, and pyroptotic cell death, with major changes in the TME. While docetaxel alone resulted in minimal TIL and CD8^+^ T-cell infiltration, combination treatment led to substantial increases in these immune populations in tumors. Global gene expression analyses revealed that IRE1α target genes were negatively correlated with cytotoxic T lymphocyte signatures in BCa patients treated with neoadjuvant taxane-based therapy.^577^ scRNA-seq analysis indicated that combination therapy altered the TME by significantly increasing the number of MHC-II-positive macrophages and dendritic cells, which participate in T-cell priming. Furthermore, the data showed that the therapy not only enhanced cytotoxic T-cell activation but also successfully upregulated PD-L1 expression in tumors, increasing their responsiveness to PD-1 inhibitors. Importantly, restoring the IRE1α pathway through genetic means successfully prevented the immunostimulatory effects of docetaxel, indicating its critical role in immune modulation. Overall, these findings suggest that targeting IRE1α could be a viable strategy to overcome immune evasion in immune-cold tumors and enhance the efficacy of chemotherapy in TNBC.

In addition to these significant effects on the TME, the inhibition of IRE1α signaling also suppresses TNBC cell growth.^578^ MKC8866 reduces the proliferation and secretion of protumorigenic cytokines (IL-6, IL-8, CXCL1, GM-CSF, and TGFβ2) and enhances paclitaxel efficacy in mouse models.^578^ Notably, the combination of MKC8866 with paclitaxel resulted in significantly enhanced tumor growth suppression and increased survival in mouse models while also reducing mammosphere formation post-chemotherapy, a surrogate marker for cancer stem cell properties, suggesting that the maintenance of IRE1α inhibition could prevent tumor reemergence after chemotherapy cessation. Consistently, high IRE1α activity is correlated with inflammation and poor prognosis in basal-like TNBC.^578^

IRE1α activity has also been shown to modulate antigen presentation in dendritic cells (DCs), thereby influencing CD8⁺ T-cell responses.^579^ In DCs, antigen-derived hydrophobic peptides activate IRE1α, which triggers RIDD-mediated degradation of MHC class I heavy chains, reducing their capacity to present antigens to T cells. Interestingly, knockout of IRE1α in CT26 colon cancer cells had a minimal effect on tumor growth in syngeneic mice, whereas systemic pharmacological inhibition of IRE1α significantly suppressed tumor progression. Treated animals presented elevated surface MHC-I on DCs and increased infiltration of cytotoxic CD8⁺ T cells in the TME. In contrast, IRE1α knockout in 4T1 TNBC cells reduced tumor size by ~50%, whereas systemic IRE1α inhibition led to an ~80% reduction.^579^ scRNA-seq analysis confirmed the increased abundance of both DCs and CD8⁺ T effector cells in treated tumors. In the orthotopic EMT6 TNBC model, IRE1α deletion alone had little impact on tumor growth, mirroring the CT26 model. However, combining IRE1α inhibition with anti-PD-L1 treatment resulted in strong tumor suppression. These findings suggest that targeting IRE1α can enhance DC-mediated T-cell priming and recruitment, ultimately strengthening antitumor immunity. In contrast, another study reported that IRE1α overexpression in CT26 colorectal and Lewis lung carcinoma cells limited tumor growth.^580^ IRE1α overexpression induced XBP1 splicing and RIDD activation. In immunocompetent, but not immunodeficient, mice, tumor growth was reduced, accompanied by increased CD3⁺, CD4⁺, and CD8⁺ T cells. Further studies in different model systems are warranted to evaluate the effects of IRE1α overexpression on tumor growth.

Collectively, these findings reveal that IRE1α acts as a potent modulator of both biology of the cancer cell and immune cell dynamics within the TME. Thus, targeting IRE1α and its associated pathways may represent a therapeutic strategy to reprogram the TME, enhancing the effectiveness of existing immunotherapies. Future efforts should be directed toward elucidating the specific immune mechanisms by which IRE1α influences TME composition and function, with the potential to inform novel therapeutic interventions in cancer treatment.

#### ATF6 signaling in cancer

The ATF6 arm is the least characterized among the canonical ER stress pathways in terms of cancer; nevertheless, several studies have suggested that it also contributes to various aspects of tumor progression, chemoresistance, and prognosis. Similar to the other two arms of the UPR, ATF6 transcriptional activity is also elevated across various cancer types, including colorectal cancer, lung cancer, PCa, and BCa, albeit to different degrees.^581,582^ In colon cancer, ATF6 directly upregulates the expression of CIP2A, an oncogene associated with poor prognosis.^581^ Another study in colon cancer explored the role of ATF6 in protecting cells from DNA damage by inducing BRCA1 through mTOR signaling.^583^ A very recent study reported that ATF6 facilitates colorectal cancer growth by promoting a cancer stem cell-like phenotype through Wnt signaling.^584^ Genetic or biochemical inhibition of ATF6 activity significantly halted the cell cycle and decreased the viability of colorectal cancer cell lines. Moreover, Dox-induced ATF6 knockdown in Colo201 tumor xenografts resulted in an over 70% reduction in tumor volume.

ATF6 signaling was also suggested to be a link between alcohol consumption and the progression of PCa.^585^ Golgi fragmentation in PCa patient tumors was positively correlated with the rate of alcohol consumption; in addition, ethanol (EtOH) induced Golgi fragmentation, which led to the translocation of S1P and S2P proteases from the Golgi to the ER, leading to the activation of ATF6 signaling. ATF6 knockdown suppressed EtOH-induced increases in colony formation and trans-well migration. The growth of xenografted LNCaP tumors was enhanced in EtOH-fed animals, and Golgi fragmentation and ATF6 translocation to the nucleus were detected in these tumors.^585^

In melanoma, ATF6 is upregulated upon PTEN deletion; this upregulation is linked to ENTPD5 expression, which has been suggested to play an important role in promoting melanoma cell proliferation, motility, and metastasis through IGF1R signaling.^586^ Finally, in gastrointestinal stromal tumors (GISTs), ATF6 expression was observed in 67% of patient samples and was correlated with shorter relapse-free survival.^587^ GISTs are often characterized by mutations in the KIT protein, resulting in the production of a mutant form (MT-KIT) that accumulates in the Golgi complex. It has been reported that ATF6 contributes to GIST development by mediating HSP90 expression, which enhances the folding and stability of MT-KIT. In addition, pharmacological or genetic inhibition of ATF6 reduced GIST cell viability both in vitro and in vivo, irrespective of imatinib resistance. In summary, these data suggest that ATF6 signaling may be involved in promoting different cancer types.

### Other diseases

Beyond the aforementioned diseases, several other disorders arise directly from protein misfolding, highlighting the centrality of proteostasis to human health. Lysosomal storage disorders such as Fabry disease, Gaucher disease, and Niemann–Pick type C (NPC) exemplify how misfolding disrupts critical degradation pathways. In Fabry disease, mutations in the GLA gene destabilize α-galactosidase A, leading to misfolding, ER retention, and degradation via ER-associated pathways, thereby preventing lysosomal trafficking and resulting in glycosphingolipid accumulation and multiorgan dysfunction.^588^ In Gaucher disease, mutations impair the folding of glucocerebrosidase, similarly triggering ER-associated degradation and accumulation of glucosylceramide within macrophages.^589^ NPC, which is caused predominantly by mutations in the NPC1 protein, results in destabilization and degradation of a lysosomal cholesterol transporter, culminating in lipid accumulation and secondary neuronal dysfunction.^590^ These examples illustrate how protein misfolding within the secretory pathway compromises lysosomal function, organelle homeostasis, and cellular viability, as previously described for membrane proteins such as CFTR in cystic fibrosis (see Section “Errors in the protein synthesis pathway”).

Other systemic protein misfolding disorders, such as transthyretin (TTR) amyloidosis, further demonstrate how destabilization of otherwise soluble proteins can lead to pathogenic aggregation.^591,592^ In TTR amyloidosis, tetramer destabilization facilitates monomer dissociation and partial unfolding, triggering the formation of β-sheet–rich amyloid fibrils, which are deposited extracellularly and disrupt tissue architecture.^591,592^ Interestingly, the pathological shift arises not from loss of native function but from a toxic gain of function through aggregation, illustrating that even minor perturbations in protein stability can drive systemic disease.

Prion diseases, including sporadic and familial forms of Creutzfeldt–Jakob disease, provide a striking example of pathogenic protein misfolding propagation.^593^ In these disorders, the normal cellular prion protein (PrP^C^), which is rich in α-helices, misfolds into a β-sheet–rich pathogenic isoform (PrP^Sc^), which acts as a seed to induce misfolding of additional PrP molecules. This autocatalytic mechanism, which is unique among human diseases, results in widespread neurodegeneration, spongiform changes, and rapid clinical deterioration, underscoring how protein misfolding can drive both cellular dysfunction and protein-only infectious transmission.

Frontotemporal dementia (FTD) and multiple system atrophy (MSA) are additional misfolding-related diseases. In FTD, abnormal aggregation of tau or TDP-43 disrupts microtubule dynamics or RNA processing, respectively, leading to neuronal vulnerability and degeneration.^594^ In MSA, aberrant accumulation of α-synuclein in oligodendrocytes, rather than neurons, underscores the diversity of misfolding targets and cellular contexts, with glial dysfunction contributing to multisystem neurodegeneration.^595^

Collectively, these disorders demonstrate that protein misfolding is central in a wide range of diseases, from single-enzyme deficiencies to systemic fibrillopathies and progressive neurodegenerative syndromes. Despite variations in the identity of the misfolded proteins, the affected cell types, and the clinical manifestations, a unifying principle emerges: disruption of proteostasis through misfolding and aggregation is a fundamental driver of pathology across diverse cell types and tissues.

## Therapeutic strategies and progress in protein misfolding and dysproteostasis-related diseases

**Fig. 7:**
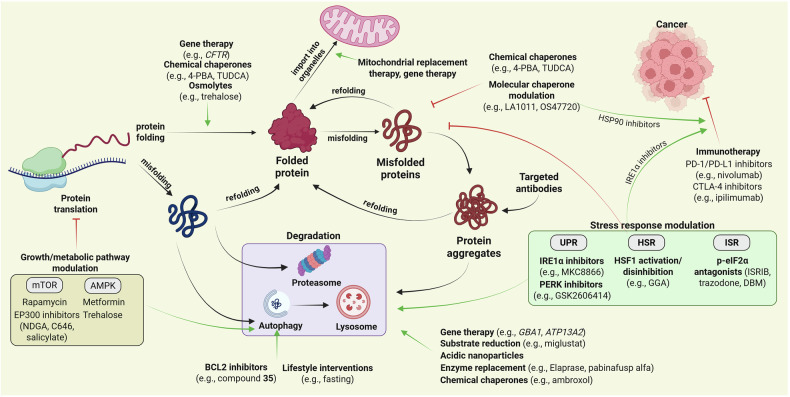
Therapeutic strategies against protein folding and abnormalities in proteostasis. Different therapeutic strategies are aimed at correcting protein folding defects and restoring cellular proteostasis. Small molecules, such as chemical chaperones and molecular chaperone modulators, assist in refolding misfolded proteins, preventing aggregation. Osmolytes and gene therapy can support proper protein conformation and cellular function. Misfolded proteins that fail to refold are targeted for degradation through the proteasome, autophagy, and lysosomal pathways, whose functions may be enhanced or restored by various means, including both pharmacological and lifestyle interventions. Stress response pathways represent another category of therapeutic targets. UPR inhibitors modulate the UPR, while ISR inhibitors target eIF2α phosphorylation. Additionally, HSF1 activators enhance the HSR to boost chaperone function. Metabolic regulators such as mTOR inhibitors and AMPK activators influence protein homeostasis by modulating translation and degradation pathways. In cancer, targeting protein homeostasis is also a therapeutic strategy, where HSP90 and IRE1α inhibitors are being explored alongside immune checkpoint inhibitors. Collectively, these pharmacological approaches aim to restore proteostasis and mitigate diseases associated with protein misfolding and aggregation

As described above, complex challenges are posed by protein folding disorders and dysproteostasis-related diseases. A variety of strategies have emerged to restore protein folding and homeostasis and mitigate disease progression (Fig. 7). Here we explore these therapeutic approaches, focusing first on strategies that directly influence protein folding and aggregation. We discuss methods that enhance molecular chaperone function, promote protein disaggregation or inhibit aggregation, and modulate PTMs to stabilize proteins. Subsequently, we examine interventions targeting cellular stress responses, including the modulation of the HSR, UPR, ISR, and the enhancement of autophagy and the UPS. By addressing both the molecular and the cellular aspects of proteostasis, these strategies offer promising avenues for the treatment of disorders associated with protein folding and dysproteostasis.

### Strategies targeting protein folding and aggregation

#### Molecular chaperone enhancement

Pharmacological strategies targeting protein folding pathways represent promising therapeutic approaches to alleviate the toxic consequences of protein misfolding and aggregation across a range of diseases. Small molecules known as chemical chaperones have attracted increasing attention in this context.^596^ These compounds promote proper folding, prevent aggregation, and stabilize protein conformations through diverse mechanisms, operating independently of the ATP-dependent cycles characteristic of molecular chaperones such as HSPs.

Chemical chaperones can be broadly categorized into two mechanistic classes: osmolytes and hydrophobic compounds.^596^ Osmolytes, such as trimethylamine N-oxide (TMAO), trehalose, betaine, and glycerol, are naturally occurring small molecules that promote protein stability by modifying solvent properties and shifting the folding equilibrium toward the native state.^597,598^ These compounds are widely utilized by extremophiles and stress-adapted organisms to maintain protein function under harsh environmental conditions and have shown potential in therapeutic and biotechnological applications.

In contrast, hydrophobic chemical chaperones act by transiently shielding exposed hydrophobic patches on misfolded or partially folded proteins, thereby reducing aggregation and facilitating proper folding or trafficking, particularly within the ER.^596^ One of the best-characterized hydrophobic chemical chaperones is 4-phenylbutyrate (4-PBA), which helps stabilize protein conformation and improves the folding capacity of the ER, thereby reducing the accumulation of misfolded proteins in the ER.^510^ Administration of PBA led to significant improvements in proteostasis in aged mice, resulting in consolidated sleep patterns and enhanced cognitive performance.^599^ Specifically, PBA treatment reduced the expression of markers of ER stress in the cortex and hippocampus, such as phosphorylated PERK and ATF4, while increasing the expression of the molecular chaperone GRP78. Additionally, PBA enhanced CREB activation, a transcription factor associated with memory formation and synaptic plasticity, thereby improving learning outcomes in hippocampus-dependent tasks.

In addition to its effects on aging, PBA has been found to be effective in many disease contexts characterized by protein misfolding and ER stress.^600^ Despite the large number of studies on its potential, translating these effects into robust clinical outcomes has proven challenging. Although it is an FDA-approved drug for the treatment of urea cycle disorders and has a well-known safety profile, only a few clinical trials have been conducted to investigate its potential for treating neurodegenerative diseases.^601^

TUDCA has been well studied in the context of several protein folding-associated diseases.^602^ Multiple preclinical studies indicate that it exerts neuroprotective effects in animal models of neurodegenerative diseases such as AD, PD, HD, and ALS.^602^ The combination of PBA and TUDCA has been investigated for the treatment of ALS. In the phase II CENTAUR trial, this combination significantly slowed functional decline in patients.^601,603^ These promising results led to the conditional approval of the PBA-TUDCA combination for the treatment of ALS.^604^ However, recent developments have tempered this initial optimism.^605^ A subsequent phase III clinical trial, the PHOENIX trial, failed to demonstrate a significant difference between the treatment and placebo groups, leading to the withdrawal of the combination from the market.^605^ This outcome underscores the challenges associated with translating promising preclinical and early clinical findings into effective treatments for complex neurodegenerative diseases such as ALS.

Migalastat is another FDA-approved chemical chaperone that exemplifies the therapeutic potential of pharmacological chaperones.^606^ Specifically, approved for Fabry disease, migalastat functions by selectively binding to and stabilizing certain mutant forms of α-galactosidase A (α-Gal A) that retain residual activity.^607^ Fabry disease is a lysosomal storage disorder resulting from mutations in the GLA gene, leading to deficient α-Gal A activity and subsequent accumulation of globotriaosylceramide (Gb3) in various organs.^607^ By acting as a chemical chaperone, migalastat promotes the proper folding of these mutant enzymes, facilitates their trafficking to the lysosome, and restores enzymatic activity, thereby reducing substrate accumulation and ameliorating disease symptoms.^607^

The combination therapy of *elexacaftor*, *tezacaftor*, and *ivacaftor* (Trikafta) represents another highly successful chemical chaperone-based approach approved for the treatment of cystic fibrosis (CF). As described previously in Section “Errors in the protein synthesis pathway”, CF is caused by mutations in the CFTR gene, leading to the degradation of misfolded CFTR proteins before they reach the cell surface. Elexacaftor and tezacaftor function as folding correctors, enhancing the proper folding and trafficking of the CFTR protein, whereas ivacaftor potentiates channel opening.^608^ By combining these agents, Trikafta addresses both the folding defect and the gating dysfunction of CFTR, leading to markedly enhanced lung function, reduced pulmonary exacerbations, and improved quality of life in patients with at least one F508del mutation, accounting for the majority of CF cases. Indeed, after its approval, this drug combination led to over 80% decrease in lung transplantation in CF patients.^609^

Ambroxol is another chemical chaperone with demonstrated efficacy in lysosomal storage disorders such as Gaucher disease, where it stabilizes β-glucocerebrosidase (GCase), enhancing enzymatic activity and lysosomal trafficking.^610,611^ Ambroxol has garnered interest for its potential therapeutic role in PD.^610,611^ The ability of ambroxol to increase GCase activity in neuronal cells reduces α-synuclein aggregation, thereby exerting neuroprotective effects.^612^ Clinical trials are currently underway to evaluate the safety and efficacy of ambroxol in patients with PD and Lewy body dementia.^613^ Current findings suggest that ambroxol is well tolerated and can cross the blood‒brain barrier, leading to increased GCase activity in the central nervous system.^611^

Tafamidis is another pharmacological chaperone that has gained approval for clinical use, specifically in the treatment of TTR amyloidosis.^591,592^ Tafamidis works by binding selectively to the thyroxine-binding sites of the TTR tetramer, stabilizing it and preventing its dissociation into monomers—the critical first step in amyloid formation.^614^ By maintaining the stability of the TTR tetramer, tafamidis reduces amyloid deposition, thereby slowing the progression of the disease and improving clinical outcomes in patients with familial amyloid polyneuropathy and cardiomyopathy.^592^

Another promising development in the field of molecular chaperones is the design of specific compounds that stabilize normal protein conformations to prevent misfolding. A notable example is a rationally designed molecular chaperone known as MC (N,N’-([cyclohexylmethylene]di-4,1-phenylene)bis(2-[1-pyrrolidinyl]acetamide)).^615^ This compound was engineered through docking simulations, molecular dynamics, and quantum chemical calculations to specifically bind and stabilize PrP^C^. In the context of transmissible spongiform encephalopathies (TSEs), fatal neurodegenerative diseases caused by prion proteins, MC has demonstrated significant therapeutic potential.

In vitro studies have shown that MC not only stabilizes PrP^C^ but also eradicates prions in infected cells, prevents the formation of drug-resistant prion strains, and inhibits the interaction between prions and abnormal aggregates.^615^ In vivo, weekly administration of MC in prion-infected mice prolonged survival, and intravenous administration in macaques infected with bovine TSE slowed disease progression and reduced disease-associated biomarkers in the cerebrospinal fluid. Very recently, MC has been designated as an orphan drug for the treatment of prion disease. These findings highlight the potential of de novo-designed chaperone compounds like MC as therapeutic agents for TSEs and possibly other protein misfolding disorders. As discussed in Section “Computational and theoretical approaches: Simulating folding processes”, with the advent of advanced AI-based tools such as AlphaFold and AlphaProteo, the rational design of molecular chaperones such as MC may become increasingly feasible. As our understanding of protein folding and misfolding mechanisms deepens and as computational methods continue to evolve, the development of new molecular chaperones tailored to prevent or correct misfolding in various diseases holds great promise for the future.

In addition to chemical chaperones that stabilize proteins through direct physicochemical mechanisms, considerable effort has been dedicated to developing pharmacological strategies that enhance the expression or function of endogenous molecular chaperones. One of the most advanced compounds in this category is arimoclomol, a pharmacological coinducer of HSPs. Arimoclomol enhances HSF1 activation, thereby amplifying the HSR under conditions of cellular stress.^616^ This results in increased chaperone expression, which improves protein folding, reduces aggregation, and enhances the clearance of misfolded proteins.

Arimoclomol has shown therapeutic potential in diseases associated with protein misfolding and very recently was approved by the FDA for treating Niemann-Pick type C disease, a lysosomal storage disorder characterized by severe neurological and systemic symptoms.^617^ This marked a significant milestone in the development of HSP-based therapies. Niemann‒Pick type C disease has limited treatment options, making arimoclomol a vital addition to the therapeutic arsenal. By amplifying the HSR and enhancing HSP70 expression, arimoclomol facilitates the proper folding and activity of enzymes critical for lysosomal function, thereby reducing substrate accumulation and mitigating disease progression.^616^

In addition to its efficacy in treating lysosomal storage disorders, arimoclomol has demonstrated neuroprotective effects in preclinical models of neurodegenerative diseases such as ALS.^618,619^ In transgenic mouse models of ALS, arimoclomol delayed disease onset, improved motor neuron survival, and extended lifespan.^619^ These effects are attributed to its ability to reduce protein aggregation, support protein homeostasis, and enhance cellular stress resilience. Despite promising preclinical outcomes, recently published phase III clinical trial data suggest that compared with placebo, arimoclomol does not significantly improve efficacy outcomes in patients with ALS.^620^ In the ORARIALS-01 trial, which assessed the safety and efficacy of arimoclomol in 239 participants over 76 weeks, no statistically significant differences were observed in the primary outcome, the Combined Assessment of Function and Survival (CAFS) rank score, and mortality between the arimoclomol and placebo groups.

While these results underscore the inherent challenges of translating chaperone-based therapies into clinical success, arimoclomol exemplifies the potential of harnessing the cell’s endogenous proteostasis machinery for therapeutic benefit. Continued advancements in molecular chaperone modulation, patient selection, and combinatorial treatment strategies will likely define the next frontier in targeting protein misfolding diseases.

#### Inhibiting protein aggregation or promoting aggregation

Efforts to modulate protein aggregation have led to a range of candidate compounds aimed at inhibiting oligomerization or facilitating the disaggregation of existing aggregates. While many such agents remain in preclinical development, several have progressed to clinical evaluation, offering potential disease-modifying benefits. This subsection highlights some of the most advanced and promising compounds.

Anle138b (emrusolmin) is a particularly promising small-molecule aggregation inhibitor identified through a high-throughput screen targeting α-synuclein and prion protein oligomerization.^621^ Orally available and brain penetrant, anle138b binds to cavities within fibrils, stabilizing polar interactions and thereby preventing the formation of toxic oligomers. Notably, it is not limited to α-synuclein; preclinical studies indicate that anle138b can also reduce tau and amyloid-β aggregates.^622,623^ In rodent models of PD and MSA, anle138b reduced α-synuclein deposits, preserved dopaminergic neurons, and improved motor function—even when treatment began after symptom onset.^624–626^ Similarly, in tauopathy and amyloid-β models, the compound reduced the aggregate load and improved neuronal survival and cognition.^622,623^ Following encouraging safety data in a phase I trial, anle138b is now poised for phase II efficacy studies in MSA, highlighting its potential as a disease-modifying therapy (NCT04685265; NCT06568237).

Valiltramiprosate (ALZ-801; tramiprosate) is another small-molecule drug that adopts a different strategy for treating amyloid pathology. As an oral agent that is highly penetrant through the blood‒brain barrier, valiltramiprosate binds and stabilizes soluble Aβ monomers, preventing their conversion into toxic oligomers.^627,628^ In a two-year, open-label phase II trial involving APOE4 carriers with early AD, treatment with valiltramiprosate reduced plasma p-tau181 levels, slowed hippocampal atrophy, and maintained cognitive performance.^629^ Owing to its favorable safety profile, this compound is now under evaluation in a phase III trial targeting APOE4 homozygotes, a population with a particularly high amyloid burden.^630^

While valiltramiprosate targets soluble Aβ, CLR01 represents a distinct class of aggregation inhibitors, described as “molecular tweezers” (for a recent review, see^631,632^). The unique mechanism of the compound stems from its ability to bind to lysine residues on misfolded proteins via noncovalent, shape‒complementary interactions. By anchoring to these positively charged side chains, CLR01 disrupts the electrostatic and hydrophobic contacts necessary for oligomer and fibril formation.

Unlike traditional aggregation inhibitors that may target a single protein species, the broad specificity of CLR01 for lysine-rich regions confers the ability to interfere with multiple amyloidogenic proteins, including α-synuclein and tau.^633–635^ Studies have demonstrated that CLR01 not only prevents the initial formation of amyloid fibrils but also remodels existing fibrils into nontoxic, off-pathway oligomers.^636^ CLR01 inhibited Aβ fibrillization, reduced oligomer-associated toxicity, and preserved synaptic function in primary neuronal cultures.^637^ Similarly, in a zebrafish model of PD, CLR01 mitigated α-synuclein-induced neurotoxicity and rescued dopaminergic neurons from degenerative changes.^633^ Moreover, in transgenic mouse models overexpressing human α-synuclein, CLR01 administered at 12 months of age improved motor deficits and reduced the oligomeric α-synuclein burden. When α-synuclein aggregates were injected directly into the striatum or substantia nigra, CLR01 similarly curtailed the pathology.^638^ These findings indicate that CLR01 can confer neuroprotection by directly interfering with pathogenic protein structures.

The final example of a small molecule drug effective against protein aggregation is squalene (ENT-01), which prevents the initial nucleation step of α-synuclein aggregation by competitively binding to negatively charged phospholipids on vesicular membranes.^639,640^ The high affinity of squalamine for these membranes effectively displaces α-synuclein, reducing the local concentration of the protein at the membrane interface and preventing it from adopting aggregation-prone conformations. In biochemical assays, squalamine markedly reduced the formation of α-synuclein fibrils and oligomers, and in cultured human neuroblastoma cells, it substantially diminished oligomer-induced cytotoxicity.^639,641^ Experiments in *C. elegans* expressing human α-synuclein demonstrated that the compound not only decreased aggregation levels but also almost completely reversed the accompanying muscle paralysis. These findings confirm that targeting the early, membrane-dependent nucleation events of α-synuclein aggregation can mitigate the downstream toxicity and functional impairments associated with synucleinopathies. ENT-01, an orally administered formulation of squalamine, has advanced into clinical trials for PD.^642^ Another clinical trial was initiated to explore the ability of the compound to address PD-related dementia; however, this trial was recently withdrawn for unknown reasons (NCT03938922).

#### Antibodies targeting protein aggregates

Owing to the central role and putative etiological contributions of protein aggregates in NDDs—including Aβ and tau aggregates in AD, α-synuclein in PD, and others—there is substantial interest in treatments that directly target these aggregates to promote their clearance or slow their spread. In addition to the small molecule aggregation inhibitors or stabilizers mentioned above, recent developments in this strategy have leveraged monoclonal antibodies (mAbs) that selectively target aggregates. As reviewed extensively elsewhere, the binding of mAbs, to e.g., Aβ, can promote clearance through various mechanisms.^643^ These include direct recognition of the FC region of the antibody by microglia or indirect recognition mediated by the complement system, which leads to subsequent phagocytosis of the resulting complexes.^643^ Lecanemab and donanemab are FDA-approved mAbs that target Aβ in distinct ways, both showing moderate benefits in clinical trials in slowing the rate of cognitive decline versus placebo, as evaluated by CDR-SB and iADRS scores, respectively.^644,645^ Two phase III trials (EMERGE and ENGAGE) on another anti-Aβ mAb, aducanumab, were stopped due to meeting prespecified futility criteria, although it was later recognized that some assumptions of the analysis were violated and inaccurately predicted the final outcomes.^646^ Nevertheless, in the final analysis, EMERGE, but not ENGAGE, met its primary endpoint (and three secondary endpoints), with aducanumab showing beneficial effects on a reduced decline in cognition and function (per CDR-SB).^646^ In addition, both trials reported significant decreases in the amyloid PET and plasma p-tau indices, which is consistent with the findings of previous studies.^646^ Aducanumab is FDA-approved for milder stages of cognitive impairment or AD.^647^

Not all drugs designed after these principles exhibit equally promising results, examples including the first-generation anti-Aβ mAbs solanezumab,^648^ crenazumab,^649^ and bapinuezemab.^650^ Another drug, the α-synuclein-targeting mAb prasinezumab, showed a lack of benefit for PD patients in a phase 2 trial reported in 2022,^651^ although a post hoc analysis suggested a reduced rate of progression of motor symptoms in a subset of patients.^652^ Prasinezumab is currently being evaluated in another trial.^653^ Evidence of research misconduct in work that may have influenced the approval for testing of prasinezumab by a central investigator in this field serves as a wake-up call and reminder of the rigor and scrutiny needed when conducting or reviewing research.^653^

### Strategies targeting cellular stress responses

#### Modulation of the heat shock response

One therapeutic strategy against protein–misfolding disorders is the use of small molecules that increase chaperone expression or activity, thereby bolstering cellular proteostasis. A promising candidate in this context is LA1011, a dihydropyridine derivative identified as an Hsp90 coinducer.^654^ In wild‑type mice, LA1011 improves spatial learning and memory, whereas in APPxPS1 AD models, it reduces neurodegeneration, preserves neuronal populations, increases dendritic spine density, mitigates tau pathology, and decreases the amyloid plaque burden.^654,655^ Structural studies have revealed that LA1011 interacts with the C-terminal domain of Hsp90, potentially disrupting the binding of FKBP51, a co-chaperone implicated in tau hyperphosphorylation.^656^ The precise mechanism of the neuroprotective effects of LA1011, including its impact on Hsp90 client proteins and other co-chaperones, requires further elucidation.

Sulforaphane, a phytochemical found in cruciferous vegetables, also harnesses the heat shock response by upregulating the co-chaperone CHIP.^657,658^ In a triple transgenic mouse model of AD (3 × Tg-AD), oral administration of sulforaphane reduced Aβ and tau levels, including phosphorylated tau, and improved cognitive performance.^657^ In another model involving intracerebroventricular injection of amyloid, 6 days of sulforaphane treatment enhanced cognition without affecting amyloid accumulation.^659^ In another study in which amyloid aggregation was induced by aluminum and galactose treatment, long-term sulforaphane treatment decreased amyloid plaques, preserved cholinergic neurons and enhanced cognitive function.^660,661^ Although not conclusive, these effects were attributed to the upregulation of CHIP and HSP70, leading to enhanced clearance of Aβ and tau.^657^

An alternative strategy to enhance chaperone expression is to release HSF1 from its inhibitory complex with HSP90.^662^ In various neurodegenerative disease models, this approach reduces misfolded protein aggregates, including pathological tau species.^662–664^ Recent studies revealed that HSP90 can subtly reshape the conformation of Tau, shifting it toward an open state that favors the formation of small oligomers while simultaneously inhibiting fibrillization.^665^ Although this approach might help in preventing large insoluble aggregates, it paradoxically supports a dynamic pool of toxic oligomers that evade normal clearance mechanisms. Moreover, the Hsp70/Hsp90 multichaperone complex can bind and hold pathologically phosphorylated Tau species, maintaining them in a soluble but misfolded state that may accumulate over time, potentially contributing to disease progression.^666^

Despite these insights, the clinical translation of early-generation HSP90 inhibitors has been hampered by systemic toxicity and insufficient CNS penetration.^662^ While some of these compounds reduce tau aggregation and improve neuronal viability in short-term applications, achieving long-term safety and efficacy is challenging.^667^ To address these limitations, recent research has focused on more refined strategies. One promising approach involves the use of nanoparticles to deliver HSP90 inhibitors.^668^ In parallel, the development of OS47720, a CNS-permeable and comparatively safe HSP90 inhibitor, has expanded the therapeutic horizon.^663^ Prolonged (months-long) administration of OS47720 in AD-like mouse models not only induces a beneficial heat shock response but also preserves synaptic structure and function, offering a more viable and enduring therapeutic route for combating tauopathy-driven neurodegeneration.

Beyond direct pharmacological modulators, the development of HSP-inspired nanochaperones represents a next-generation concept.^669,670^ Inspired by natural chaperones, these synthetic nanoassemblies provide a platform to selectively bind misfolded proteins, suppress their aggregation, and facilitate clearance by promoting their uptake by microglia.^669^ Although still at an early stage, such nanochaperones have shown efficacy in preclinical models of AD by reducing Aβ aggregates and improving cognitive function. These systems represent a versatile strategy, potentially customizable to target specific aggregate types while minimally impacting protein homeostasis.

A recent study introduced a novel nanochaperone-mediated autophagy (NCMA) strategy aimed at selectively targeting and eliminating pathogenic tau and its aggregates.^671^ This approach utilizes a customized nanochaperone, Beclin1-VQIINK-nChap (BVNC), which is composed of a micelle with hydrophobic microdomains, tau-binding VQIINK peptides, and Beclin 1 peptides that activate autophagy. BVNC specifically targets and binds pathogenic tau, stabilizing microtubules and maintaining tau homeostasis. The Beclin 1 peptides induce autophagy, forming autophagosomes around the bound tau. These autophagosomes then fuse with lysosomes, enhancing autophagic flux and promoting tau degradation. This innovative NCMA-based approach has been shown to be effective in preclinical AD models by reducing the tau burden and significantly improving cognitive function, representing a promising therapeutic strategy for neurodegenerative diseases.

The translational potential of nanochaperone-based therapies will depend on careful optimization of their size, surface chemistry, biodistribution, and safety profiles.^670^ Questions surrounding long-term biocompatibility, brain penetration, and immunogenicity must be addressed before clinical applications. Nevertheless, the rational design principles underpinning nanochaperones—modularity, specificity, and biocompatibility—mark an exciting frontier in the pharmacological manipulation of cellular stress responses.

#### Targeting the unfolded protein response

Chronic UPR activation is also a hallmark of neurodegenerative disorders, causing proteostasis imbalance and neuronal loss, and targeting UPR pathways is a potential therapeutic strategy. Here, we highlight recent advancements in UPR modulation for the treatment of neurodegenerative diseases.

As summarized above, the PERK‒eIF2α axis of the UPR plays a central role in translational control during ER stress.^672^ In many neurodegenerative diseases, including AD, PD, and ALS, increased phosphorylation of PERK and eIF2α is consistently observed in the brains of patients.^673^ Prolonged PERK activation suppresses global protein synthesis, leading to synaptic failure and neuronal death^674^ These disruptions are central to the pathology of neurodegeneration, as demonstrated in animal models.^673^

Experimental approaches have highlighted the therapeutic potential of modulating PERK signaling. Genetic interventions have successfully mitigated synaptic failure and neuronal loss in AD and prion-diseased mice, with notable survival improvements in certain cases.^674–676^ Similarly, pharmacological inhibitors of PERK, such as GSK2606414, restored protein synthesis and prevented neuronal degeneration in preclinical models of prion disease, tauopathy, PD, ALS, and FTD.^677–680^ Despite these encouraging results, systemic inhibition of PERK is associated with significant on-target pancreatic toxicity, thus presenting a major challenge for clinical translation.^681^

To address this limitation, alternative inhibition strategies have been investigated.^681,682^ The small molecule ISRIB (Integrated Stress Response Inhibitor) binds eIF2B and enhances translation downstream of eIF2α phosphorylation without altering PERK activity.^682,683^ In wild-type mice, ISRIB administration improved memory performance in behavioral tests.^682^ In animal models of neurodegeneration, ISRIB has shown significant neuroprotective effects.^673,684^ Particularly in prion-infected mice, the administration of ISRIB led to a complete absence (0/12) of clinical signs of prion disease, in contrast with the vehicle-treated control group, in which all the mice (9/9) developed confirmatory signs.^673^ In this study, ISRIB not only prevented neuronal loss in the hippocampus but also reduced typical spongiform pathology associated with prion diseases, demonstrating its potential efficacy in mitigating neurodegeneration. ISRIB significantly prolonged the survival of prion-infected mice without causing pancreatic toxicity; however, the authors noted that ISRIB-treated animals were sacrificed because they lost 20% of their body weight despite a lack of clinical or neurodegenerative signs of prion disease, potentially due to the off-target effects of ISRIB.

On the basis of this unfavorable pharmacodynamic profile of ISRIB, the same group performed a compound library screen and identified trazodone, an antidepressant, and dibenzoylmethane (DBM), a naturally occurring compound known for its anti-inflammatory and anticancer properties, as two promising inhibitors of eIF2α phosphorylation (eIF2α-P).^685^ In prion-diseased mice, treatment with either trazodone or DBM effectively restored memory function, prevented the progression of neurological symptoms, and reduced neurodegeneration, significantly extending survival. Similarly, in tauopathy-frontotemporal dementia models, both compounds showed neuroprotective effects, reversing memory impairments and reducing hippocampal atrophy. Additionally, trazodone lowered phosphorylated Tau levels, further highlighting its therapeutic potential. In a recent study in which noncanonical amino acid tagging was used, only two weeks of trazodone treatment restored the hippocampal translatome to a profile comparable to that of healthy mice, with significant recovery of synaptic and mitochondrial protein levels.^686^ This recovery was paralleled by the restoration of synaptic and mitochondrial function, as well as a reversal of their disease-associated decline.

Despite these promising findings, the efficacy of trazodone in clinical settings has raised concerns.^687^ Previous trials investigating the use of trazodone to treat sleep disturbances in Alzheimer’s patients reported no significant benefits, which could be due to its low dose (50 mg) and short treatment duration.^688,689^ Furthermore, the sedative effects of trazodone may not overlap with its ability to modulate the UPR, complicating the interpretation of these results. These trials primarily involved patients with moderate to severe AD, a stage that may be less responsive to such treatments; thus, there may still be potential for trazodone to benefit early or prodromal stages of neurodegeneration, which should be tested by dose optimization and patient stratification in future clinical trials.^689^

Since eIF2α phosphorylation is a central event in the ISR, which is regulated not only by PERK but also by other stress-sensing kinases, therapeutic targeting of eIF2α-P could unintentionally disrupt multiple essential cellular processes. SC79, an AKT activator, was proposed to lower eIF2α-P levels without imposing broad ISR inhibition.^681^ Activated AKT enhances the phosphorylation of Thr799 within the kinase insert loop of PERK and subsequently reduces its ability to bind and phosphorylate eIF2α. In prion-diseased mice, SC79 effectively reduced eIF2α-P levels, providing significant neuroprotection and extending survival. In the 5xFAD mouse model, oral SC79 treatment enhanced memory retention and reduced anxiety without affecting amyloid-β levels.^690^ While SC79 itself may not be suitable for clinical use owing to its own limitations, as a proof-of-principle tool, it highlights the potential of selectively modulating PERK activity as a therapeutic strategy.

In addition to PERK signaling, the IRE1α–XBP1 axis has emerged as a critical player in neurodegenerative diseases, displaying both protective and detrimental effects depending on the context.^691^ The overexpression of spliced XBP1s through AAV-based gene delivery has demonstrated substantial neuroprotection in rodent models of AD, HD, and PD.^692–695^ In these settings, enhancing XBP1s expression appears to alleviate ER stress, restore proteostasis, and preserve neuronal integrity. For example, in mouse models of PD, XBP1s expression in the substantia nigra protects dopaminergic neurons against neurotoxin-induced degeneration, while in HD models, it reduces protein aggregation in the striatum.^692–694^ Similarly, in mouse and *C. elegans* models, XBP1s mitigates amyloid-β or tau toxicity.^696,697^ Moreover, evidence also suggests that XBP1s expression during brain aging preserves synaptic plasticity, prevents cognitive decline, and normalizes proteomic alterations associated with neurodegenerative pathology.^698^ Taken together, several lines of evidence suggest that enhancing IRE1α-XBP1s signaling promotes adaptive responses that can mitigate neurodegenerative pathology.

However, this protective paradigm is complicated by findings that reducing IRE1α–XBP1 signaling can unexpectedly confer neuroprotection in certain contexts. For example, conditional genetic deletion of Xbp1 in mouse models of ALS and HD produced beneficial effects.^699,700^ Instead of exacerbating disease, the loss of Xbp1 enhanced autophagy, facilitating the clearance of misfolded proteins such as mutant SOD1 aggregates in ALS and mutant huntingtin aggregates in HD. Similarly, in models of AD, targeting IRE1 improved cognitive outcomes.^701^ Recently, mesencephalic astrocyte-derived neurotrophic factor (MANF)-mediated inhibition of IREα signaling was reported to protect neurons in an animal model of PD from ER stress-induced death.^702^ These results emphasize the complex interplay between UPR branches and cellular degradative pathways, suggesting that the therapeutic manipulation of IRE1α–XBP1s signaling may need to be finely tuned. In some disease states, blunting IRE1α–XBP1s activity appears to shift the cell toward alternative protective responses, such as autophagy, that can more effectively handle the buildup of toxic aggregates.

While preclinical findings suggest that the IRE1α–XBP1s axis can act as a double-edged sword in neurodegenerative diseases, translating these insights into clinical applications remains challenging. Despite encouraging data, only a few inhibitors of the IRE1α–XBP1s pathway have been tested in vivo. For example, only one study from nearly a decade ago reported a modest but significant neuroprotective effect of KIRA6, an allosteric IRE1α inhibitor, in retinal degeneration models.^703^ Moreover, the long-term consequences of altering UPR pathways in chronic neurodegenerative disorders are still unknown; thus, the possibility of off-target effects must be carefully weighed, given the wide-ranging physiological roles of UPR signaling. Nonetheless, the growing arsenal of small molecules and gene therapy strategies offers hope for selectively targeting IRE1α–XBP1s to mitigate neurodegeneration without disrupting essential cellular homeostasis.

#### Targeting autophagy and the ubiquitin‒proteasome system

As reviewed above, when protein folding issues occur in the cell, leading to the formation of various types of aggregates, one pathway for recovery is autophagy. There is compelling evidence for the importance of autophagy in major diseases such as cancer, neurodegenerative and cardiovascular diseases, as well as aging; thus, autophagy has received substantial attention as a possible therapeutic target or point of intervention.^704,705^

Owing to the many molecular components constituting the autophagy–lysosome pathway and the large number of pathways and mechanisms that regulate autophagy, multiple therapeutic strategies have been explored, some of them making it to clinical trials. Moreover, while increased autophagy is generally regarded as beneficial in the prevention of, e.g., age-related tissue dyshomeostasis and neurodegenerative diseases, the reverse is often true in cancer. Across different tumor types, autophagy supports sustained metabolism, growth, and resistance to therapy in cancer cells.^706^ As such, there is a need to target autophagy in different ways depending on the context; here, we focus on therapeutic strategies that aim to activate autophagy to ameliorate dysproteostasis. There are other recent reviews on autophagy-inhibiting strategies in cancer (e.g.^706).^ This section will present some of the different autophagy-targeting strategies in general terms, along with specific emerging and established interventions that may show promise.

##### Interventions targeting the central autophagy machinery

The central autophagy machinery consists of autophagy receptors and proteins involved in autophagosome formation, as well as upstream kinases and ubiquitin ligases that mark cargo for degradation. In vivo studies have demonstrated that targeting individual genes within this machinery can significantly influence autophagic flux, tissue homeostasis, and overall organismal health.^707^ For instance, the overexpression of Atg5, a key protein for autophagosome formation, enhances autophagy, reduces obesity, improves insulin sensitivity, and extends lifespan in mice.^708^ In a mouse model of AD, lentiviral expression of Becn1, a core component of the autophagy initiation complex, reduced both intracellular and extracellular Aβ accumulation.^709^ Moreover, a specific Becn1 mutation that reduces its sequestration by Bcl2 has been shown to enhance autophagic flux, mitigate age-related phenotypes, and prolong lifespan in mice.^710^ These findings suggest that gene therapies could be viable options, especially in disease cases linked to variants of central autophagy genes.^711^

In addition to the BECN1/BCL2 example, BECN1 sequestration may also be relieved by small-molecule BCL2 inhibitors that selectively target the BECN1 interaction over those of BCL2 with proapoptotic proteins.^712^ Other small pharmaceutical molecules acting at the autophagy machinery include autophagy-targeting chimeras (AUTACs), which can be designed to target specific proteins via guanine-based degradation tags.^713^ AUTACs employ principles similar to those of proteolysis-targeting chimeras (PROTACs), although the latter leverages the proteasome rather than autophagy to mediate targeted protein degradation.^713^

##### Therapeutically targeting lysosomal function

Downstream of the recognition, capture, and lysosomal transfer of cargo by the central autophagy machinery, a range of lysosomal proteins that facilitate or mediate the degradation of this cargo are also potential targets in autophagy-manipulating therapeutics. LSDs, whose common pathophysiological aspects include lysosomal dysfunction due to the loss of function of lysosomal enzymes, provide good examples of diverse therapeutic strategies aimed at restoring lysosomal function downstream of different defects. LSD treatments may involve hematopoietic stem cell transplantation (HSCT) to replenish active enzymes, small molecule-based substrate reduction therapies (SRTs) to decrease the production of accumulating substrates, pharmacological chaperone therapies that stabilize mutated enzymes, enzyme replacement therapies (ERTs), and gene therapy.^714,715^ For example, as an SRT, miglustat has been used to inhibit glucosylceramide synthase to reduce the lysosomal sphingolipid load and is an approved treatment for Niemann–Pick type C^716^; moreover, miglustat acts as a chaperone for glucocerebrosidase (GBA1),^714^ an enzyme whose loss-of-function variants are linked to both Gaucher disease and PD risk.^613^ In mouse models of PD, GBA1 loss leads to α-synuclein accumulation, and GBA1 overexpression reduces the α-synuclein load.^613^ GBA1 activity can also be restored by the pharmacological chaperone ambroxol, which is currently being tested for its safety and efficacy in PD dementia (PDD) in a phase II trial.^613^

In Hunter syndrome, iduronate-2-sulfatase (IDS) deficiency leads to lysosomal glycosaminoglycan accumulation. For this disease, multiple ERTs, including infusions of recombinant idursulfase beta^717^ and, more recently, pabinafusp alfa, which displays improved blood–brain barrier permeability,^718^ have been evaluated in clinical trials. Although these treatments require weekly infusions and are not curative, they show promise for increasing quality of life and extending life expectancy. Gene therapy may represent an opportunity to further alleviate developmental defects and extend the time to disease onset in LSDs; currently, multiple gene therapies are in clinical trials for different LSDs (for a review, see.^715^ Experimentally, lysosomal function in *GBA1* mutant cells, or other disease models, has also been improved through the restoration of lysosomal pH.^719^ Mutations in the lysosomal polyamine exporter ATP13A2—linked to Kufor-Rakeb syndrome, a juvenile-onset form of PDD—can cause lysosomal alkalinization and decreased autophagosome clearance.^719^ Lysosome-targeted acidic nanoparticles have been shown to restore lysosomal pH and enhance autophagosome clearance in ATP13A2-mutant fibroblasts.^719^ Another review discussed the potential application of therapies that increase the intracellular concentration of cAMP or zinc for lysosomal reacidification.^720^

##### Intervening with autophagy-regulating pathways

Among the multiple signaling pathways implicated in regulating autophagy, the mTOR pathway has received the most attention as a possible therapeutic target. Across multiple model organisms, the longevity-increasing effects of TOR inhibition—either through genetic, pharmacological, or dietary interventions^721^—have been well established and shown to act in concert with the consequent stimulation of autophagy.^704^ Notably, the beneficial effects of TOR inhibition are also attributed to decreased protein synthesis.^704^ Moreover, autophagy has been shown to mediate the beneficial reductions of polyQ-expanded protein aggregates in mouse and fly models of Huntington disease downstream of TOR inhibition.^722^ mTOR inhibitors are clinically approved in different settings, e.g., for immunosuppressive and anticancer purposes.^723^ These inhibitors include rapamycin and its derivatives (e.g., everolimus and temsirolimus), as well as multiple second-generation mTOR inhibitors, which have been reviewed extensively elsewhere.^723,724^ As such, several mTOR-inhibiting drugs that may be leveraged for their autophagy-inducing potential already exist. Autophagy is also successfully activated in vivo through inhibition of the acetyltransferase EP300, abolishing its acetylation of the Raptor (RPTOR) subunit of mTORC1 and thereby inhibiting TOR activity. Among the compounds that exhibit inhibitory effects on EP300 are nordihydroguaiaretic acid (NDGA), C646, and the acetylsalicylate (aspirin) derivative salicylate, although the actions of the latter appear to also be mediated by non-EP300-mediated mechanisms.^725^

An important mediator of the autophagy-enhancing effects of mTOR inhibition is the activation of TFEB, a transcriptional regulator recruited to lysosomes and phosphorylated by mTORC1, leading to its sequestration by 14-3-3 proteins.^704^ In addition, autophagy can be stimulated through the activation of AMPK, which acts antagonistically to mTOR. AMPK activation and downstream autophagy induction may be achieved by metformin or trehalose^726^; the latter also stimulates protein folding as an osmolyte.^727^ Resveratrol, a natural compound found in plants, induces autophagy and may do so through promiscuous effects on multiple targets,^728^ including direct inhibition of mTOR.^729^ While we cannot cover all possible approaches here, there are recent reviews that provide an in-depth overview of additional mTOR-dependent and -independent approaches to target autophagy through its upstream regulatory pathways.^345,711,726^

##### Lifestyle interventions that leverage autophagy

It has long been known that dietary interventions such as caloric restriction may promote human health and longevity; this has recently received increasing attention, with scientific studies documenting tangible effects and characterizing the underlying mechanisms, leading to increased interest by the public at large. In 1934, McCay & Crowell referred to the notion that slower growth increases longevity as an “ancient theory” and recapitulated some of the current literature on the topic, showing, for instance, the inverse correlation between growth rate and longevity in rats—data that have since been corroborated.^730^ Notably, caloric restriction and other diets that aim at mimicking its effects (e.g., intermittent or periodic fasting, ketogenic diets) have been shown to consistently restore or enhance autophagy.^705,730^ Upon caloric restriction, mechanisms that contribute to the activation of autophagy may include increased sirtuin expression and activity, coupled with decreased insulin/IGF1 signaling leading to, e.g., decreased mTORC1 activity.^730^ In general, autophagy may contribute to increasing longevity or slowing age-related health declines through preventing or alleviating cytotoxicity and subsequent tissue/organ degeneration stemming from the accumulation of damaged or dysfunctional molecules and organelles within cells.^731^ For example, autophagy has been described as central for stem cell metabolism and in preventing stem cell senescence, thereby supporting the regenerative mechanisms of certain tissues.^732^

Dietary interventions may also include various modes of supplementation. Among the dietary compounds that are experimentally associated with autophagy induction are spermidine,^733^ EGCG,^734^ resveratrol^735^ and trehalose.^726^ In addition, nicotinamide adenine dinucleotide (NAD) augmentation by, e.g., oral nicotinamide riboside (NR) intake, has been shown to increase the transcription of both proteasomal and lysosomal components and decrease serum and CSF inflammatory cytokine levels in PD patients,^736^ suggesting that it may be explored whether the targeting of age-associated declines in NAD synthesis could ameliorate age-related decreases in protein degradation pathways as well as inflammaging. We have also discussed above how micro- and macronutrient deficiencies may disrupt proteostasis, which may similarly be ameliorated through dietary modifications (Section “Genotoxic or proteotoxic stresses and deficiencies”). While dietary supplements are widely used and may provide opportunities in bolstering public health through their relative availability, affordability, and noninvasive nature, drawbacks include their potentially promiscuous or poorly characterized effects, issues in isolating and determining the causality of their effects, or their potential under- or overconsumption outside a regulated setting.^737^ Although the links between some of the mentioned dietary interventions, autophagy, and health have been explored in vitro and in model organisms, controlled trials that establish their efficacy in humans are currently lacking. Additional research may lead to a more integral role for these interventions in future efforts to prevent common degenerative diseases associated with lifestyle or aging.

### Protein misfolding, proteostasis and immunotherapy

The last two decades have seen the emergence of immunotherapy as a transformative new avenue for treating diseases; it involves harnessing or modifying the immune system rather than specifically targeting diseased tissue. Immunotherapy approaches have been particularly successful for certain cancers (for reviews, see^738,739^). Nevertheless, challenges exist, such as limited response in some patients owing to tumor heterogeneity and immune evasion mechanisms, development of resistance (primary or acquired), immune-related adverse effects (such as cytokine release syndrome), high cost and the need for sophisticated infrastructure (for a review, see^740^).

Major types of immunotherapies include immune checkpoint inhibitors (ICIs), which block immune checkpoints to reactivate T-cell responses against tumors; CAR-T-cell therapy, in which patient T cells are engineered to express chimeric antigen receptors (CARs) that target, e.g., specific cancer cells; cancer vaccines and cytokines that boost immune responses to eliminate disease; and monoclonal antibodies that target specific antigens to trigger immune-mediated destruction. In the previous sections, we provided examples of monoclonal antibodies that are used in different settings to combat dysproteostasis. Below, we use ICI as an example to show how proteostasis and immunotherapy approaches could be linked and how this could be actionable, with a focus on cancer therapies.

We have observed in the previous sections that protein folding and proteostasis are significantly disrupted in cancer, which often hijacks normal cellular proteostasis mechanisms for survival. Accordingly, a number of approaches that target proteostasis mechanisms are being developed as a means to destabilize tumors. In principle, immune approaches can also target the proteostasis machinery, which can assist in this quest. In addition, dysproteostasis in tumors can generate neoantigens that can facilitate the recognition of cancer cells by the immune system and their destruction.

In the context of cancer, dendritic cells (DCs), which are one type of antigen-presenting cells, and cytotoxic T lymphocytes (CTLs) are two of the most central immune cells involved in antitumor immunity. In response to changes in the TME, such as inflammation, DCs are attracted to tumor sites as part of the innate immune response; they then take up fragments of dead cancer cells or the material excreted from them, digest them into small fragments, and load them onto major histocompatibility complexes I and II. These are then presented at the surface of DCs to CTLs or T helper cells in the lymph nodes. Antitumor responses to particular antigens in tumors are then activated by CTLs.

Cancer cells present neoantigens on their cell surface through MHC-I complexes, and the ability of CTLs to recognize these foreign peptides is, in the context of ICI, evaluated to be directly proportional to the tumor mutational burden.^741^ Since loss of MHC-I complexes increases the vulnerability of cancer cells to depletion by NK cells, tumors evolve ways to evade immune detection and inhibit immune function in the TME. The prime example of this is alteration in the expression of checkpoint inhibitor proteins, such as programmed death ligand 1 (PD-L1) and Cytotoxic T Lymphocyte-Associated protein 4 (CTLA-4) (for a review, see^742^). PD-L1 is a transmembrane protein normally expressed on the surface of some macrophages, DCs, and activated T and B cells. It binds to another transmembrane cell surface protein, programmed death 1 (PD-1), which is specifically expressed on CTLs. The interactions between PD-L1 and PD-1 normally help prevent immune-mediated tissue damage upon activation. On the other hand, CTLA-4 is normally expressed on regulatory T cells, contributing to their inhibitory function, and its expression increases in T cells upon activation. CTLA-4 is homologous to the T-cell costimulatory protein CD28, both of which bind to B7 on antigen-presenting cells. Whereas the binding of CD28 to B7 is costimulatory, the binding of CTLA-4, which is more avid than that of CD28, is coinhibitory.

Tumor cells express PD-L1 to engage PD-1 to inhibit CTL function. Thus, monoclonal antibodies have been developed to inhibit this interaction, targeting either PD-1 (e.g., nivolumab, pembrolizumab) or PD-L1 (e.g., avelumab, atezolizumab) to restore CTL function and antitumor immunity. Similarly, since CTLA-4 directly inhibits CTL function, antagonistic antibodies against CTLA-4 have been developed to inhibit its interaction with B7 to activate antitumor immunity (e.g., ipilimumab). In the last two decades, these checkpoint inhibitors have transformed cancer therapy for some cancer types (for reviews, see^742^). New checkpoints have also been identified and are now being explored for ICI. However, as noted above, significant hurdles exist, and currently, only approximately 13% of cancer patients benefit from ICI therapies.^743^

Recent studies have shown that different aspects of protein folding and the proteostasis network significantly influence the expression of the checkpoint proteins PD-L1 and CTLA-4. For example, BiP is necessary for the stability and trafficking of PD-L1.^744^ In fact, increased BiP expression through UPR activation is one way by which tumors increase the surface accumulation of PD-L1. Other chaperones, such as HSP90, may also be involved in PD-L1 expression in tumor cells.^745^ An HSP90 inhibitor, AUY-922, induces HSPA8 expression, which competes with CMTM6 for binding to PD-L1 and facilitates its lysosomal degradation to inhibit tumor growth and enhance the antitumor efficacy of anti-PD-L1 and anti-CTLA-4 treatment in BCa.^746^ HSP90 appears to play additional roles in maintaining an immunosuppressive phenotype, as a low level of HSP90 inhibition, which is not sufficient to inhibit the HSR or UPR, or affect PD-L1 expression or localization, increases MHC-I presentation at the cell surface.^747^ Consistent with its role in activating the immune response, this low level of HSP90 inhibition resulted in a lower tumor burden in animal models. These findings suggest that the protein folding machinery and the chaperone system may play important roles in shaping the immunopeptidome and thus the overall immune response.

ER stress and chronic UPR activation can also upregulate PD-L1 expression in immunosuppressive aspects of the TME. For example, upon Kaposi’s sarcoma-associated herpesvirus (*KSHV*) infection of macrophages, the IRE1α-XBP1 pathway is activated and increases the expression of PD-L1, potentially impairing the immune response.^748^ Similarly, in melanoma, activation of the IRE1α‒XBP1 pathway increases macrophage PD-L1 expression and promotes tumor immune evasion.^749^ In addition, ER stress may stimulate the exosomal release of miR-23a by liver cancer cells, leading to the downstream upregulation of PD-L1 expression in macrophages through the miR-23a–PTEN–AKT pathway, thereby promoting tumor progression.^750^ These studies show that ER stress-mediated PD-L1 expression in macrophages can inhibit T-cell function and promote tumor cell escape from antitumor immunity.

Recent studies have provided examples of how targeting key proteostasis regulators can sensitize tumors to ICIs in cancer types that are known not to respond to them (i.e., “cold” tumors, such as cancers of the prostate, ovary, and pancreas). For example, as noted above (Section 4.3.2), we have recently shown that genetic or small-molecule inhibition of IRE1α in syngeneic and orthotopic mouse models of PCa decreased tumor growth, accompanied by potentiation of interferon responses and activation of immune system-related pathways in the TME.^566^ The small-molecule IRE1α inhibitor MKC8866, which is currently in clinical trials, reprogrammed the TME and enhanced anti-PD-1 therapy. These results show that IRE1α signaling, a key player in the overall proteostasis network, interferes with antitumor immunity in the TME, making it a promising target for improving anti-PD-1 immunotherapy in PCa. Interestingly, recent work has shown that targeting IRE1α signaling can increase ICI efficacy in ovarian, breast, and colon cancer models as well, suggesting that it may have broad utility as a combination therapy with ICIs.^579,751,752^ However, the molecular mechanisms by which IRE1α co-targeting with ICIs affects different cancer types vary, and further work is needed to understand the basis of these differences.

Another area of functional intersection of protein folding, proteostasis and ICI resistance is the established link between HSPA1A and HSPA1B expression in CD4^+^ /CD8^+^ T cells and ICI resistance across various cancers.^753^ In this interesting work, a single-cell atlas of T cells from 308,048 transcriptomes across 16 cancer types was established, resulting in the identification of a previously undescribed T-cell type, T Stress Response (TSTR) cells, with a unique stress response state. HSPA1A and HSPA1B expression in TSTR cells in the TME significantly increased following ICI treatment, especially in nonresponsive tumors; these findings suggest that TSTR cells and HSP expression may promote resistance to immunotherapy. Interestingly, in our data on IRE1α inhibition in conjunction with ICI treatment described above, MKC8866 + anti-PD-1 therapy reduced HSPA1A and HSPA1B expression in CD8^+^ T cells and increased the expression of T-cell activation markers.^566^ In another study, the immune landscape of tumors from metastatic castration-resistant PCa by scRNA-Seq revealed that resistance to ICIs was associated with an increase in the HSP90 - steroid hormone receptor pathway and HSF1 activation.^754^

In summary, these findings suggest that protein folding and the proteostasis state of the cell/tissue may have a major influence on the potential efficacy of ICIs; thus, combining ICIs with proteostasis-targeting therapies may have translational impacts in the clinic. Further work is needed to test this possibility.

## Conclusion and future perspectives

Proteins are central actors in cell biology across the tree of life, each with remarkable versatility and efficiency arising from their intricately folded structures. For over two centuries, a primary goal in protein research has been to understand how a linear polypeptide adopts its native 3D conformation, how folding can be measured experimentally, and how accurately this process can be predicted from sequence alone. Not only this is a key question in basic molecular biology, but also important clinically since protein folding defects and dysproteostasis are linked to many major diseases. As detailed in Sections “Historical perspective and milestone events in protein folding” and “Causes and consequences of protein misfolding” of this Review, complementary experimental and computational approaches have provided major insights into folding mechanics and proteostasis under normal physiology. Leveraging this knowledge, a number of targeted therapies have been established, and many more are in development (Sections “Diseases associated with protein folding defects” and “Therapeutic strategies and progress in protein misfolding and dysproteostasis-related diseases”). However, complex folding and homeostasis networks still pose substantial challenges for developing novel therapies. Recent breakthroughs in computational biology and AI, biophysics, biochemistry, molecular and cell biology, and medicine hold great promise for unprecedented advancements in fundamental science as well as translational applications in the future.

In mammalian cells, protein synthesis is among the most energy-demanding processes. Remarkably, approximately one-third of all newly synthesized proteins are rapidly degraded, often due to folding issues, errors, redundancy, and delays that are intrinsic to protein synthesis regulation. Proteins are also continuously and dynamically modified and transported across cellular compartments in response to changing physiological conditions and intra- or extracellular stresses. As detailed in Sections 3 and 5, without rigorous quality control by molecular chaperones, disaggregases, and degradation machinery, these processes may give rise to a state of dysproteostasis that can either result in cell death or disease. Perturbations causing dysfunction may arise from genetic mutations, environmental stresses, aging, errors in protein synthesis or post-translational modifications, or impairment of the cellular quality control pathways themselves. In response, interconnected stress‒response pathways, including the cytosolic HSR, the UPR, and the UPRmt, act collectively to restore proteostasis.

Looking ahead, several promising approaches are emerging. Advances in deep learning and AI-driven protein structure prediction are expected to further expand our understanding of dynamic folding processes, improve simulations that incorporate PTMs, and aid in predicting multiprotein complexes and interactions. These advances will impact not only fundamental science but also translational efforts, including the rational design of small molecules and therapeutic antibodies and even the engineering of synthetic proteins with customized folding properties.

Here, we have highlighted diseases where protein misfolding and dysproteostasis is involved; in the future, a major focus will be on defining disease-specific mechanisms of protein misfolding that constitute the basis for novel therapies. For example, AD, PD, HD, and ALS all involve the accumulation of misfolded proteins in the nervous system. With the knowledge base and novel tools that are becoming available, rather than focusing on late-stage disease protein aggregates, future therapies will instead focus on preventing early misfolding events and stabilizing native conformations, as well as developing early diagnostic tools. Conversely, in cancer, novel approaches will be developed to inhibit protein folding and increase dysproteostasis by targeting different key pathways for folding, the proteasome, autophagy, the UPR, etc. In metabolic and rare diseases where protein misfolding is the culprit, small molecules that can restore native folding or gene editing approaches are being developed to correct mutations that generate misfolding.

Protein folding and proteostasis research has entered a transformative era driven by technological advances and computational breakthroughs. However, critical controversies and knowledge gaps remain—ranging from incomplete mechanistic models to the complexity of stress responses across compartments. Many of these arise from the gap between in vitro systems and the complex cellular environments in which folding occurs, as well as from assumptions embedded in current computational and mechanistic models. For example, the folding intermediates remain elusive in structural studies. While energy landscape theory and the concept of foldons provide a conceptual framework, direct observation of these transient states is technically challenging. Misfolded and partially folded intermediates are thought to be central to aggregation diseases (e.g., in AD and PD), yet their precise structures, lifespans, and fates are unclear. Moreover, there is debate over the universality of folding pathways. Some proteins appear to follow highly reproducible paths; others fold via multiple routes. Whether the pathway is encoded in sequence, modulated by context, or inherently stochastic remains an open question.

Another area of incomplete knowledge involves molecular chaperones which are essential for maintaining proteostasis. For example, their substrate specificity and functional interplay remain poorly understood. Many client proteins are shared between HSP70, HSP90, and chaperonins, often in sequential or overlapping networks. Interestingly, knockout or depletion of individual chaperones frequently results in partial compensation, raising questions about functional redundancy and backup mechanisms. Moreover, while some chaperones facilitate folding, others perform distinct or additional roles—such as acting as disaggregases or triage factors for degradation. Defining the precise boundaries and switching mechanisms among these roles remains an unresolved challenge.

There are also challenges in the translational route to fundamental discoveries in protein folding and proteostasis research. Pharmacological targeting of proteostasis regulators—such as HSP90 inhibitors, IRE1α inhibitors, or chemical chaperones—has shown promise in preclinical models. However, their clinical efficacy remains limited, and off-target effects are a concern. The problem lies in the pleiotropic nature of these pathways: chaperones and stress regulators are involved in numerous cellular processes beyond protein folding. In addition, the context dependency of these pathways complicates therapeutic design. For example, enhancing HSR may protect against neurodegeneration but could support tumor growth. Conversely, inhibiting the UPR may sensitize cancer cells but induce toxicity in normal secretory tissues. A nuanced understanding of cell type specificity, stress duration, and feedback regulation is essential for refining these strategies.

Addressing these open questions is crucial not only for advancing our understanding of fundamental biology but also for translating insights into effective therapies for diseases characterized by protein misfolding, aggregation, and stress dysregulation. A key direction for future research will be to better define the crosstalk among the diverse pathways governing protein folding and proteostasis. While individual components—such as the UPR in the ER and mitochondria, the HSR in the cytosol, and compartment-specific quality control mechanisms—have been extensively studied, their interconnections remain incompletely understood. Notably, interactions between ER stress signaling and mitochondrial pathways, as well as cross-regulation between the ER UPR and cytosolic HSR, warrant deeper investigation. A more integrated understanding of these networks will not only enrich basic biological knowledge but also lay the groundwork for targeted therapeutic strategies aimed at correcting dysproteostasis.

In summary, substantial progress has been made in elucidating the complex landscape of protein folding and proteostasis; however, continued technological innovation and interdisciplinary collaboration are essential to overcome current limitations. These efforts hold promise for the development of precise diagnostic tools and transformative therapies—whether to preserve protein function in degenerative diseases or to exploit proteostasis vulnerabilities in conditions such as cancer.
